# Blood proteomics of menopause map to brain aging and dementia risk

**DOI:** 10.1038/s41591-026-04648-4

**Published:** 2026-09-22

**Authors:** Madeline Wood Alexander, Jennifer S. Rabin, Michelle Caunca, Allesandra Iadipaolo, Louisa Cornelis, Ria Warrier, Keenan A. Walker, Nina Miolane, Veronica Augustina Bot, Brendan Wood, Hamilton Se-Hwee Oh, Tony Wyss-Coray, Albert Pham, Julia Borger, Valentina Diaz, Emily W. Paolillo, Joel Kramer, Laura Pritschet, Caitlin Taylor, Matthew S. Panizzon, Ramiro Eduardo Rea Reyes, Marisa N. Denkinger, Nicholas J. Ashton, Sterling C. Johnson, Emily G. Jacobs, Rowan Saloner, Kaitlin B. Casaletto

**Affiliations:** 1 Hurvitz Brain Sciences Program, Sunnybrook Research Institute, Toronto, Ontario Canada; 2 Rehabilitation Sciences Institute, Temerty Faculty of Medicine, University of Toronto, Toronto, Ontario Canada; 3 Harquail Centre for Neuromodulation, Sunnybrook Health Sciences Centre, University of Toronto, Toronto, Ontario Canada; 4 Department of Medicine, Division of Neurology, Sunnybrook Health Sciences Centre, University of Toronto, Toronto, Ontario Canada; 5 Department of Neurology, Neurovascular Division, Weill Institute for Neurosciences, University of California, San Francisco, CA USA; 6 Department of Psychological and Brain Sciences, University of California, Santa Barbara, CA USA; 7 Ann S. Bowers Women’s Brain Health Initiative, University of California, Santa Barbara, CA USA; 8 Neuroscience Research Institute, University of California, Santa Barbara, Santa Barbara, CA USA; 9 Department of Physics, University of California, Broida Hall, Santa Barbara, CA USA; 10 Laboratory of Behavioral Neuroscience, National Institute on Aging, Baltimore, MD USA; 11 The Phil and Penny Knight Initiative for Brain Resilience, Stanford University, Stanford, CA USA; 12 Wu Tsai Neurosciences Institute, Stanford University, Stanford, CA USA; 13 Graduate Program in Bioengineering, Stanford University, Stanford, CA USA; 14 Department of Medical Biophysics, University of Toronto, Toronto, Ontario Canada; 15 Nash Family Department of Neuroscience, Icahn School of Medicine at Mount Sinai, New York, NY USA; 16 Brain and Body Research Center of the Friedman Brain Institute, Icahn School of Medicine at Mount Sinai, New York, NY USA; 17 Department of Genetics and Genomic Sciences, Icahn School of Medicine at Mount Sinai, New York, NY USA; 18 Ronald M. Loeb Center for Alzheimer’s Disease, Icahn School of Medicine at Mount Sinai, New York, NY USA; 19 Department of Neurology and Neurological Sciences, Stanford University School of Medicine, Stanford, CA USA; 20 Department of Neurology, Edward and Pearl Fein Memory and Aging Center, Weill Institute for Neurosciences, University of California, San Francisco, CA USA; 21 Department of Psychiatry, University of Pennsylvania, Philadelphia, PA USA; 22 Center for Behavior Genetics of Aging, School of Medicine, University of California, San Diego, La Jolla, CA USA; 23 Wisconsin Alzheimer’s Disease Research Center, School of Medicine and Public Health, University of Wisconsin, Madison, WI USA; 24 Banner Sun Health Research Institute, Sun City, AZ USA; 25 Banner Alzheimer’s Institute, Phoenix, AZ USA; 26 Wisconsin Alzheimer’s Institute, School of Medicine and Public Health, University of Wisconsin, Madison, WI USA

**Keywords:** Proteome informatics, Protein-protein interaction networks, Alzheimer's disease, Alzheimer's disease, Risk factors

## Abstract

Menopause is a hallmark process in biological aging that has been implicated in later neurodegenerative risk, but the pathways underlying this connection remain unclear. Here we used blood proteomics data from several cohorts to identify biological changes associated with menopause and its links to brain aging. In *n* = 80 rigorously staged (STRAW+10) pre-, peri- and postmenopausal women (aged 43–58 years) with serum NULISAseq proteomics, we show that spontaneous menopause is characterized by dysregulation in inflammatory, synaptic, metabolic and Alzheimer’s disease biologic processes, which tracked more strongly with hormones than with age. Validation analyses in age-matched pre-/peri- and postmenopausal women (*n* = 2,814) with plasma Olink proteomics replicated the observed proteomic shifts and revealed broader menopause-related upregulation of inflammatory and catabolic processes plus accelerated organ and cell aging, including brain aging. In four independent cohorts of older women (average age, 60.7–72.1 years; total *n* = 11,925), higher menopause proteomic scores associated consistently with cognitive aging and dementia risk. The molecular signatures of menopause may inform the selection of biomarkers or therapeutic targets for brain health in midlife women.

## Abstract (web summary)

Leveraging proteomic data from nearly 15,000 women across several cohorts, this study found that menopause is associated with hormone-linked molecular changes that correlate with poorer cognitive outcomes later in life and the molecular signatures identified might inform selection of therapeutic targets to improve brain health.

## Main

Accumulating data point towards menopause as a critical inflection point in the aging process^1^. Universal to all midlife people with ovaries, menopause is characterized by a steep decline in ovarian hormones (17β-estradiol, progesterone) and a sustained rise in follicle-stimulating hormone (FSH). These endocrine shifts are associated with widespread multisystem health effects^1–4^ that may be relevant for later brain aging and dementia risk^5–9^. Canonical menopause symptoms, including vasomotor (that is, hot flashes, night sweats), sleep problems and mood and/or cognitive changes, are neurologic in origin, suggesting an acute neural response to the changing endocrine environment^10–12^. A recent review estimated that 26–95% of women experience cognitive symptoms during menopause (for example, word-finding, attentional difficulties) and finds that observational studies show overall subtle performance declines (especially in verbal learning and memory) that fall within normative expectations over the menopause transition^12^. Further, experimental data suggest that hypothalamic aging can precede and may causally trigger ovarian senescence, inducing menopause^13,14^. Together, these findings strongly implicate brain involvement in the menopause cascade.

Menopause may represent a midlife window to understand and intervene on incipient brain aging and dementia risk. Although dementia symptoms typically emerge in the mid-70s, biomarker evidence strongly suggests that the pathophysiology of Alzheimer’s disease (AD) begins decades earlier^15,16^, placing menopause (which occurs at an average age of 51 years^17^) in the coincident timeframe for disease pathogenesis. Increasing evidence has identified earlier and surgical menopause as risk factors for AD^5–8,18^. Furthermore, vasomotor symptoms have been associated with poorer memory performance, white matter hyperintensities and AD biomarkers—hallmarks of early dementia risk^19–21^. These data position menopause as a neurologic transition that offers a framework for understanding early manifestations of brain aging and identifying changes that may harken later-life dementia risk in women.

Despite these emerging links, human research investigating the biological pathways by which menopause influences brain aging is scarce. Limited existing proteomics studies of menopause have identified changes in targets involved in metabolic, vascular and immune processes^22,23^. However, previous studies did not employ rigorous menopause staging or proteomic platforms targeted at brain aging, and no studies have tied the molecular profiles of menopause to cognitive outcomes.

We characterized blood-based biological signals associated with spontaneous menopause in healthy midlife women with a restricted age range (43–58 years) and rigorous menopause staging^24^. Using ultrasensitive nucleic acid linked immuno-sandwich assay (NULISAseq) proteomics^25^, we show that menopause is associated with shifts in inflammatory, synaptic, metabolic and AD biologic processes. These signals tracked more strongly with sex hormones than with age. Proinflammatory proteins were especially upregulated in women with vasomotor symptoms. Validation in a large sample of age-matched pre-/peri- versus postmenopausal women with plasma Olink proteomics replicated the observed menopause-associated proteomic shifts and showed broader upregulation of inflammatory and catabolic processes, including accelerations in multisystem organ and cellular aging. Across four cohorts of older women with average ages of 61–72 years, menopause-related proteomic elevations consistently associated with less favorable cognitive outcomes. Together, these findings highlight reproducible biological signatures of menopause that overlap with molecular drivers of later-life brain health, underscoring menopause as a discovery framework for understanding the earliest manifestations of brain aging and risk and resilience to dementia.

## Results

### Spontaneous menopause cohort

**Fig. 1:**
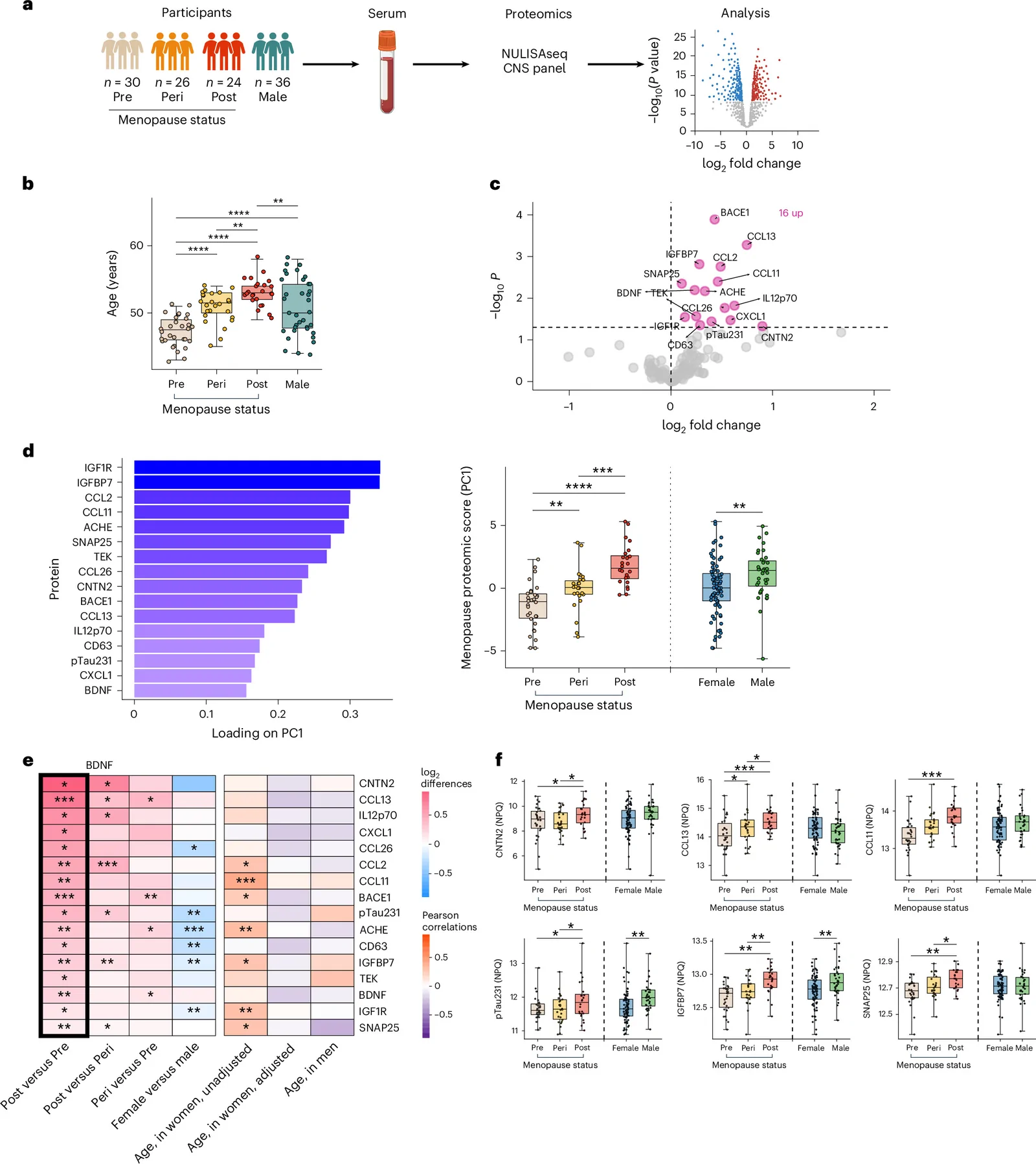
Blood-based proteomics of spontaneous menopause. **a**, Study overview. Blood was collected in 30 premenopausal (pre), 26 perimenopausal (peri) and 24 postmenopausal (post) women, plus 36 men for comparison. Serum was analyzed for 118 proteins using a NULISAseq CNS panel. Age-adjusted analyses identified differences in protein levels across menopause stages and sex. **b**, PCA identified a menopause proteomic score. Participant ages ranged from 43 to 58 years. Despite significant group differences in expected directions, menopause stages exhibited substantial overlap in age. Each datapoint represents an individual participant. The boxplots show the median as the center line, the 25th and 75th percentiles as the bounds of the box and the whiskers as the observed range of the data. Nominal *P* values from two-sided *t*-tests are as follows: pre versus peri *P* = 1.4 × 10^−7^, pre versus post *P* = 1.1 × 10^−13^, peri versus post *P* = 0.001, pre versus male *P* = 5.5 × 10^−5^, peri versus male *P* = 0.82 and post versus male *P* = 0.006. **c**, Age-adjusted ANCOVA with two-sided pairwise contrasts on the estimated marginal means revealed 16 proteins that were upregulated in postmenopausal women relative to premenopausal women, reflecting immune, synaptic, metabolic and neurodegenerative biology. **d**, PCA performed on the 16 proteins from **c** identified a menopause proteomic score (PC1) that increased across menopause stages. The boxplot shows the median as the center line, the 25th and 75th percentiles as the bounds of the box, and the whiskers as the observed range of the data. Each datapoint represents an individual participant. Nominal *P* values from two-sided *t*-tests are as follows: pre versus peri *P* = 0.005, pre versus post *P* = 5 × 10^−9^, peri versus post *P* = 0.0002, female versus male *P* = 0.005. **e**, In age-adjusted analyses, many of the 16 menopause proteins showed significant elevations across menopause stages (pre-, peri-, post-), and some showed sex differences. Several proteins significantly correlated to age in women in bivariate analyses, but these associations with age attenuated and reversed direction after accounting for menopause status. No proteins correlated to age in men. Statistical tests included ANCOVA (menopause stage), linear models (sex) and Pearson correlations (age), and *P* values are nominal. Exact estimates and *P* values are presented in Supplementary Tables 1–3. The black outline denotes the results of main analyses comparing protein levels in post vs. premenopausal women, and additional results reflect post hoc analyses. **f**, Boxplots of top menopause proteins revealed stepwise increases across the menopause transition. The boxplots show the median as the center line, the 25th and 75th percentiles as the bounds of the box, and the whiskers as the observed range of the data, where each datapoint is the observed serum NULISAseq level for an individual participant. Annotations reflect the nominal significance from two-sided, age-adjusted ANCOVA and linear models testing differences in protein levels by menopause stage and sex, and exact statistics are presented in Supplementary Table 1. *P < 0.05, **P < 0.01, ***P < 0.001. Panel **a** created in BioRender; Wood, M. https://biorender.com/9h3qdrj (2026). Source data

We included *n* = 30 premenopausal, *n* = 26 perimenopausal and *n* = 24 postmenopausal women, plus *n* = 36 age-matched men for comparison (Fig. 1a and Table 1). In women, age ranged from 43–58 years, and age differences by menopause stage were statistically significant but small (Fig. 1b). The vast majority were late premenopausal (STRAW+10 stages −3b/−3a), perimenopausal (−2/−1) or early postmenopausal (1a/1b/1c; *n* = 1 postmenopausal woman stage 2; *n* = 1 perimenopausal woman for whom exact stage could not be ascertained). There were no significant age differences by sex and no menopause group differences in body mass index (BMI). Where not included in the text, statistics are presented in Supplementary Tables 1–15.

**Table 1:** UCSB HAS spontaneous menopause cohort characteristics

	Menopause stage				Sex		
	Premenopausal (*n* = 30)	Perimenopausal (*n* = 26)	Postmenopausal (*n* = 24)	*P* value	Female (*n* = 80)	Male (*n* = 36)	*P* value
Age, mean (s.d.) years	47.3 (2.23)^***^	51.0 (2.31)^***^	53.1 (2.02)^***^	1.8 × 10^−14^	50.2 (3.27)	50.8 (4.15)	0.43
Ethnicity, *n* (%)				0.57			0.74
Black/African American	0 (0)	0 (0)	1 (4.2)	–	1 (1.3)	1 (2.8)	–
Filipino	0 (0)	0 (0)	1 (4.2)	–	1 (1.3)	0 (0)	–
Japanese	0 (0)	0 (0)	1 (4.2)	–	1 (1.3)	0 (0)	–
Mexican	1 (3.3)	0 (0)	1 (4.2)	–	2 (2.6)	1 (2.8)	–
Mixed	1 (3.3)	0 (0)	1 (4.2)	–	2 (2.6)	0 (0)	–
Native American/Alaskan	1 (3.3)	0 (0)	0 (0)	–	1 (1.3)	2 (5.6)	–
White	26 (86.7)	24 (100.0)	19 (79.2)	–	69 (88.5)	32 (88.9)	–
White/Chinese/Vietnamese	1 (3.3)	0 (0)	0 (0)	–	1 (1.3)	0 (0)	–
Education, mean (s.d.) years	16.8 (1.71)	16.8 (1.95)	17.1 (2.04)	0.84	16.9 (1.87)^***^	18.1 (1.65)^***^	0.001
BMI, mean (s.d.)	25.5 (4.28)	25.2 (3.33)	25.7 (5.98)	0.93	25.5 (4.58)	26.3 (3.35)	0.32
Smoking history, *n* (%)	6 (20.0)	4 (16.0)	9 (37.5)	0.17	19 (24.1)	7 (19.4)	0.76
High blood pressure or hypertension, *n* (%)	1 (3.3)	0 (0)	0 (0)	0.44	1 (1.3)	3 (8.3)	0.17
High cholesterol, *n* (%)	2 (6.7)	7 (28.0)	6 (25.0)	0.09	15 (19.0)	9 (25.0)	0.63
Diabetes, *n* (%)	1 (3.3)	1 (4.0)	1 (4.2)	0.99	3 (3.8)	2 (5.6)	>0.99
STRAW stage, *n* (%)				–	–	–	–
−3b	9 (30.0)	–	–	–	–	–	–
−3a	21 (70.0)	–	–	–	–	–	–
−2	–	10 (40.0)	–	–	–	–	–
−1	–	15 (60.0)	–	–	–	–	–
1a	–	–	6 (25.0)	–	–	–	–
1b	–	–	6 (25.0)	–	–	–	–
1c	–	–	11 (45.8)	–	–	–	–
2	–	–	1 (4.2)	–	–	–	–
Estradiol (pg ml^−1^), mean (s.d.)	97.7 (95.24)^**^	62.9 (59.32)^**^	9.34 (11.32)^**^	0.0002	61.3 (77.2)	–	–
Progesterone (ng ml^−1^), mean (s.d.)	2.50 (2.94)^*^	1.65 (3.25)^*^	0.60 (0.55)^*^	0.05	1.69 (2.70)	–	–
FSH (mIU ml^−1^), mean (s.d.)	9.18 (7.22)^***^	36.8 (25.5) ^***^	82.2 (34.3)^***^	1.4 × 10^−15^	38.9 (38.0)	–	–
SHBG (nmol l^−1^), mean (s.d.)	65.5 (39.2)	82.9 (47.8)	60.7 (31.6)	0.16	69.6 (40.7)^***^	37.0 (18.6)^***^	1.6 × 10^−^^7^
DHEAS (μg dl^−1^), mean (s.d.)	130.1 (73.4)	122.5 (64.4)	116.8 (52.3)	0.77	123.8 (64.5)^***^	191.6 (73.2)^***^	5.6 × 10^−^^5^
Testosterone (ng dl^−1^), mean (s.d.)	16.7 (10.1)	19.1 (11.2)	14.9 (13.1)	0.47	16.9 (11.3)^***^	479.9 (141.4)^***^	<2.2 × 10^−^^16^
Menopause symptoms in last 2 weeks							
Hot flashes, *n* (%)	–	11 (44.0)**	16 (66.7)**	0.008	–	–	–
Night sweats, *n* (%)	–	16 (64.0)	11 (45.8)	0.09	–	–	–
Vaginal dryness, *n* (%)	–	6 (24.0)	8 (33.3)	0.75	–	–	–
Irritability, *n* (%)	–	19 (76.0)	15 (62.5)	0.56	–	–	–
Unilateral oophorectomy, *n* (%)	0 (0)	0 (0)	2 (8.3)	0.09	1 (1.25)	–	–
Current hormonal contraceptive use, *n* (%)	1 (3.4)	0 (0)	0 (0)	0.43	1 (1.3)	–	–
Past hormone therapy use, *n* (%)	0 (0)	2 (8.0)	0 (0)	0.11	2 (2.6)	–	–

*P* values (*P < 0.05, **P < 0.01, ***P < 0.001) are nominal (unadjusted) and represent the results of ANOVA, *t*-tests and *χ*^2^ tests testing differences by menopause stage and sex.

#### Inflammatory, synaptic and neuropathology-related proteins were elevated in postmenopausal women

We first examined differential serum protein abundance in postmenopausal versus premenopausal women (Fig. 1c). Adjusting for age, 16 proteins were elevated in postmenopausal women, reflecting inflammatory (CCL13, IL-12p70, CXCL1, CCL26, CCL2, CCL11, TEK, CD63), synaptic/neuronal network (CNTN2, ACHE, SNAP25, BDNF), metabolic (IGFBP7, IGF1R), and AD (BACE1, p-tau231) biology (Supplementary Table 1). To reduce data dimensionality and noise, more holistically capture biology represented, and mitigate multiple comparisons, we performed principal components analysis (PCA) on these 16 targets to estimate a menopause proteomic score that summarizes these top menopause-related changes. The first principal component (PC1) explained 28.7% of the variance in menopause proteins (Supplementary Fig. 1), whereby higher values reflected a more postmenopausal-like proteome (Fig. 1d). Confirming this, age-adjusted analyses revealed stepwise increases in PC1 (that is, the menopause proteomic score) across the menopause transition (post (versus peri): *β* = 2.20, 95% confidence interval (CI) = 1.178, 3.21, *P* = 0.00005, peri (versus pre): *β* = 1.87, 95% CI = 0.772, 2.98, *P* = 0.001). We assessed stability of PC1 loadings using bootstrap resampling (1,000 iterations), which showed low s.d. values (<0.08) and high sign consistency (>0.95) across all proteins (Supplementary Fig. 2).

In women, age was associated significantly with higher menopause proteomic scores (correlation = 0.36, *P* = 0.001). However, this association with age was no longer significant and reversed directionality after adjusting for menopause stage (*β* = −0.14, 95% CI = −0.31, 0.03, *P* = 0.11). Unadjusted linear estimates showed a stepwise effect of menopause stage on the proteomic score. Relative to premenopausal, perimenopausal status was associated with a 1.36-unit (s.d.) increase in menopause scores, and postmenopausal status with a 3.25-unit increase. By contrast, each year of age was associated with a 0.24-unit higher menopause score. To assess the potential modulating role of age, we also tested interactions between menopause stage and age on the menopause score, which were not significant (post (versus pre) × age: *β* = 0.003, 95% CI = −0.44, 0.45, *P* = 0.99; peri (versus pre) × age: *β* = 0.17, 95% CI = −0.24, 0.57, *P* = 0.42). We computed the same composite score in men and found age did not correlate to the menopause score in men (cor = −0.02, *P* = 0.89; see Fig. 1e and Supplementary Table 2 for post hoc tests of individual proteins). Overall, these findings suggest that the observed proteomic changes were driven more by menopause stage than by chronological age in women.

**Extended Data Fig. 1:**
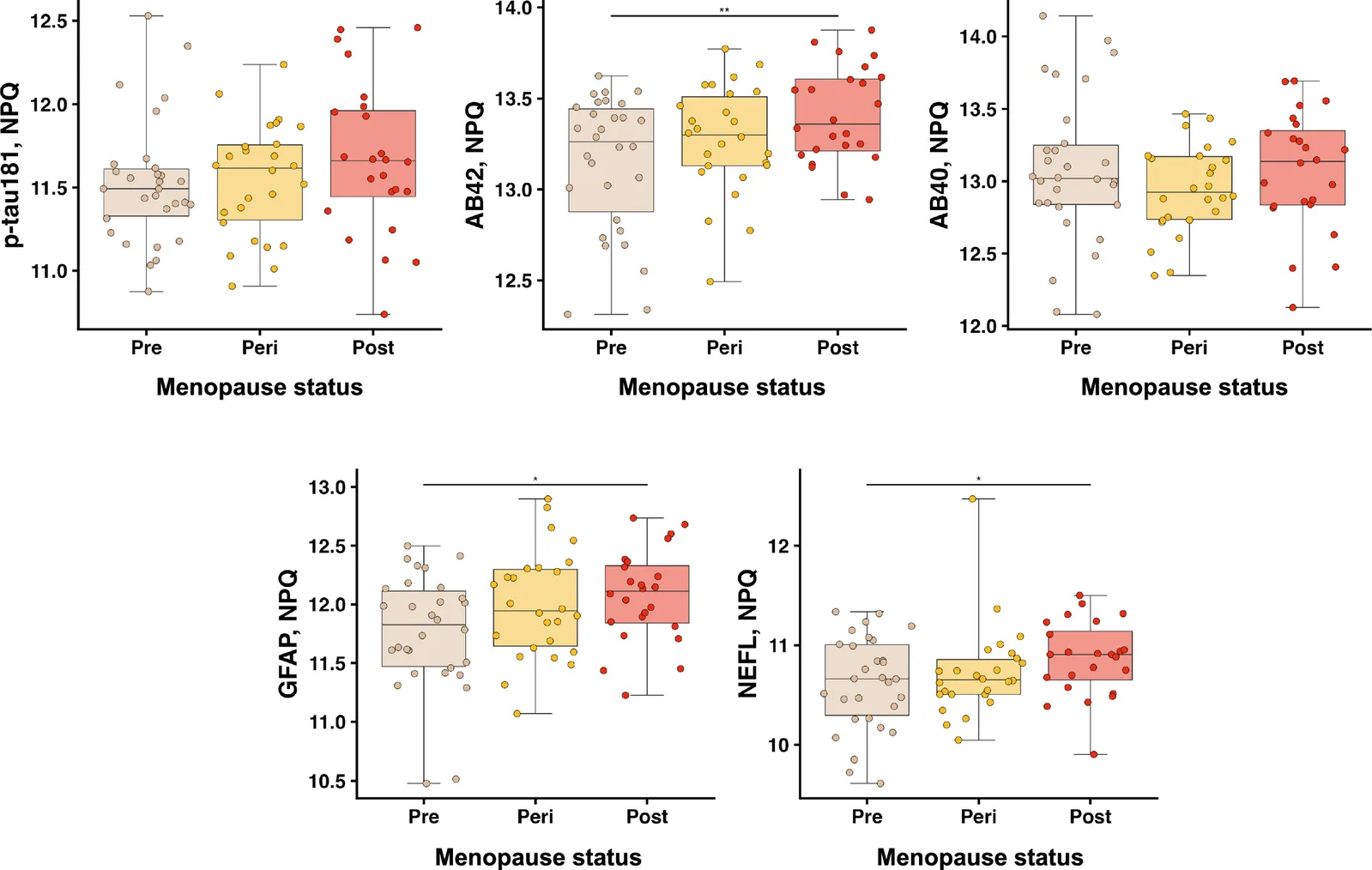
Distributions of plasma biomarkers of neurodegeneration by menopause status. The boxplots depict levels of p-tau181, Aβ40, Aβ42, GFAP and NEFL by menopause stage (*N* = 80 women total; *N* = 30 premenopausal, *N* = 26 perimenopausal, and *N* = 24 postmenopausal). Each datapoint is the NULISAseq serum proteomics level for an individual participant, and the plots show the median as the center line, the 25^th^ and 75^th^ percentiles as the bounds of the box, and the whiskers as the observed range of the data. The significance annotations reflect the results of unadjusted, two-sided t-tests comparing protein level by menopause stage (p-tau181: pre vs. peri *p* = 0.82; pre vs. post *p* = 0.21, peri vs post *p* = 0.29; Aβ42: pre vs. peri *p* = 0.17; pre vs. post *p* = 0.004, peri vs post *p* = 0.10; Aβ40: pre vs. peri *p* = 0.24; pre vs. post *p* = 0.94, peri vs post *p* = 0.24; GFAP: pre vs. peri *p* = 0.08; pre vs. post *p* = 0.02, peri vs post *p* = 0.55; NEFL: pre vs. peri *p* = 0.40; pre vs. post *p* = 0.04, peri vs post *p* = 0.24). In models adjusted for age, none of these canonical AD biomarkers significantly differed between post- and premenopausal women (Supplementary Table 1).

Notably, after accounting for age, p-tau231 was the only canonical AD biomarker (versus p-tau181, Aβ40, Aβ42, GFAP, NEFL) that differed significantly between post- and premenopausal women (Supplementary Table 1 and see Extended Data Fig. 1 for distributions of these biomarkers by menopause stage). Examining sex differences, men had higher ‘menopause proteomic scores’ than women overall adjusting for age (*β* = 1.13, 95% CI = 0.27, 1.90, *P* = 0.009; see Fig. 1f and Supplementary Table 3 for individual proteins).

#### Menopause proteins tracked with estradiol and FSH

**Fig. 2:**
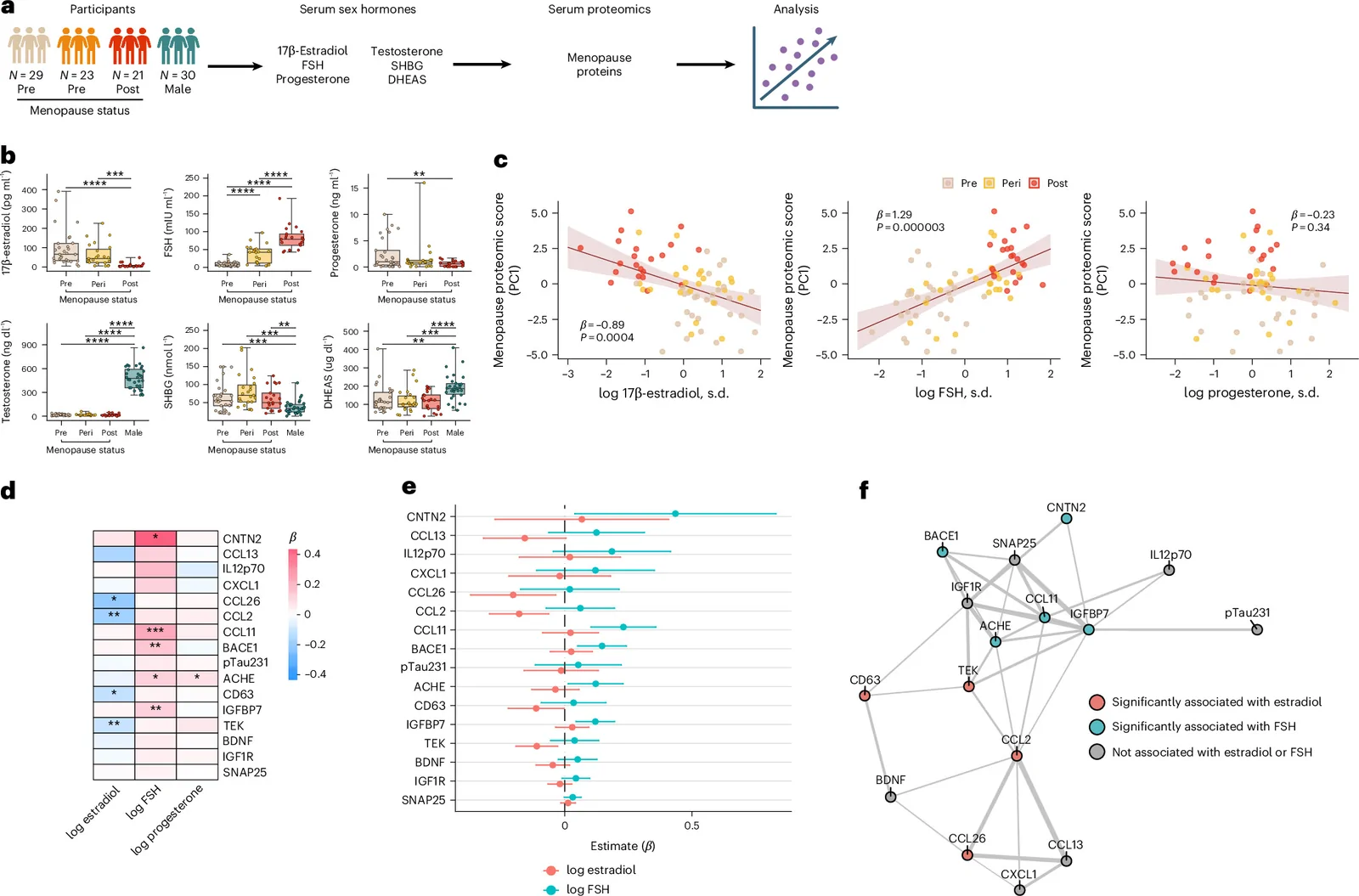
Menopause proteins tracked with estradiol and FSH. **a**, Serum was analyzed for sex hormones: 17β-estradiol, FSH and progesterone (in women only), plus testosterone, SHBG and DHEAS in all participants. Analyses examined associations between menopause proteins and hormone levels. **b**, In ANOVA models, estradiol, FSH and progesterone differed significantly by menopause stage, but testosterone, SHBG and DHEAS did not. The boxplots show the median as the center line, the 25th and 75th percentiles as the bounds of the box and the whiskers as the observed range of the data. Each datapoint represents the observed hormone level in an individual participant. Nominal *P* values from two-sided *t*-tests are as follows: estradiol: pre versus peri *P* = 0.11, pre versus post *P* < 2.9 × 10^−5^, peri versus post *P* = 0.0003. FSH: pre versus peri *P* = 3.5 × 10^−5^, pre versus post *P* = 3.4 × 10^−9^, peri versus post *P* < 1.7 × 10^−5^. Progesterone: pre versus peri *P* = 0.33, pre versus post *P* = 0.002, peri versus post *P* = 0.14. Testosterone: pre versus peri *P* = 0.44, pre versus post *p* = 0.60, peri versus post *P* = 0.26, pre versus male *P* < 2.22 × 10^−16^, peri versus male *P* < 2.22 × 10^−16^, post versus male *P* < 2.22 × 10^−16^. SHBG: pre versus peri *P* = 0.17, pre versus post *P* = 0.64, peri versus post *P* = 0.08, pre versus male *P* = 0.001, peri versus male *P* = 0.0002, post versus male *P* = 0.004. DHEAS: pre versus peri *P* = 0.69, pre versus post *P* = 0.46, peri versus post *P* = 0.75, pre versus male *P* = 0.002, peri versus male *P* = 0.0006, post versus male *P* = 9.2 × 10^−5^. **c**, Age-adjusted linear regression models revealed that estradiol and FSH (but not progesterone) related significantly to the menopause proteomic score. Within each panel, each datapoint represents the menopause proteomic score for an individual participant. Shaded regions: 95% CIs. **d**, In post hoc age-adjusted linear models simultaneously testing associations of hormone levels with individual proteins, FSH was associated uniquely with five proteins, estradiol was associated uniquely with four proteins and progesterone was associated uniquely with one protein. *P* values are nominal and reflect the results of two-sided significance tests. Exact statistics are presented in Supplementary Tables 4. **e**, A forest plot of beta coefficients for estradiol and FSH from age- and progesterone-adjusted linear models showed that menopause proteins demonstrate specificity in their associations with sex hormones. CNTN2, CCL11, BACE1, ACHE and IGFBP7 associated only with FSH, and CCL26, CCL2, CD63 and TEK associated only with estradiol. Error bars: 95% CI. **f**, Network analysis demonstrated that estradiol-associated (CCL13, CCL26, CCL2, TEK) and FSH-related (CNTN2, CCL11, BACE1, ACHE, IGFBP7) proteins clustered into two separate groups. *P < 0.05, **P < 0.01, ***P < 0.001. Panel **a** created in BioRender; Wood, M. https://biorender.com/pvigodb (2026). Source data

Estradiol, FSH and progesterone significantly differed by menopause stage, whereas testosterone, sex-hormone binding globulin (SHBG) and dehydroepiandrosterone sulfate (DHEAS) did not (Table 1 and Fig. 2a,b). Therefore, we focused on the former three in proteomic analyses. Age-adjusted linear models showed the menopause proteomic score was associated inversely with estradiol (*β* = −0.89, 95% CI = −1.37, −0.41, *P* = 0.0004), positively with FSH (*β* = 1.29, 95% CI = 0.78, 1.80, *P* = 0.000003) and not associated with progesterone (*β* = −0.23, 95% CI = −0.71, 0.25, *P* = 0.34; Fig. 2c). When simultaneously modeling all three hormones together, only FSH was associated significantly with the menopause score (FSH: *β* = 1.13, 95% CI = 0.51, 1.78, *P* = 0.0005; estradiol: *β* = −0.43, 95% CI = −0.97, 0.11, *P* = 0.12; progesterone: *β* = 0.24, 95% CI = −0.20, 0.68, *P* = 0.28). Furthermore, in the same multivariable linear regression, age was not associated with the menopause score (*β* = −0.01, 95% CI = −0.17, 0.14, *P* = 0.88), suggesting that the menopause score is related more strongly to hormone levels than to age when modeled together. Post hoc analyses examining individual proteins showed distinct hormone–protein relationships such that CCL26, CCL2, CD63 and TEK tracked inversely with 17β-estradiol; CNTN2, CCL11, BACE1, ACHE and IGFBP7 tracked positively with FSH; and only ACHE tracked with progesterone (Supplementary Table 4 and Fig. 2d,e). A network analysis estimating pairwise correlations at a threshold of *r* = 0.3 illustrated that estradiol-related proteins and FSH-related proteins clustered into two separable groups (Fig. 2f), suggesting that different menopause proteins may reflect distinct hormone-related biology.

#### Women with vasomotor symptoms showed the most pronounced increases in inflammatory menopause proteins

**Fig. 3:**
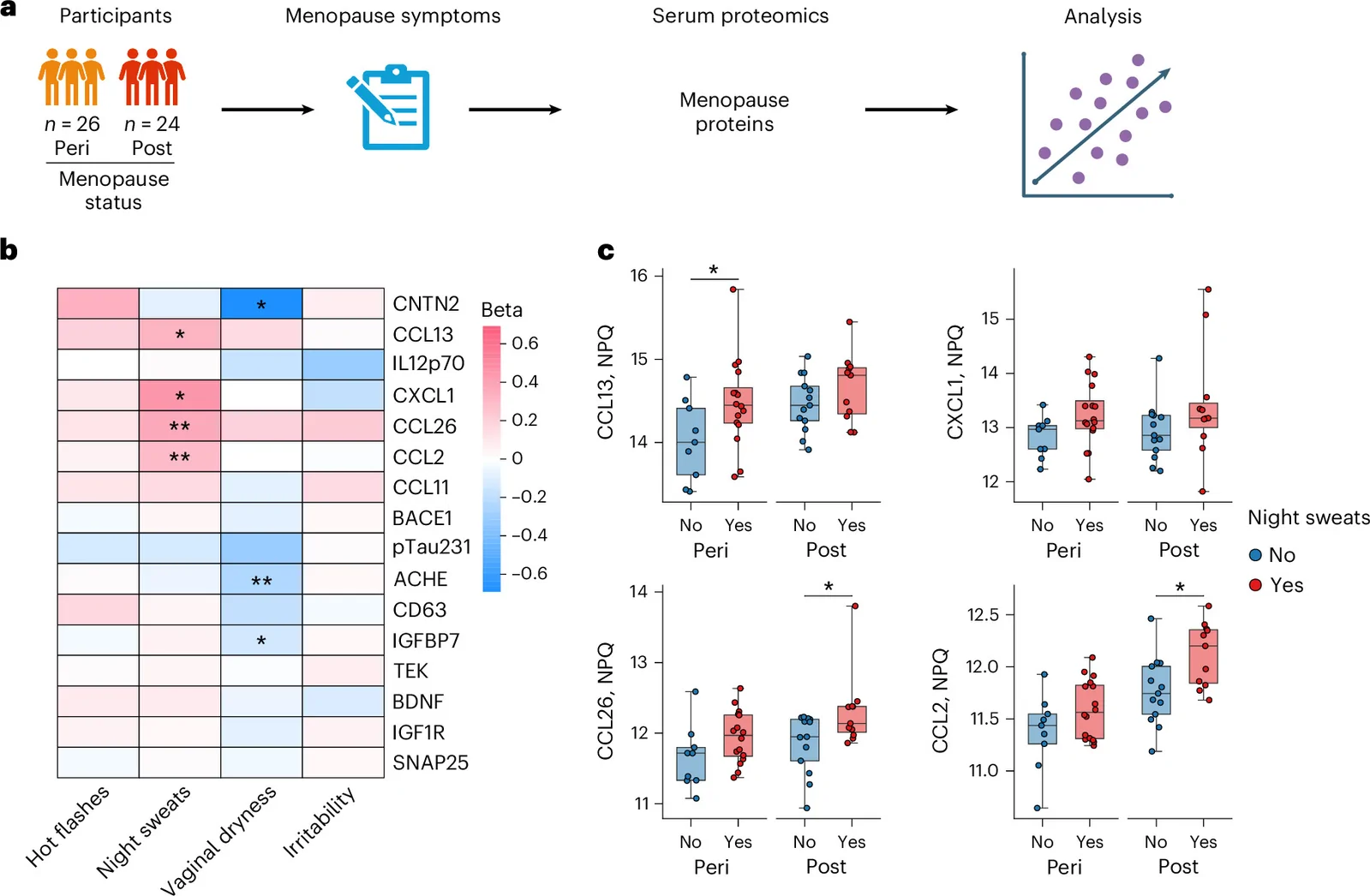
Menopause symptoms associated with menopause protein levels in peri- and postmenopausal women. **a**, Peri- and postmenopausal women self-reported hot flashes, night sweats, vaginal dryness and irritability. Analyses tested associations of menopause symptoms with menopause proteomic scores and individual proteins. **b**, In linear models adjusted for age and menopause stage, women with night sweats had significantly elevated levels of CCL13, CXCL1, CCL26 and CCL2. Conversely, women with vaginal dryness had significantly lower levels of CNTN2, ACHE and IGFBP7. *P* values are nominal and reflect the results of two-sided significance tests. Exact statistics are presented in Supplementary Tables 5. **c**, For the four proteins that showed overall differences in women with versus without night sweats (**b**), boxplots stratified by night sweats status and menopause stage are presented to illustrate the data distribution. Within each panel, each datapoint represents serum NULISAseq level in an individual participant. The boxplot shows the median as the center line, the 25th and 75th percentiles as the bounds of the box, and the whiskers as the observed range of the data. Nominal *P* values from two-sided *t*-tests comparing protein levels in women with versus without night sweats within each menopause stage are as follows: CCL13—peri *P* = 0.04, post *P* = 0.22; CXCL1—peri *P* = 0.05, post *P* = 0.19; CCL26—peri *P* = 0.12, post *P* = 0.035; CCL2—peri *P* = 0.14, post *P* = 0.01. Panel **a** created in BioRender; Wood, M. https://biorender.com/pvigodb (2026).

In peri- and postmenopausal women, we tested whether menopause symptoms were associated with the menopause proteomic score (Fig. 3a). Night sweats were related to higher (more postmenopausal) scores (*β* = 0.94, 95% CI = 0, 1.88, *P* = 0.05), whereas vaginal dryness was associated with lower scores (*β* = −1.15, 95% CI = −2.17, −0.13, *P* = 0.03). There were no associations between hot flashes or irritability and the menopause score (hot flashes: *β* = 0.43, 95% CI = −0.55, 1.42, *P* = 0.38; irritability: *β* = 0.32, 95% CI = −0.74, 1.38, *P* = 0.55). In post hoc analyses, CXCL1, CCL26, CCL13 and CCL2 were upregulated in women with night sweats, whereas CNTN2, ACHE and IGFBP7 were downregulated in women with vaginal dryness (Supplementary Table 5 and Fig. 3b,c).

### Cross-platform menopause validation cohort

#### Menopause proteomic shifts replicate in a large independent cohort

**Fig. 4:**
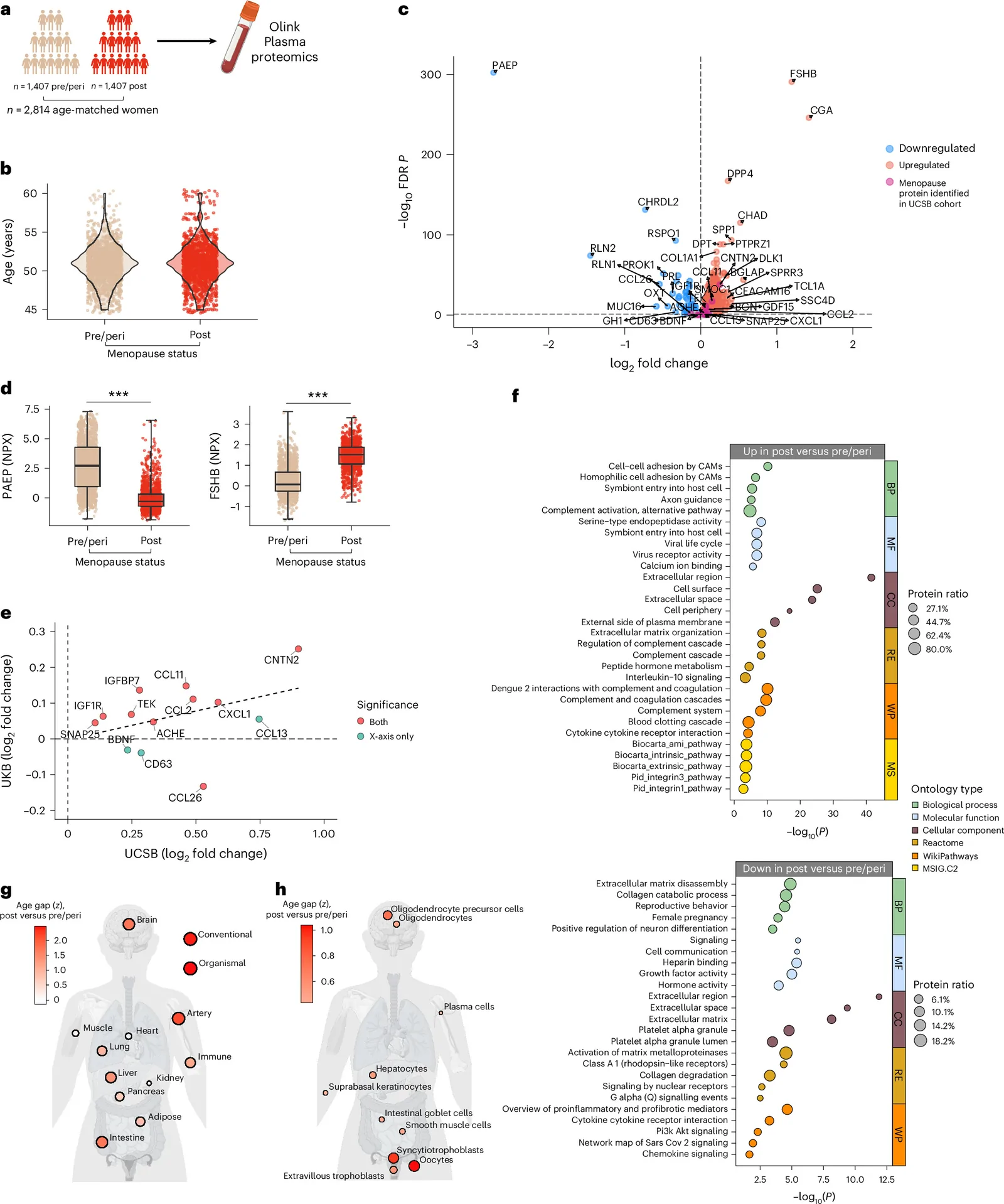
Menopause proteomic shifts replicate cross-platform in age-matched pre-/peri- and postmenopausal women in the UKB. **a**,**b**, Pre-/peri- and postmenopausal women aged 45–60 years were propensity-matched on age with a 1:1 ratio to form the analytic sample (**a**; *n* = 2,814). A two-sided *t*-test showed no differences in age in the matched sample (**b**; *P* = 0.68). **c**, Age-adjusted differential abundance analyses (linear models) of Olink proteins demonstrated 1,146 upregulated and 154 downregulated proteins in postmenopausal (versus pre-/peri-menopausal) women. **d**, Top hits included markers of ovarian hormone signaling, including PAEP and FSHB. The boxplots show the median as the center line, the 25th and 75th percentiles as the bounds of the box, and the whiskers as the observed range of the data. Each datapoint represents the observed Olink protein level in an individual participant. Nominal *P* values from two-sided *t*-tests are as follows: PAEP: pre-/peri- versus post *P* < 2 × 10−, FSHB: pre-/peri- versus post *P* < 2.2 × 10^−16^. **e**, Effect sizes for menopause proteins identified in UCSB demonstrated concordance with those in UKB, showing strong overlap of the largest-magnitude hits in both cohorts. **f**, GO and pathway enrichment analysis of FDR-significant menopause proteins revealed menopause-associated downregulation of proteins enriched for growth factor and reproductive signaling and upregulation of proteins enriched for cytokine signaling, complement activation and extracellular matrix catabolism. The bubble plots show signed enrichment *z*-scores derived from nominal one-sided Fisher’s exact test enrichment statistics. **g**, Body plot showing organ age associations with menopause stage. **h**, Body plot showing the top ten cell aging measures associated with menopause stage. *P < 0.05, **P < 0.01, ***P < 0.001. Panels created in BioRender: **g**, Wood, M. https://biorender.com/mzowo2k (2026); **h**, Wood, M. https://biorender.com/9h3qdrj (2026). Source data

In the UK Biobank (UKB) validation cohort including equal groups of age-matched pre-/peri- and postmenopausal women aged 45–60 years (total *n* = 2,814; Supplementary Table 6), 44% of plasma proteins (1,300 of 2,923 Olink proteins) were differentially abundant (false discovery rate (FDR) *P* < 0.05) by menopause stage (1,146 upregulated in post versus pre-/peri, 154 downregulated in post versus pre-/peri; Fig. 4a–c and Supplementary Table 7). Top hits included markers reflecting ovarian hormone signaling, including decreased progestogen-associated endometrial protein (PAEP) and increased FSH subunits beta (FSHB) and alpha (CGA; Fig. 4d). We assessed whether the specific menopause proteins identified in the University of California Santa Barbara (UCSB) cohort also showed menopause stage differences in the UKB. Of the 13 menopause proteins measured on both proteomic platforms, 9 clearly replicated across cohorts, with effect size concordance (*r* = 0.39) and strong overlap of largest-magnitude hits (Fig. 4e). Replication analyses were robust using the menopause proteomic score as the outcome, tested in a smaller subset of UKB women with nonmissing values on all 13 available menopause proteins (*n* = 1,976; post (versus pre-/peri): *β* = 0.22, 95% CI = 0.13, 0.31, *P* = 0.000001).

**Extended Data Fig. 2:**
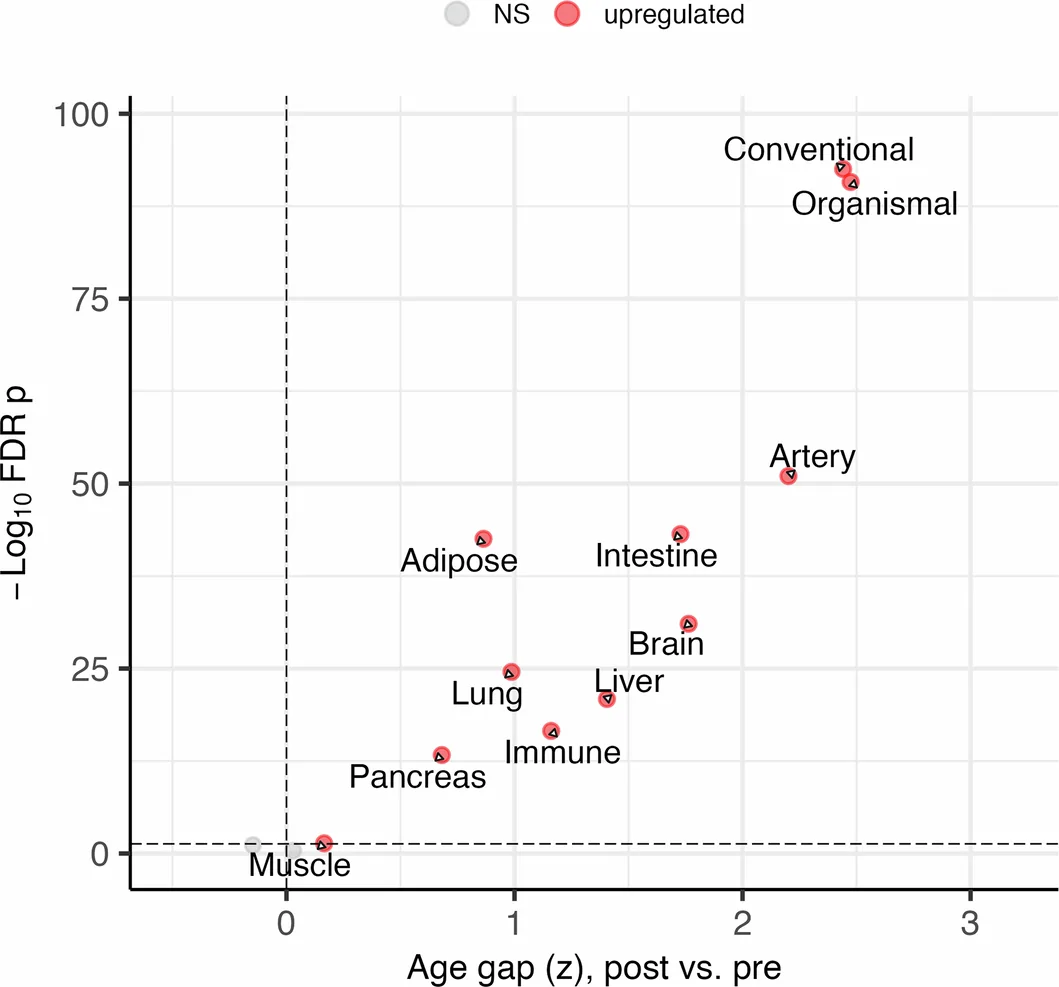
Volcano plot showing organ age gaps in the UK Biobank by menopause stage. The volcano plot depicts organ age gaps (expressed in standard deviations) on Olink proteomic clocks of organ aging, in a sample of *N* = 2,340 age-matched pre/peri- and postmenopausal women from the UK Biobank. Compared to pre/peri-menopausal women of the same age, in age-adjusted linear models, postmenopausal women had larger (that is, less favourable) age gaps on 11 of 13 organ age measures.

**Extended Data Fig. 3:**
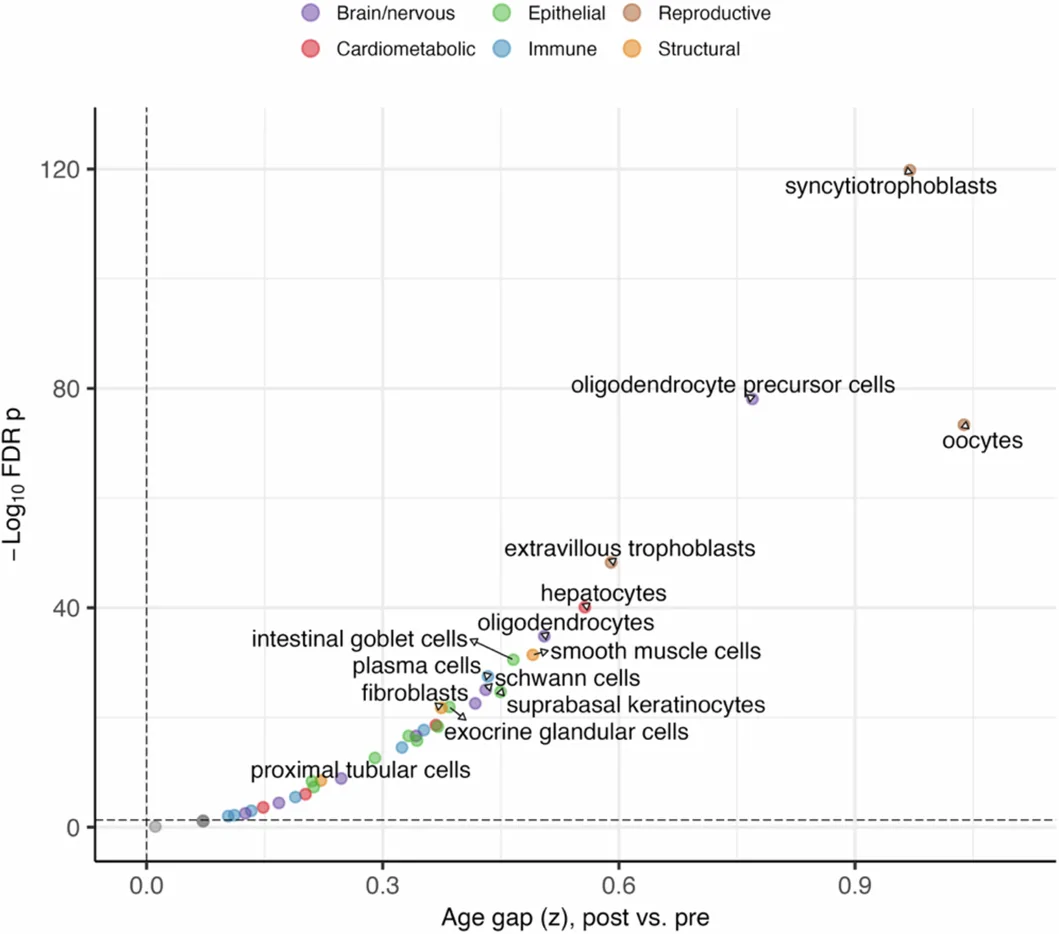
Volcano plot showing cell age gaps in the UK Biobank by menopause stage. The volcano plot depicts cell age gaps (expressed in standard deviations) on Olink proteomic clocks of cell aging, in a sample of *N* = 2,340 age-matched pre/peri- and postmenopausal women from the UK Biobank. Compared to pre/peri-menopausal women of the same age, in age-adjusted linear models, postmenopausal women had larger (that is, less favourable) age gaps on 36 of the 38 cell age measures. FDR-significant cell aging measures are colour-coded by organ system category.

We performed post hoc gene ontology (GO) and pathway analysis on the broader list of 1,300 Olink proteins differentially abundant by menopause stage. These analyses revealed menopause-associated downregulation of proteins enriched for growth factor and reproductive signaling and upregulation of proteins enriched for cytokine signaling, complement activation and extracellular vesicle–related processes (Fig. 4f). Next, in *N* = 2,340 age-matched pre-/peri- and postmenopausal women, we estimated proteomic aging clocks and assessed whether menopause stage was associated with organ and cell aging (Supplementary Table 8). Relative to pre-/peri-menopausal women of the same age, in age-adjusted analyses, postmenopausal women had larger (less favorable) age gaps on 11 of 13 organ age measures (Extended Data Fig. 2) and 36 of the 38 cell age measures (Extended Data Fig. 3). Top menopause-associated organ ages included artery, brain and intestine aging, plus conventional and organismal (overall, organ-shared) aging (Fig. 4g). For cell aging, top measures included female reproductive cells (for example, oocytes, syncytiotrophoblasts), and oligodendrocyte precursor cells, supporting the biological validity of menopause classifications in the UKB and highlighting the potential role of menopause in brain aging (Fig. 4h).

### Aging cohorts

#### Menopause symptoms associated with menopause protein levels in later life

**Extended Data Fig. 4:**
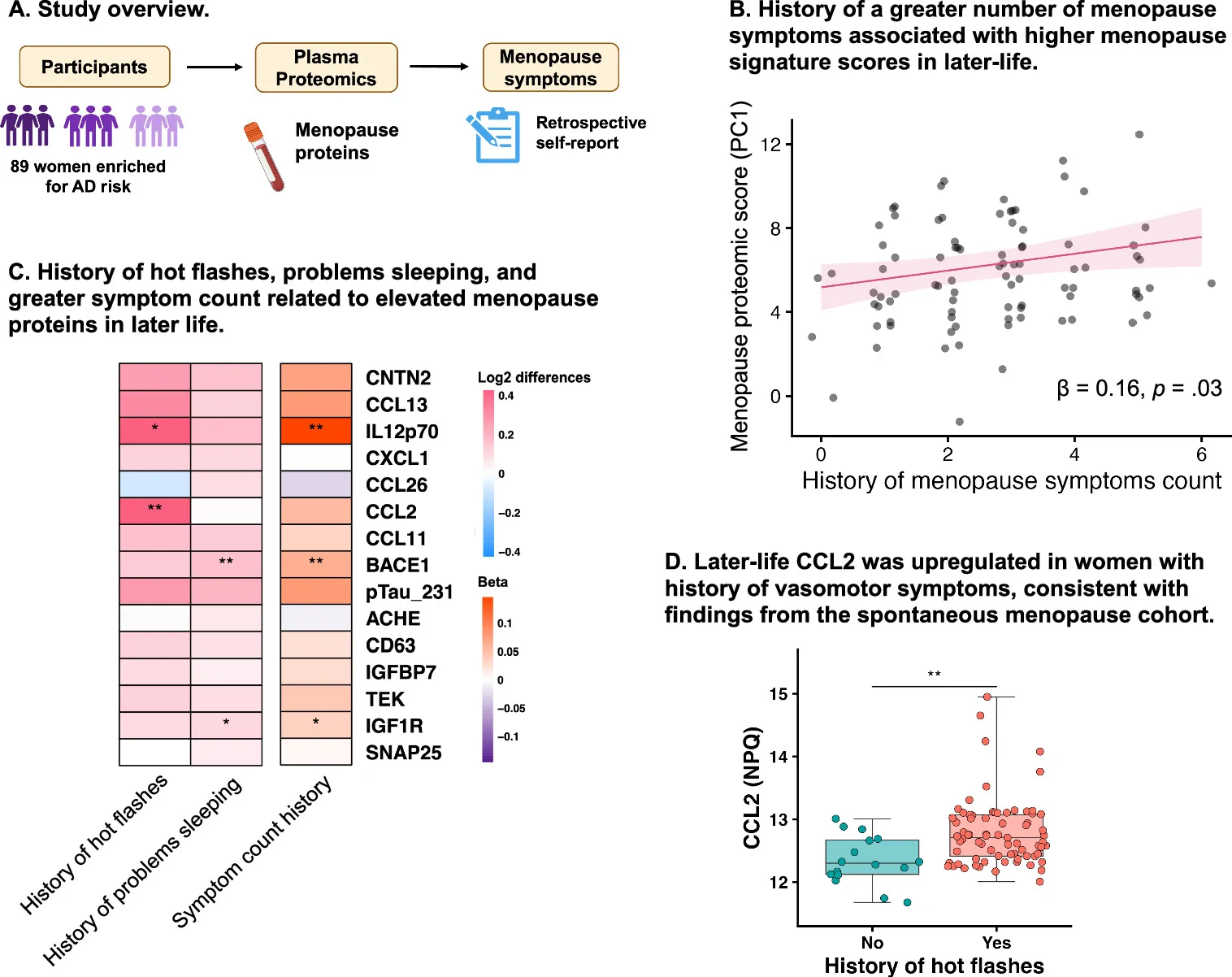
History of menopause symptoms relates to menopause protein levels years to decades later. **A**. In the aging cohort with menopause symptom data (WRAP), women self-reported their history of menopause symptoms, including hot flashes, night sweats, sexual dysfunction, problems sleeping, mood swings, and depression. Analyses evaluated associations of menopause symptom history with menopause scores and individual proteins, adjusted for age, history of hormone therapy, and bilateral oophorectomy. **B**. In a linear regression model, greater total menopause symptom count was associated with higher menopause proteomic scores (nominal *p* = 0.03 from a two-sided significance test), adjusted for age, history of hormone therapy, and bilateral oophorectomy. **C**. Among symptoms that significantly associated with menopause proteomic scores (that is, hot flashes, problems sleeping, and total symptom count), post hoc analyses tested associations with individual menopause proteins, adjusted for age, history of hormone therapy, and bilateral oophorectomy. History of hot flashes associated with increased IL12p70 and CCL2, problems sleeping with increased BACE1 and IGF1R, and symptom count with increased IL12p70, BACE1, and IGF1R. P-values are nominal for these post hoc analyses and derived from two-sided significance tests. Exact statistics are presented in Supplementary Table 10. **D**. A boxplot visualization of CCL2 level by history of hot flashes showed higher levels in women with hot flashes vs. without (*p* = 0.001 from a two-sided t-test). ^*^p < 0.05, ^**^p < 0.01, ^***^p < 0.001. Created in BioRender; Wood, M. https://biorender.com/9h3qdrj (2026).

In 89 women (mean (s.d.) age = 69.2 years (6.84)) in the Wisconsin Registry for Alzheimer’s Prevention (WRAP; Supplementary Table 9), we examined whether history of menopause symptoms showed associations with the identified menopause proteins years to decades after menopause (Extended Data Fig. 4a). Higher menopause proteomic scores were associated with hot flashes (*β* = 0.58, 95% CI = 0.06, 1.11, *P* = 0.03), problems sleeping (*β* = 0.45, 95% CI = 0.03, 0.86, *P* = 0.04) and total symptom count (*β* = 0.16, 95% CI = 0.02, 0.30, *P* = 0.03; Extended Data Fig. 4b). Associations between other menopause symptoms and the proteomic score were not significant (mood swings: *β* = 0.42, 95% CI = −0.01, 0.85, *P* = 0.06; night sweats: *β* = 0.04, 95% CI = −0.37, 0.46, *P* = 0.84; depression: *β* = 0.18, 95% CI = −0.35, 0.71, *P* = 0.50; sexual dysfunction: *β* = 0.41, 95% CI = −0.23, 1.05, *P* = 0.21). Post hoc analyses showed that hot flashes associated with increases in IL-12p70 and CCL2; problems sleeping with increases in BACE1 and IGF1R; and total symptom count with increases in IL-12p70, BACE1 and IGF1R (Extended Data Fig. 4c and Supplementary Table 10). CCL2 was the only individual protein associated with vasomotor symptoms in both older women and midlife women, suggesting a link between vasomotor symptoms and CCL2 that may persist into later life (Extended Data Fig. 4d).

#### Menopause proteomic changes related to cognitive aging and dementia risk

**Fig. 5:**
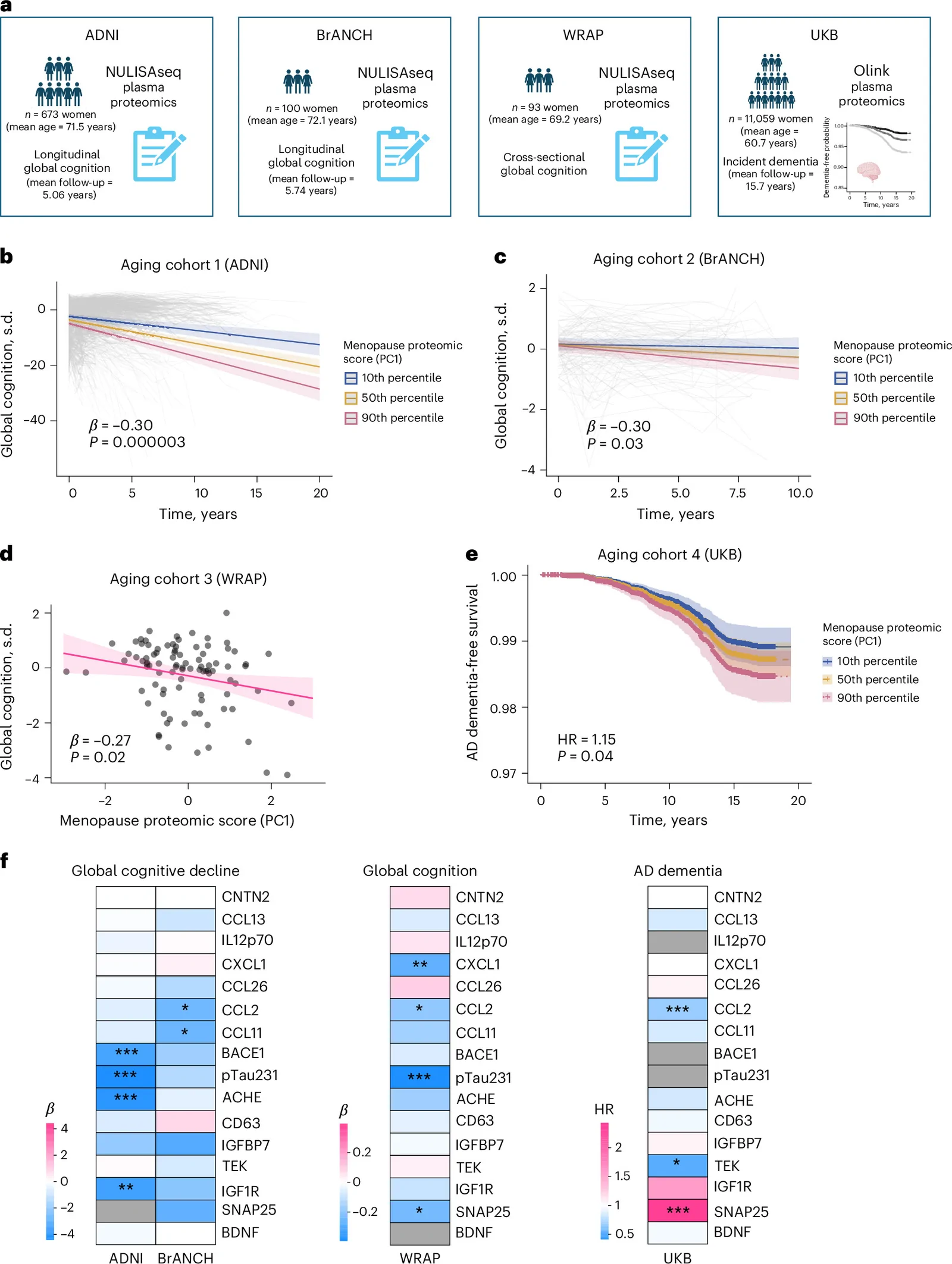
In later life, higher menopause proteomic scores consistently associated with cognitive aging and dementia risk. **a**, We tested associations of the menopause proteomic score (PC1) with cognitive outcomes across four aging cohorts (ADNI; (aging cohort 1), BrANCH (aging cohort 2), WRAP (aging cohort 3) and UKB (aging cohort 4)). **b**–**e**, In all cohorts, higher (more postmenopausal) menopause proteomic scores were associated with cognitive aging and dementia risk, including less favorable cognitive changes in ADNI (**b**) and BrANCH (**c**) in age-adjusted linear mixed models, lower global cognitive scores in WRAP in an age-adjusted linear model (**d**) and greater risk for incident AD dementia in the UKB in an age-adjusted Cox model (**e**). For **b**–**e**, *P* values are nominal results of two-sided significance tests; shaded regions: 95% CI. **f**, In post hoc analyses, individual menopause protein associations with cognitive outcomes varied across cohorts. Panel **a** created in BioRender; Wood, M. https://biorender.com/f5wk8tf (2026). Source data

To test the relevance of proteomic shifts during menopause for later-life cognitive health, we computed the menopause proteomic score in four independent cohorts of older women (Supplementary Table 9) with mean baseline ages ranging from 60.7 to 72.1 years (Fig. 5a). In longitudinal data with an average 5-year follow-up from the AD Neuroimaging Initiative (ADNI; *n* = 673; 54.3% mild cognitive impairment (MCI) or dementia; 49.0% amyloid-positive) and UCSF Brain Aging Network for Cognitive Health (BrANCH; *n* = 100; 1% MCI; 21.7% amyloid-positive), higher (more postmenopausal) baseline menopause proteomic scores associated with a steeper rate of cognitive changes over time (ADNI: *β* = −0.30, 95% CI = −0.42, −0.18, *P* = 0.000003; BrANCH: *β* = −0.03, 95% CI = −0.05, −0.002, *P* = 0.03; Fig. 5b,c). In cross-sectional data from WRAP (*N* = 93; 15.0% MCI/dementia; 64.5% amyloid-positive), higher menopause proteomic scores associated with lower global cognitive performance (*β* = −0.27, 95% CI = −0.51, −0.04, *P* = 0.02; Fig. 5d). Finally, in the UKB (*n* = 11,059), we examined whether menopause proteomic scores associated with risk for incident dementia over a mean (s.d.) of 15.7 (2.84) years of follow-up. Higher menopause scores associated with greater risk for AD dementia (hazard ratio (HR) = 1.15, 95% CI = 1.01, 1.31, *P* = 0.04, *n* = 219 events; Fig. 5e), but not frontotemporal (HR = 0.90, 95% CI = 0.59, 1.36, *P* = 0.61, *n* = 23 events), vascular (HR = 1.15, 95% CI = 0.91, 1.47, *P* = 0.25, *n* = 65 events) or all-cause dementia (HR = 1.08, 95% CI = 0.98, 1.19, *P* = 0.13, *n* = 387 events). Post hoc analyses showed that the individual menopause proteins associations with cognitive outcomes varied across cohorts, suggesting a broader coordinated biological relationship (versus specific individual proteins) (Fig. 5f and Supplementary Tables 1 and 12).

### Sensitivity analyses

**Extended Data Fig. 5:**
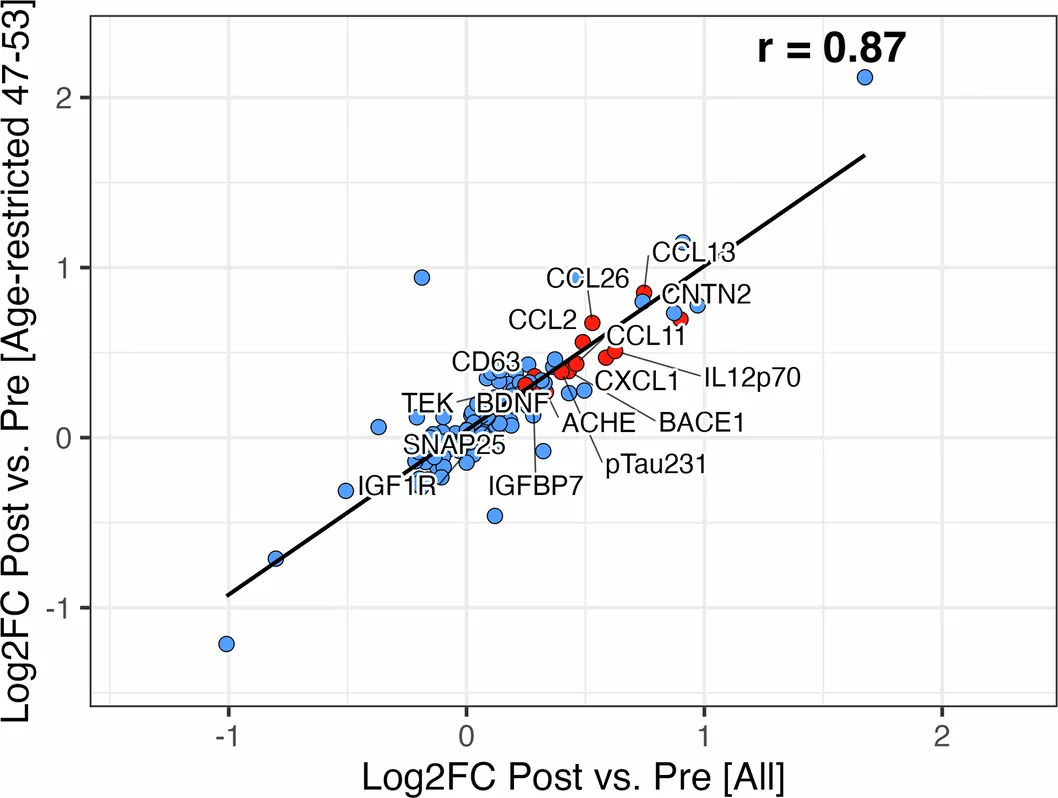
Comparison of log_2_fold change in protein levels in postmenopausal vs. premenopausal women, in the whole UCSB analytic sample and an age-restricted subset (age 47-53 years). To further address the possibility that menopause proteomic differences are driven by chronological (vs. endocrine) aging, we re-estimated age-adjusted ANCOVA models in a subsample of UCSB spontaneous menopause cohort participants with a tightly restricted age range (that is, 47–53 years; *N* = 54 total [*N* = 17 pre-, *N* = 22 peri-, *N* = 15 postmenopausal]; relative to *N* = 80 women aged 43–58 in main analytic sample). The scatter plot depicts a comparison of effect sizes in the main analytic sample (x-axis) versus the age-restricted subset (y-axis), showing strong concordance (r = 0.87). The proteins that significantly differed between pre- and postmenopausal women in main analyses (Supplementary Table 1) are highlighted in red and are labeled.

**Extended Data Fig. 6:**
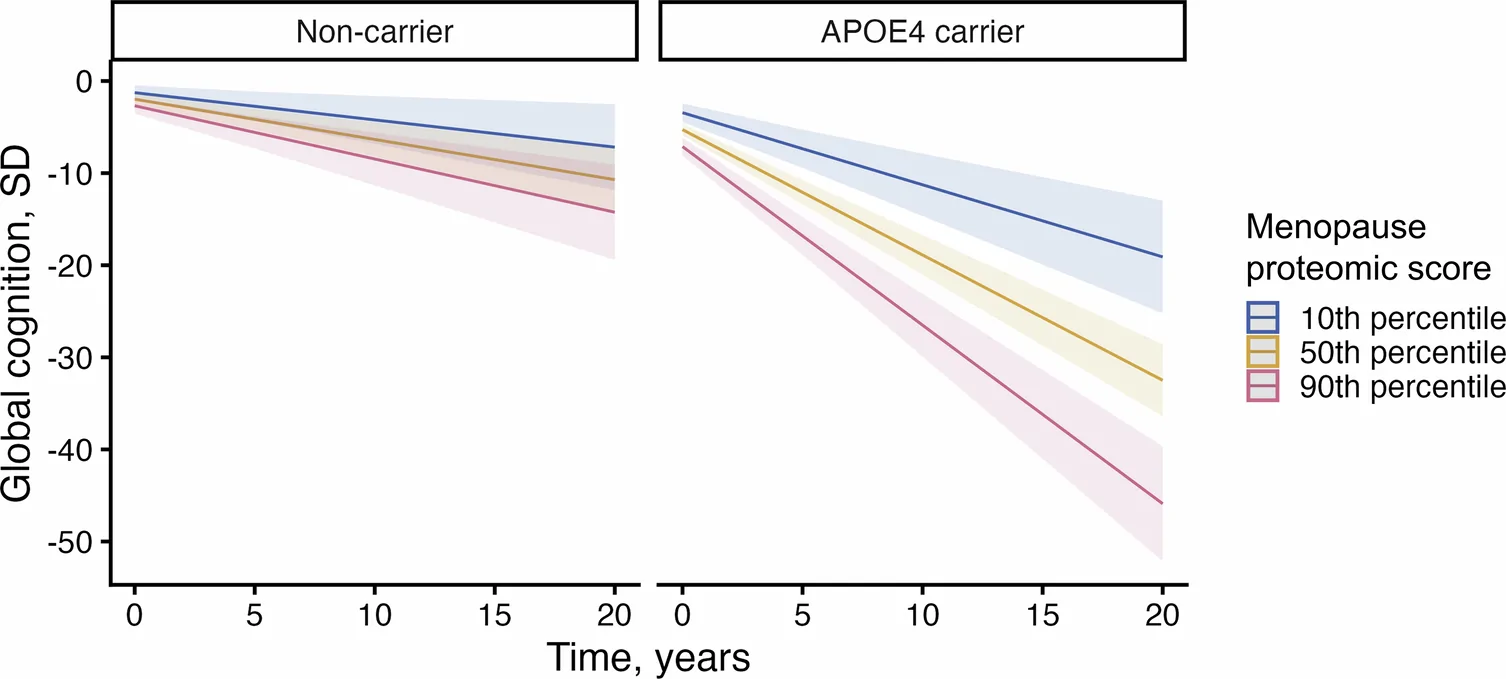
Interactive associations of *APOE4* carriage and menopause proteomic scores on global cognitive decline in ADNI. Exploratory analyses in ADNI (*N* = 673 women) showed that *APOE4* carriage (carrier vs. non-carrier) exacerbated the association of menopause proteomic scores with global cognitive changes (β = −0.39, 95% CI = −0.63, −0.15, *p* = 0.002 for the three-way interaction between menopause score, *APOE4* and time). The colored lines represent the model-estimated predicted values from the linear mixed-effects model, adjusted for baseline age and years of education, and the shaded regions represent 95% confidence intervals. Source data

In sensitivity analyses, our findings were robust to (1) further restriction of the UCSB Healthy Aging Study (HAS) sample by chronological age (47–53 years; *n* = 54; Extended Data Fig. 5 and Supplementary Table 13); (2) adjustment for cardiometabolic comorbidities and in aging cohorts only; (3) exclusion of participants with bilateral oophorectomy; (4) stratification by history of hormone therapy; (5) exclusion of p-tau231 from the menopause proteomic score and (6) exclusion of participants with MCI or dementia at baseline (Supplementary Tables 14 and 15). Exploratory analyses testing effect modification by *APOE4* status showed largely null findings, with the exception that *APOE4* carriage significantly exacerbated the association of the menopause proteomic score with cognitive changes in ADNI (Extended Data Fig. 6 and Supplementary Tables 14 and 15).

## Discussion

Applying attamolar-sensitivity, multiplex proteomics of brain aging in a rigorously staged cohort of midlife women, we identified molecular signals of menopause enriched for inflammatory, metabolic, synaptic and AD biology. Menopause-related proteomic shifts tracked more strongly with hormones than with age. Across perimenopausal, early postmenopausal and older women, vasomotor symptoms related to exaggerated increases in proinflammatory proteins. Top menopause proteomic shifts robustly replicated across analytic platforms in a large, independent cohort of age-matched pre-/peri- and postmenopausal women. These data also revealed wider menopause-associated upregulation of molecular pathways reflecting accelerated multisystem biological aging, notably including brain and oligodendrocyte aging. Further, in four independent cohorts of older postmenopausal women, menopause proteomics associated consistently with less favorable cognitive outcomes, suggesting that menopause-related biological shifts may remain relevant years to decades postmenopause. Collectively, our findings highlight potential biology linking menopause to risk and resilience in cognitive aging. Of note, although all midlife women with an ovary undergo menopause, most do not develop dementia; instead, the menopause transition may represent a vulnerable window for understanding and identifying the earliest signals of future brain aging risk.

The predominance of immune and inflammatory molecules associated with the menopause transition (for example, CCL2, CCL11, TEK) alongside upregulation of pathways related to cytokine signaling and the complement cascade align with observed rises in systemic inflammation postmenopause^2,23^. We also identify menopause-associated shifts in synaptic markers (ACHE, SNAP25, CNTN2), and demonstrate links between menopause and blood markers of AD biology (p-tau231, BACE). These findings are consistent with animal models demonstrating widespread disruptions in synaptic plasticity, spine density and cholinergic signaling^4,26,27^ and extend recent studies showing elevations in Aβ and tau-positron emission tomography (PET) across menopause^28–30^. The clinical relevance of subthreshold elevations in p-tau231 and BACE1 in midlife is unclear. Overall, our findings align with previous observations of accelerated biological aging across menopause^31^ and underscore specific links to brain-relevant targets, overall brain aging and oligodendrocyte age.

Menopause proteomic differences tracked more strongly with hormones than age, suggesting that the observed biological changes may be driven primarily by ovarian versus chronological aging. Many proteins were most linked strongly to FSH, including key targets implicated in AD pathobiology and synaptic processes (that is, BACE1, ACHE, CNTN2)^32–34^. FSH receptors are expressed abundantly in AD-vulnerable brain regions, and preclinical models show that FSH can trigger the development of AD pathology, neuroinflammation and cognitive deficits^35^. By contrast, lower estradiol was linked to increases in proinflammatory markers (CCL26, CCL2, TEK), aligning with the established role of estrogens in modulating immune responses^36^.

Vasomotor symptoms were associated with menopause proteomic scores, across both midlife and older women with histories of vasomotor symptoms. These associations were driven primarily by proinflammatory targets, with CCL2 (that is, MCP-1) the most replicable. Vasomotor symptom severity has been associated previously with vascular inflammation^37,38^ (including CCL2^39^), cardiovascular risk and AD brain changes^20,21^. Vasomotor symptoms may signal less adaptive physiological responses to menopause that confer risk for adverse brain aging and other chronic diseases. It is unclear why only night sweats (not hot flashes) were associated with the menopause score in midlife women, and only hot flashes (not night sweats) were associated with the menopause score in the later-life cohort. Given their shared etiology^10^, this inconsistency may relate to recall bias and imprecision introduced by self-report measures. Although vasomotor symptoms originate in the hypothalamus, these symptoms are multifactorial and can be impacted by both neurologic and nonbrain physiologic and contextual factors^10,11^. There were also unexpected associations of vaginal dryness with lower menopause scores, the mechanistic significance of which is unclear.

Our study provides evidence that menopause-related proteomic shifts are detectable and linked to cognitive changes in later life. Across four independent older adult cohorts, menopause proteomic scores associated with less favorable cognitive outcomes, including cognitive changes and greater risk of AD dementia. The effect size for AD dementia was relatively small, and the associations of individual proteins with cognitive outcomes varied across cohorts. These findings may reflect differences in cohort composition (for example, age, cognitive status, amyloid positivity), as well as proteomic platform differences (NULISAseq/Olink). However, these results could also suggest that associations between menopause proteomic changes and cognitive outcomes are driven less by specific targets than by coordinated shifts that may reflect pro-aging biology generally^40–42^.

Strengths of this study include the unique midlife cohort with rigorously defined menopause stages to identify proteomic signals of menopause that largely replicated cross-platform in a validation cohort of postmenopausal women age-matched to pre-/peri-menopausal women. The age-matched validation provides compelling evidence that the observed proteomic changes were driven more strongly by endocrine than chronological aging. Three of the targets identified in the UCSB cohort did not show concordant elevations in the UKB sample (that is, BDNF, CD63 and CCL26), which may reflect proteomic platform differences, spurious findings or coarse evaluation of menopause status in the UKB. Of note, although we used a composite score to summarize menopause-associated biological signals observed on ultrasensitive NULISAseq in our deeply phenotyped cohort, the menopause proteomic score does not represent the full breadth of menopause-related biology detectable in circulation, as evidenced by the widespread proteomic shifts observed in the UKB Olink analyses. That is, the NULISAseq central nervous system (CNS) panel captures only ~120 proteins potentially involved in neurologic aging, whereas other high-throughput assays may allow expanded discovery. Furthermore, while serum and plasma seem to perform comparably on the NULISAseq CNS panel^43^, sample matrix differences may influence protein measurements making cross-cohort comparison more challenging. Other limitations include the small spontaneous menopause discovery sample, which included only cross-sectional protein and hormone data, the reliance on self-reported menopause symptoms, and that proteins in blood may reflect multiple tissue sources. Longitudinal studies mapping within-person trajectories of hormones, multiomics, symptoms and cognitive and brain health across menopause will be critical to confirm the observed menopause-associated proteomic changes and disentangle the complex interplay between endocrine, neurologic and chronological aging.

We defined blood-based proteomic signals of menopause, enriched for inflammatory, metabolic, synaptic and AD biology alongside accelerated biological aging. We demonstrated that inflammatory changes are especially pronounced in women with vasomotor symptoms, highlighting CCL2 as a signal of this physiologic susceptibility that may persist into later life. We further illustrated that menopause biology shows continued relevance to cognitive aging across four independent cohorts of older women. These findings shine light on the biological processes connecting menopause to dementia risk, affirming the importance of menopause as a critical window for our foundational understanding of brain aging.

## Methods

The UCSB HAS (spontaneous menopause cohort) was approved by the institutional review board of UCSB (UCSB Human Subjects Committee). The UKB received approval from the North West Multicentre Research Ethics Committee and is overseen by an independent Ethics and Governance Council. WRAP was approved by the University of Wisconsin Institutional Review Board. UCSF BrANCH was approved by UCSF Institutional Review Board. ADNI was approved by local institutional review boards or equivalent ethics committees at each participating site. All participants provided informed consent.

### Spontaneous menopause cohort participants

The UCSB HAS recruited approximately equal numbers of pre-, peri- and postmenopausal women (*n* = 80) plus age-matched men (*n* = 36). Menopause was staged following STRAW+10 criteria^24^. Participants were aged 35–60 years, not currently taking menopausal hormone therapy, and did not have any notable medical conditions. Participants who were not taking hormonal contraceptives were prioritized for recruitment, resulting in a sample that was largely free of exogenous hormones (*N* = 1 on oral contraceptives). Female participants were required to have an intact uterus and no history of bilateral oophorectomy. Female participants self-reported how often they experienced commonly endorsed menopause symptoms^44,45^ including hot flashes, night sweats, vaginal dryness and irritability in the last 2 weeks, on a scale of ‘not at all,’ ‘1–5 days,’ ‘6–8 days,’ ‘9–13 days’ or ‘every day.’ Due to the small sample size, each individual symptom was dichotomized as present versus absent.

### Endocrine assays

In the UCSB HAS, serum was analyzed for gonadotropin and sex steroid hormones: FSH, 17β-estradiol, progesterone, SHBG, DHEAS and testosterone. All participants fasted for 8 h before a morning venous blood draw. Women who were still menstruating were scheduled in the early follicular phase (day 3–5) of their menstrual cycle, pursuant to subject report. Serum was analyzed at the Brigham and Women’s Hospital Research Assay Core. 17β-estradiol, progesterone, and testosterone were determined by liquid chromatography mass-spectrometry, and FSH, SHBG and DHEAS by chemiluminescent immunoassay (Beckman Coulter). Assay sensitivities, dynamic range and intra-assay coefficients of variation (CVs) (respectively) were as follows: estradiol, 1 pg ml^−1^, 1–500 pg ml^−1^, <5% relative standard deviation (RSD); progesterone, 0.05 ng ml^−1^, 0.05–10 ng ml^−1^, 9.33% RSD; testosterone, 1.0 ng dl^−1^, 1–2,000 ng dl^−1^, <4% RSD; FSH, 0.2 mIU ml^−1^, 0.2–200 mIU ml^−1^, 3.1–4.3%; SHBG, 0.33 nmol l^−1^, 0.33 200 nmol l^−1^, 4.5–4.8%; DHEAS, 2 μg dl^−1^, 2–1,000 μg dl^−1^, 1.6–8.3%. All six hormones were measured in women, and the latter three were measured in men. Hormone levels were log-transformed and expressed in s.d. units to aid interpretability.

### Blood proteomics

Serum (menopause cohort) or plasma (aging cohorts) was analyzed on the NULISAseq CNS panel to measure 127 proteins in UCSB HAS, 131 in ADNI, 132 in BrANCH and 122 in WRAP^25,46,47^. The automated Alamar Argo HT workflow generated immunocomplexes tagged with both sample-specific and target-specific DNA barcodes. Samples were pooled into a single library for next-generation DNA sequencing. Samples were randomized (nonstratified) across plates and run in singlet, per previously established procedures for NULISA assays^25,47^. Batch (plate) effects were mitigated using the standard NULISA protein quantification (NPQ) protocols and algorithm (as described previously)^47^, including hierarchical normalization that consists of internal spike-in controls for well-level normalization, pooled interplate controls to harmonize protein measurements across plates and negative controls to estimate background and limits of detection. Protein concentrations were reported in NPQ units, which are computed by normalizing the relative concentration of each analyte using internal and inter-plate controls and log_2_-transforming the normalized values. Plate- and sample-level quality control (QC) metrics (for example, CV, detectability) were used to assess assay performance. The limit of detectability is determined as the mean plus three times the s.d. of the exponentiated (that is, anti-log-transformed) normalized counts for the negative control samples on the plate, which are run in quadruplicate. Detectability is then calculated as the percentage of samples in which the quantification is above the limit of detectability for that target.

In all cohorts, QC metrics were in acceptable ranges, including for both intraplate internal (UCSB HAS: 15.0%; BrANCH: 7.3%; ADNI: 5.9%; WRAP: 0.01–7.5%; all within maximum threshold of 25%) and interplate (UCSB HAS: 4.0%; BrANCH: 8.6%; ADNI: 11.8%; WRAP: 0.29–5.6%; all within maximum threshold of 15%) control CV. In the UCSB HAS, eight proteins (UCHL1, SNCB, PTDP43409, PTN, p-Tau217, OligoSNCA, IL1B and NRGN) had median detectability of <50%, so were removed from analyses following previous work with the NULISA panel^48^. In all other NULISA cohorts (BrANCH, ADNI), our analyses were restricted to the 16 menopause proteins identified in the UCSB HAS. All 16 of these menopause proteins were detectable (≥50%) in BrANCH and WRAP and all except SNAP25 were detectable (≥50%) in ADNI. SNAP25 was removed from analyses in ADNI. Given that APOE4 protein expression proxies *APOE4* genotype (bimodal distribution), we excluded APOE4 from proteomics analyses. In the UCSB HAS, APOE4 levels were used to infer *APOE4* allele carriage (APOE4 ≥ 10 NPQ categorized as carrier, APOE4 < 10 NPQ categorized as noncarrier; Supplementary Fig. 3). A full list of proteins in each cohort is provided in Supplementary Table 16.

To reduce the influence of extreme values, all protein concentrations were winsorized at the three-interquartile limit. There were no missing values on any examined proteins in the UCSB HAS cohort. No imputation was used for NULISAseq data, and analyses in other NULISA cohorts (BrANCH, ADNI, WRAP) were restricted to participants with nonmissing values on all menopause proteins. Given all analyses examined proteomic outcomes within cohorts separately, we did not normalize protein levels between cohorts.

### Cross-cohort, cross-platform validation in UKB

To evaluate whether spontaneous menopause proteomic shifts were robust to cohort and proteomic platform, we compared spontaneous menopause protein effect sizes detected in the midlife menopause cohort with NULISAseq proteomics to menopause protein effect sizes detected in a large cohort of 2,814 women from the UKB with cross-sectional proximity extension assay Olink proteomics^49^. The UKB is a population-based prospective cohort study with multiomics and clinical data on approximately 500,000 participants aged 40–69 years. The UKB Pharma Proteomics Project (UKB–PPP) consortium generated proximity extension assay Olink plasma proteomics data on a subset of participants’ baseline samples, resulting in up to 2,923 protein measurements from 52,995 participants in the post-UKB–PPP quality control dataset. Details of proteomics data generation, processing and QC have been described previously^49^ and are available at https://biobank.ndph.ox.ac.uk/ukb/ukb/docs/PPP_Phase_1_QC_dataset_companion_doc.pdf and https://biobank.ndph.ox.ac.uk/crystal/ukb/docs/Olink-3072_B0-B6_Analysis-Report.pdf. In brief, samples were aliquoted using a pseudorandomization algorithm to maximize variation of phenotypes on each generated sample plate, balancing factors such as age, sex, day of the week of blood collection, self-reported ethnicity and deprivation index^49^. QC metrics were all in acceptable ranges for Olink proteomics, including median intraplate CVs <8%, median interplate CVs <11%, median interinstrument CVs <11% and 73.2% of proteins had <30% of measurements falling below the limits of detectability^49^. Normalized protein expression values were calculated per standard, previously described algorithms that include normalizing for both plate-to-plate and batch-to-batch variation. To maintain consistency with other published UKB proteomics papers^49,50^, we used the standard, postquality control UKB Olink dataset consisting of 2,923 unique proteins, including 13 of the menopause proteins identified in UCSB (p-tau231, BACE1 and IL-12p70 were not measured in UKB).

A total of *n* = 17,487 female participants reported being postmenopausal at baseline (answered ‘yes’ to ‘Have you had your menopause (periods stopped)?’), and *n* = 6,542 female participants reported not being postmenopausal at baseline (answered ‘no’; *n* = 3,272 with hysterectomy and *n* = 1,231 not sure/prefer not to answer excluded). Of the resultant sample of *n* = 24,029 pre-/peri- and postmenopausal women, we further restricted our menopause analyses to participants who reported no history of bilateral oophorectomy (*n* = 22,809; *n* = 1,220 excluded), no current use of menopausal hormone therapy (*n* = 21,910; *n* = 899 excluded) and who were aged 45–60 years at the time of the Olink blood draw to mirror the UCSB HAS sample more closely (*n* = 10,926 (*N* = 3,789 pre-/peri-menopausal, *n* = 7,137 postmenopausal); *n* = 10,984 excluded).

To mitigate the confound between age and menopause status, we created age-matched samples of pre-/peri- and postmenopausal women (R ‘MatchIt’ package). Pre-/peri- and postmenopausal women were matched on a 1:1 ratio using *k-*nearest-neighbor (KNN) matching without replacement and a 0.05 s.d. caliper. This resulted in a sample of *n* = 1,407 pre-/peri-menopausal women and *n* = 1,407 postmenopausal women who were nearly matched exactly on age (Fig. 4a and Supplementary Table 6) for validation analyses.

### Aging cohorts

To evaluate the relevance of menopause-related biological changes to later-life cognitive outcomes, we used proteomics data from women in four independent aging cohorts.

#### ADNI (aging cohort 1)

Data used in the preparation of this article were obtained from the Alzheimer’s Disease Neuroimaging Initiative (ADNI) database (adni.loni.usc.edu). The ADNI was launched in 2003 as a public-private partnership, led by Principal Investigator Michael W. Weiner, MD. The primary goal of ADNI has been to test whether serial magnetic resonance imaging (MRI), positron emission tomography (PET), other biological markers, and clinical and neuropsychological assessment can be combined to measure the progression of mild cognitive impairment (MCI) and early Alzheimer’s disease (AD). ADNI is an international multisite observational study that follows older adults across the cognitive spectrum (that is, cognitively normal, MCI and AD)^51^. No data were collected on menopause history or symptoms. Participants underwent repeated cognitive assessments and blood collection every 6–12 months. To quantify global cognition, we examined the ADNI modified Preclinical Alzheimer’s Cognitive Composite score, which was computed by averaging sample-based *z*-scores on the Alzheimer Disease Assessment Scale—Cognitive Subscale Delayed Word Recall, Logical Memory Delayed Recall, the Mini Mental State Examination and (log-transformed) Trail-Making Test B Time to Completion^52^. For a subset of ADNI participants (*n* = 1,248), baseline plasma was analyzed for 131 proteins on the NULISAseq CNS platform. Our analytic sample included *n* = 673 female participants who had NULISAseq data and cognitive test data available. To characterize the sample, amyloid positivity was determined using the derived ADNI variable ‘AMYSTAT,’ which uses validated, tracer-specific quantitative thresholds to classify participants’ amyloid status as ‘elevated’ or ‘not elevated’ from harmonized Aβ PET data^53,54^.

#### UCSF BrANCH (aging cohort 2)

BrANCH is a study of community-dwelling, functionally intact participants who undergo annual neuropsychological testing and blood draws. Female participants self-reported their histories of hormone therapy use and oophorectomy. A composite score of global cognition was computed by averaging sample-based *z*-scores for tests of memory (California Verbal Learning Test II immediate and delayed recall), executive function (Stroop interference, modified Trail-Making Test, phonemic fluency and Digit Span Backwards), and processing speed (six computerized visually based tests of Length Judgment, Visual Search, Distance Judgment, Abstract Matching 1, Abstract Matching 2 and Shape Judgment), described previously^55^. Baseline plasma was analyzed for 132 proteins on the NULISAseq CNS platform. Our analytic sample included *n* = 100 female participants with NULISAseq who had cognitive data available. Baseline plasma p-tau181/Aβ42 was assayed on Simoa (Quanterix) across three batches in the broader BrANCH cohort (1,266 samples across *n* = 664 participants). Batch effects were quantified by linear regression on samples shared across batches, with model estimates applied subsequently to all samples to align cross-batch values on the same scale. To characterize the sample, amyloid positivity was determined using a sample-specific, batch-harmonized p-tau181/Aβ42 threshold that optimized classification of visually read Aβ-PET 18F-AV45 positivity in the broader BrANCH cohort (area under the receiver operating characteristic curve = 0.88).

#### WRAP (aging cohort 3)

WRAP is an observational study enriched for participants with a parental history of AD. Participants completed clinical and cognitive testing and blood collection approximately every 2 years. Female participants self-reported their histories of hormone therapy use, oophorectomy and menopause symptoms. Menopause symptoms reported included hot flashes, night sweats, sexual dysfunction, problems sleeping, mood swings and depression, which together are among the most common and distressing menopause symptoms observed in midlife women^56–61^, plus total symptom count. A composite score of global cognition was calculated by averaging sample-based *z*-scores on tests for memory (Rey Auditory Verbal Learning Test total learning and delayed recall, Logical Memory IA and IIA) and executive function (Trail-Making Test Part B, Weschler Adult Intelligence Scale–Digit Symbol Substitution Test and Animal fluency). Plasma samples from a subset of recent WRAP visits (2017–2024) were analyzed for 122 proteins on the NULISAseq CNS kit^46^. Given the lack of sufficient follow-up after the NULISAseq blood draw, we restricted our analyses to cross-sectional data. Our sample included *n* = 93 female participants who had cognitive data available within 30 days of NULISA blood collection. Plasma p-tau217/Aβ42 was measured on the Lumipulse G System (Fujirebio) at the Michael T. Zuendel Biomarker Laboratory at Banner Sun Health Research Institute. To characterize the sample, amyloid positivity was determined using a threshold of >0.008 (ref. ^62^).

#### UKB (aging cohort 4)

To assess whether menopause-related proteomic shifts predict risk for dementia, we used data from *N* = 11,059 women ≥50 years old in the UKB with nonmissing values for all 13 menopause proteins measured on Olink at study baseline. Participants were free from known dementia at baseline and followed for a mean (s.d.) of 15.7 (2.84) years. Consistent with previous work, incident dementia cases were defined algorithmically using a combination of information in International Classification of Diseases diagnosis codes in linked primary care, hospital inpatient and death registry data, for all-cause dementia (codes A81, F00 to F03, F05, F10, G30, G31 and I67), alongside AD (F00 and G30), vascular dementia (F01 and I67) and frontotemporal dementia (F02 and G31)^50^. Participants were censored at first dementia diagnosis, death or September 2025, representing the most recent update of algorithmically defined dementia outcomes in the UKB.

### Analyses

Analyses were conducted in R (v4.5.0) and Python (v3.11.14). Descriptive statistics and analysis of variance (ANOVA), *t*-tests and *χ*^2^ tests summarized demographic and clinical characteristics in each cohort.

### Spontaneous menopause cohort

Age-adjusted analysis of covariance (ANCOVA) compared levels of 126 NULISAseq proteins by menopause stage (pre, peri or post). Menopause proteins were identified as those that differed between pre- and postmenopausal women per nominal statistical significance (unadjusted *P* < 0.05). All further analyses focused exclusively on these proteins. We performed a PCA on the identified menopause proteins in all midlife women and extracted the first component (PC1) as a ‘menopause proteomic score.’ To limit comparisons, the menopause score was used as the primary measure for all further analyses, with effects of individual proteins examined in post hoc analyses. Separate age-adjusted regressions tested the associations of menopause stage and sex with the menopause score.

Although menopause definitionally involves endocrine shifts, different hormones may relate to unique aspects of menopause biology. To probe this, age-adjusted regressions tested associations between sex hormones and the menopause proteomic score in all midlife women. Among peri- and postmenopausal women, separate regressions tested associations of menopause symptoms with the menopause score, adjusting for age and menopause stage.

### Cross-cohort, cross-platform validation

In the age-matched sample of pre-/peri- and postmenopausal women in the UKB, age-adjusted regression models tested associations between menopause status (post versus pre/peri) and 2,923 Olink proteins, with FDR correction. For the menopause proteins identified in UCSB (NULISAseq) that were measured on Olink, we examined whether these proteins also differed between pre-/peri- and postmenopausal women in the UKB, including estimated proteomic effect sizes between cohorts. We performed post hoc GO and pathway enrichment using the publicly available GO parallel code (https://www.github.com/edammer/GOparallel)^63^.

We also tested associations of menopause stage with proteomic clocks of organ and cellular aging^50,64^. Using publicly available code for established Olink models, we calculated proteomic aging clocks for 13 organ systems (github.com/hamiltonoh/organageUKB) and 38 cell types (github.com/dingdaisy/cellage)^50,64^. Organ and cell clock models were developed using proteins that are labeled as enriched for the corresponding tissue or cellular sources in the Human Protein Atlas^65^, such that proteins that load onto each organ- or cell-specific model are assumed to originate at least in part from those organs and/or cells^50^. Because the organ and cell clock estimations required complete data, these analyses were performed on a subset of *n* = 2,340 age-matched pre-/peri- and postmenopausal women following KNN imputation of the Olink data^50^.

We imputed Olink proteomics data using the entire UKB Olink baseline sample (*n* = 52,995 participants; *n* = 2,923 proteins) following a previously published method^50^. For quality control, we first excluded *n* = 8,181 samples with more than 1,000 proteins missing, 7 proteins with missing values in more than 10% of samples, and 338 samples with discordant self-reported sex and genetic sex, resulting in a final dataset of *n* = 44,476 participants with 2,916 protein measurements. We performed missing value imputation of the Olink data using KNN imputation. We split the data into train and test, where each split comprised 11 randomly selected UKB assessment centers (train centers: Newcastle, Nottingham, Bristol, Leeds, Sheffield, Glasgow, Wrexham, Croydon, Hounslow, Edinburgh, Reading; test centers: Barts, Birmingham, Bury, Cardiff, Liverpool, Manchester, Middlesborough, Oxford, Stockport, Stoke, Swansea). Protein levels were *z*-transformed using the mean and s.d. values of the train split. Using scikit-learn’s KNNImputer function (scikit-learn.org), we trained a KNN imputer where the number of neighbors (*k*) was set to the square root of the sample size in the train split (*k* = 166). We assessed the imputer’s performance on *n* = 5,590 samples (3,425 train; 2,165 test) with no original missing protein values. To do so, we input missing values randomly into this ‘ground truth’ subsample at 3% (equivalent to the missing value rate in the entire post-QC dataset). We then used the imputer on this subsample to calculate the error between imputed values and ground truth values. This resulted in a total mean error rate of 0.57 in both the train and test samples, consistent with previous research^50^ and supporting robust imputation of the Olink data (Supplementary Fig. 4).

Chronological age-adjusted linear models tested associations of age at menopause with organ and cell age ‘gaps’ (that is, *z*-scored residuals of proteomic predicted age linearly regressed against actual age, whereby larger gaps reflect more advanced biological aging).

### Aging cohorts

We extracted loadings and summary statistics for the menopause score (PC1) calculated in the spontaneous menopause cohort to compute an equivalent score in each aging cohort. To accommodate varying protein availability between aging cohorts (Supplementary Table 16), we recomputed several versions of PC1 in the spontaneous menopause cohort after (1) removing SNAP25 for use in ADNI; (2) removing BDNF for use in WRAP and (3) removing BACE1, IL-12p70 and p-tau231 for use in UKB. Loadings for the remaining shared proteins among the versions were very similar (Supplementary Fig. 5).

In WRAP, linear regressions tested associations of menopause symptom history with the menopause score, adjusting for age, history of hormone therapy and bilateral oophorectomy.

To examine the relevance of the menopause score to cognitive aging outcomes, in ADNI and BrANCH, linear mixed-effects models tested associations of the interaction between the score and time on global cognition, adjusting for baseline age and education. In WRAP, a linear regression tested the association of menopause score on global cognition, adjusting for age and education. In the UKB, Cox proportional-hazards models tested associations of the menopause score with risk for incident dementia (AD, frontotemporal, vascular, all-cause), adjusting for age.

### Sensitivity analyses

To further address the possibility that menopause proteomic differences are driven by chronological (versus endocrine) aging, we performed sensitivity analyses repeating models in a subsample of participants with a tightly restricted age range (47–53 years; *N* = 54 total; *n* = 17 pre-, *n* = 22 peri-, *n* = 15 postmenopausal). These analyses yielded very similar findings to the main results (Extended Data Fig. 5 and Supplementary Table 14), suggesting the observed proteomic differences between menopause groups were not confounded substantially by chronological age. In the UKB age-matched validation cohort, we re-ran differential abundance analyses, also adjusting for factors that systematically differed between pre- and postmenopausal women (years of education, history of hormone therapy use, current hormonal contraceptive use, ethnicity, smoking status, low-density lipoprotein (LDL) cholesterol and glycated hemoglobin (hbA1c)), which yielded highly consistent findings to the main analyses (Supplementary Fig. 6). In all cohorts, we also re-estimated models after adjusting for cardiometabolic comorbidities and common health-related risk factors that may influence the proteome, per available data in each cohort. These covariates included: UCSB HAS: BMI, smoking history and self-reported histories of high blood pressure, high cholesterol and diabetes; ADNI: BMI, smoking history, systolic blood pressure and diastolic blood pressure; BrANCH: BMI, smoking history, systolic blood pressure, diastolic blood pressure, hbA1c and LDL cholesterol; WRAP: BMI, smoking history, systolic blood pressure, diastolic blood pressure, fasting glucose and LDL cholesterol; UKB: BMI, smoking history, systolic blood pressure, diastolic blood pressure, hbA1c and LDL cholesterol. All analyses yielded similar results to main models (Supplementary Tables 14 and 15).

In aging cohorts, we performed additional sensitivity analyses re-estimating models after excluding participants who self-reported a history of bilateral oophorectomy (not available in ADNI) and stratified by history of hormone therapy (ever used versus never used; also not available in ADNI), which also yielded similar findings (Supplementary Tables 9 and 15). Furthermore, given the known associations of plasma p-tau with cognitive decline and AD risk, we computed an alternate version of the menopause signature excluding p-tau231 (Supplementary Fig. 5). Analyses excluding p-tau231 yielded a very similar pattern of results to the main analyses (Supplementary Table 15). In ADNI, BrANCH and WRAP, which had detailed information on baseline cognitive diagnoses, we also re-estimated models after excluding participants with MCI or dementia at baseline, which again produced similar findings (Supplementary Tables 9 and 15).

In both the UCSB HAS and aging cohorts, we finally performed exploratory analyses testing effect modification by *APOE4* genotype (that is, carrier versus noncarrier). *APOE4* carriage was inferred using NULISAseq serum levels of the APOE4 protein in the UCSB HAS cohort (Supplementary Fig. 5) and was determined using genetic data for the single-nucleotide polymorphisms rs429358 and rs7412 in the aging cohorts (ADNI, BrANCH, WRAP and UKB). These showed largely null effects, with the exception that *APOE4* carriage significantly exacerbated associations of menopause proteomic scores with cognitive changes in ADNI (Supplementary Tables 14 and 15 and Extended Data Fig. 6).

### Reporting summary

Further information on research design is available in the Nature Portfolio Reporting Summary linked to this article.

## Online content

Any methods, additional references, Nature Portfolio reporting summaries, source data, extended data, supplementary information, acknowledgements, peer review information; details of author contributions and competing interests; and statements of data and code availability are available at https://doi.org/10.1038/s41591-026-04648-4.

## Supplementary information

Supplementary Information Supplementary Figs. 1–6.

Reporting Summary

Peer Review File

Supplementary Tables 1–16 Supplementary Tables 1–16.

## Source data

Source Data Fig. 1 Figure 1 source data. **b**, Source data cannot currently be shared because participant-level data is protected as outlined in ‘Data Availability.’ **c**, Source data in Supplementary Table 1. **d**, Source data for PC1 plot in sourcedata_Fig1D.xlsx; participant-level data for boxplot cannot be shared as noted above. **e**, Source data in Supplementary Tables 1–3. **f**, Participant-level data for boxplot cannot be shared as noted above; source statistics in Supplementary Tables 1 and 3.

Source Data Fig. 2 Figure 2 source data. **b**, Source data cannot be shared because participant-level data is protected as outlined in ‘Data availability’; statistics in Table 1. **c**, Source data for scatterplots cannot be shared because participant-level data is protected as noted above. **d**, Source data in Supplementary Table 4. **e**, Source data in Supplementary Table 4. **f**, Source data in sourcedata_Fig2F.xlsx.

Source Data Fig. 4 Figure 4 source data. **b**,**d**, Source data cannot be shared because participant-level data is protected as outlined in ‘Data Availability.’ **c**, Source data in Supplementary Table 7. **e**, Source data in Supplementary Tables 1 and 7. **f**, Source data in sourcedata_Fig4F. **g**,**h**, Source data in Supplementary Table 8.

Source Data Fig. 5 Figure 5 source data. **b**,**d**, Source data in data_Fig5B-D.xlsx for model-plotted estimates; data for spaghetti points cannot be shared because participant-level data is protected as outlined in ‘Data Availability.’ **e**, Source data for scatterplot cannot be shared as participant-level data is protected. **f**, Source data in Supplementary Tables 11 and 12.

Source Data Extended Data Fig. 6 Source data for model-plotted estimates in sourcedata_ExtFig6.

## Data Availability

Data from the UCSB Healthy Aging Study (spontaneous menopause cohort) are currently being prepared for inclusion in the Global Neurodegeneration Proteomics Consortium and are expected to be available to qualified researchers through the standard Global Neurodegeneration Proteomics Consortium data access protocol in early 2027 (https://www.neuroproteome.org/harmonized-data-set-hds). Questions can be directed to E. Jacobs and the Bowers WBHI team at info@wbhi.ucsb.edu. Data access requests for the other included studies can be submitted at the following links: UKB, https://ukbiobank.ac.uk/use-our-data/apply-for-access; WRAP, https://wrap.wisc.edu/data-requests-2; UCSF BrANCH, https://memory.ucsf.edu/research-trials/professional/open-science#data-sharing; ADNI, https://adni.loni.usc.edu/data-samples/adni-data. Access to controlled datasets requires submission of a formal application and adherence to the appropriate data use agreements. Requests are reviewed and responded to by the relevant data access committees according to each study’s data access procedures. Source data are provided with this paper.

## Code availability

Algorithms used for data analyses are accessible in publicly available, existing R and Python packages and code repositories. The code used to perform GO/pathway analysis is available at https://github.com/edammer/GOparallel. The code used to generate proteomic clocks of organ aging is available at https://github.com/hamiltonoh/organageUKB. The code used to generate proteomic clocks of cell aging is available at https://github.com/dingdaisy/cellage.
