# Supplementary material for: Blood proteomics of menopause map to brain aging and dementia risk
Source: Nat Med. 2026 Sep 22;32(11):4244–55. doi: 10.1038/s41591-026-04648-4 (PMC13768193; doi:10.1038/s41591-026-04648-4)

nature medicine

Supplementary information https://doi.org/10.1038/s41591-026-04648-4

Blood proteomics of menopause map to brain aging and dementia risk

In the format provided by the authors and unedited

Figure S1. Variance plot for principal components analysis.

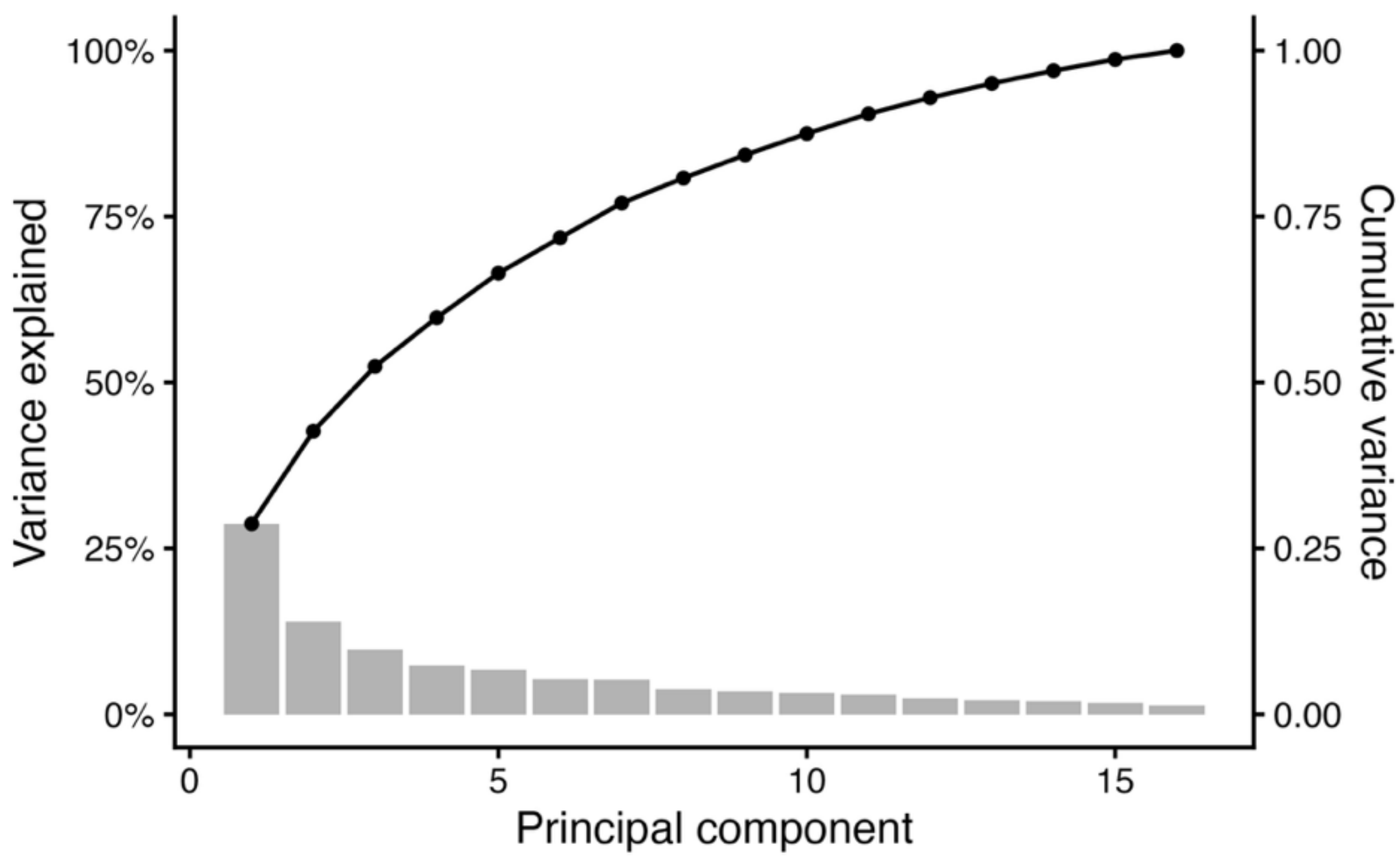

Figure S2. Means and standard deviations (SD) of PC1 loadings across 1,000 bootstrapped resampling iterations.

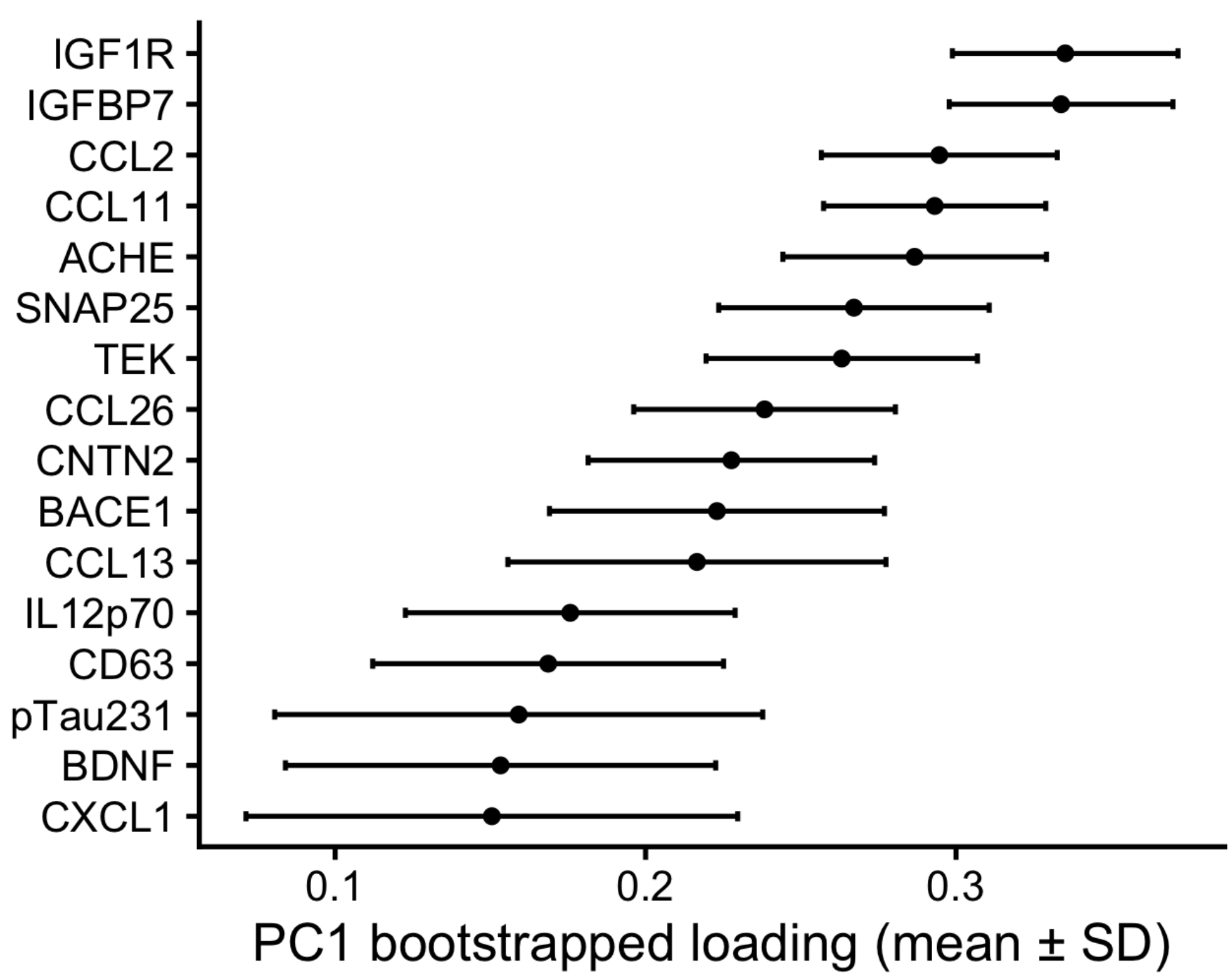

Figure S3. Bimodal distribution of serum NULISAseq APOE4 levels used to infer *APOE4* allele carriage in UCSB HAS menopause cohort.

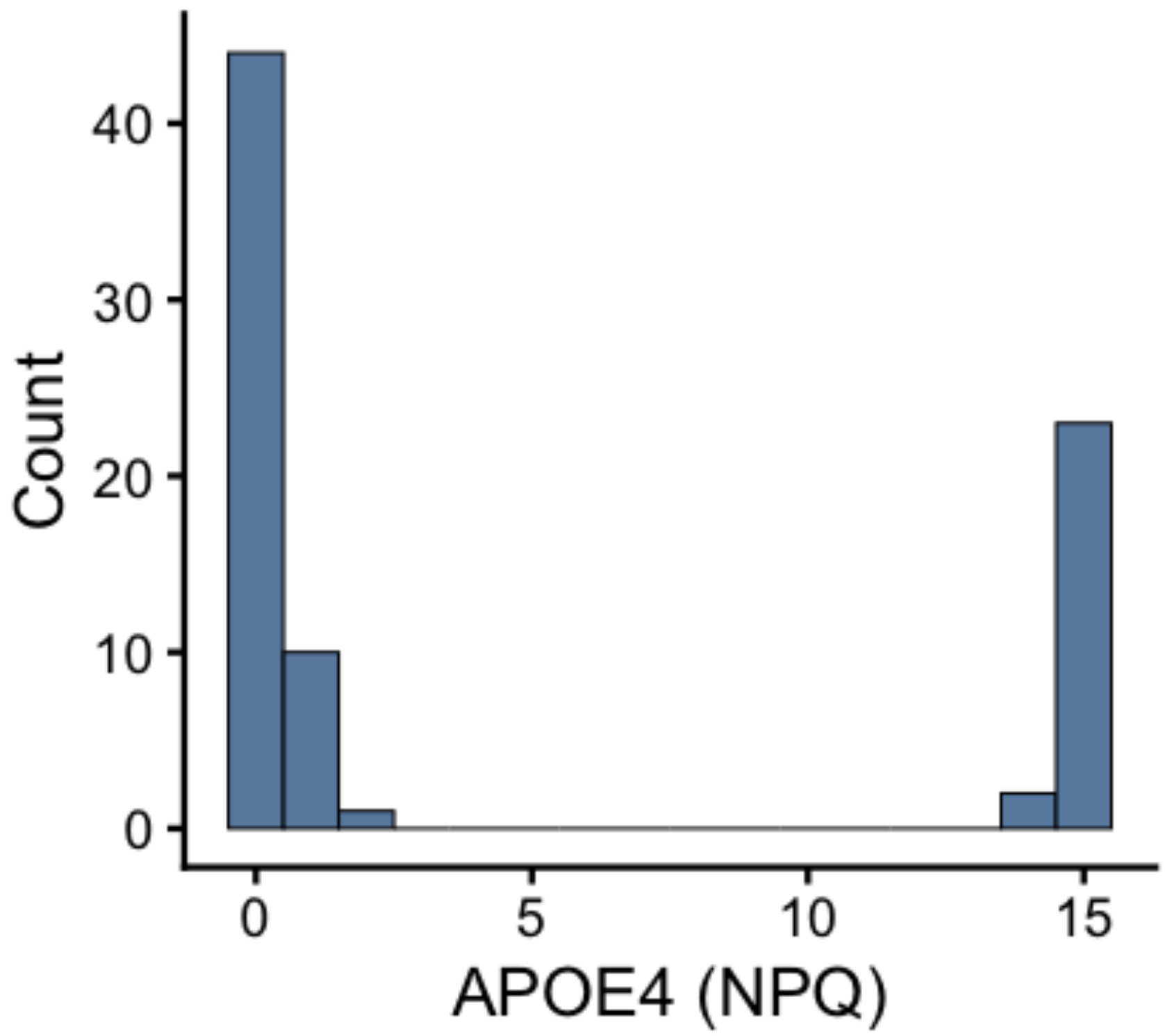

Figure S4. Imputation evaluation metrics including distribution of ground truth vs. imputed mean average error (MAE) per protein in train and test splits (left) and total MAE rate in both train and test splits (right).

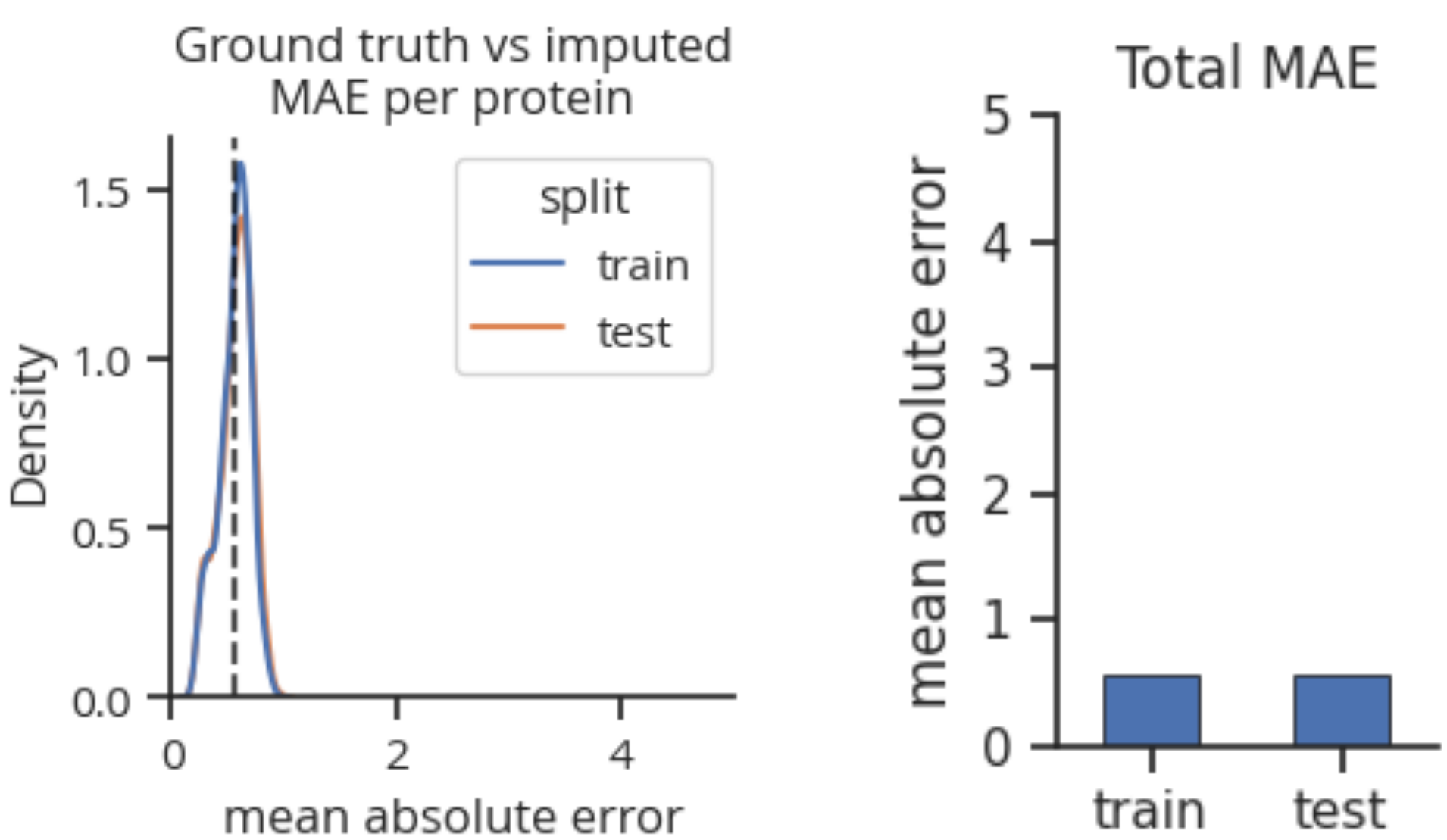

Figure S5. Comparison of PC1 loadings with and without inclusion of BDNF, SNAP25 and p-tau231.

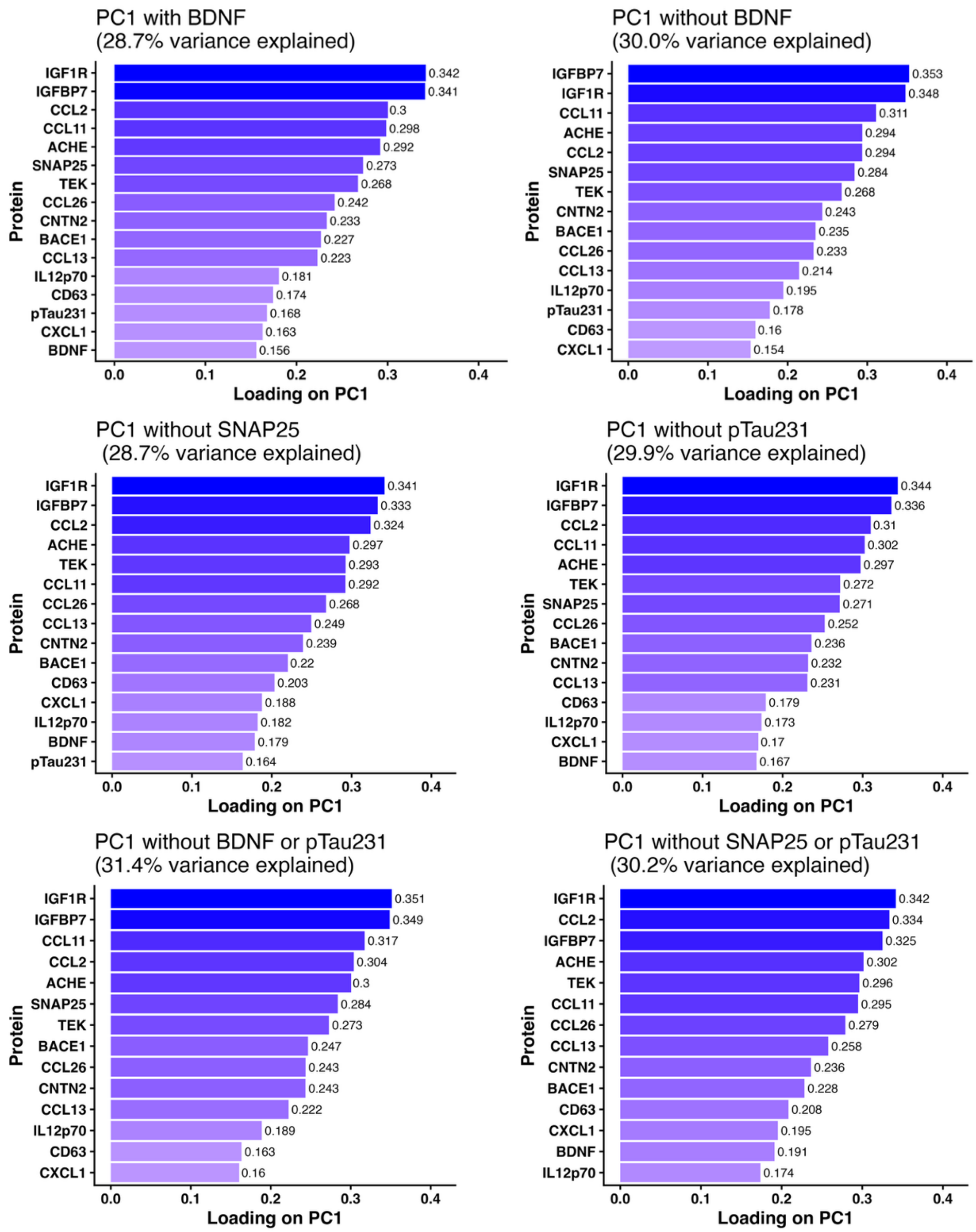

Figure S6. Comparison of log2fold change in protein levels in postmenopausal vs. premenopausal women in the UKB, in main analyses (x-axis) and sensitivity analyses (y-axis).

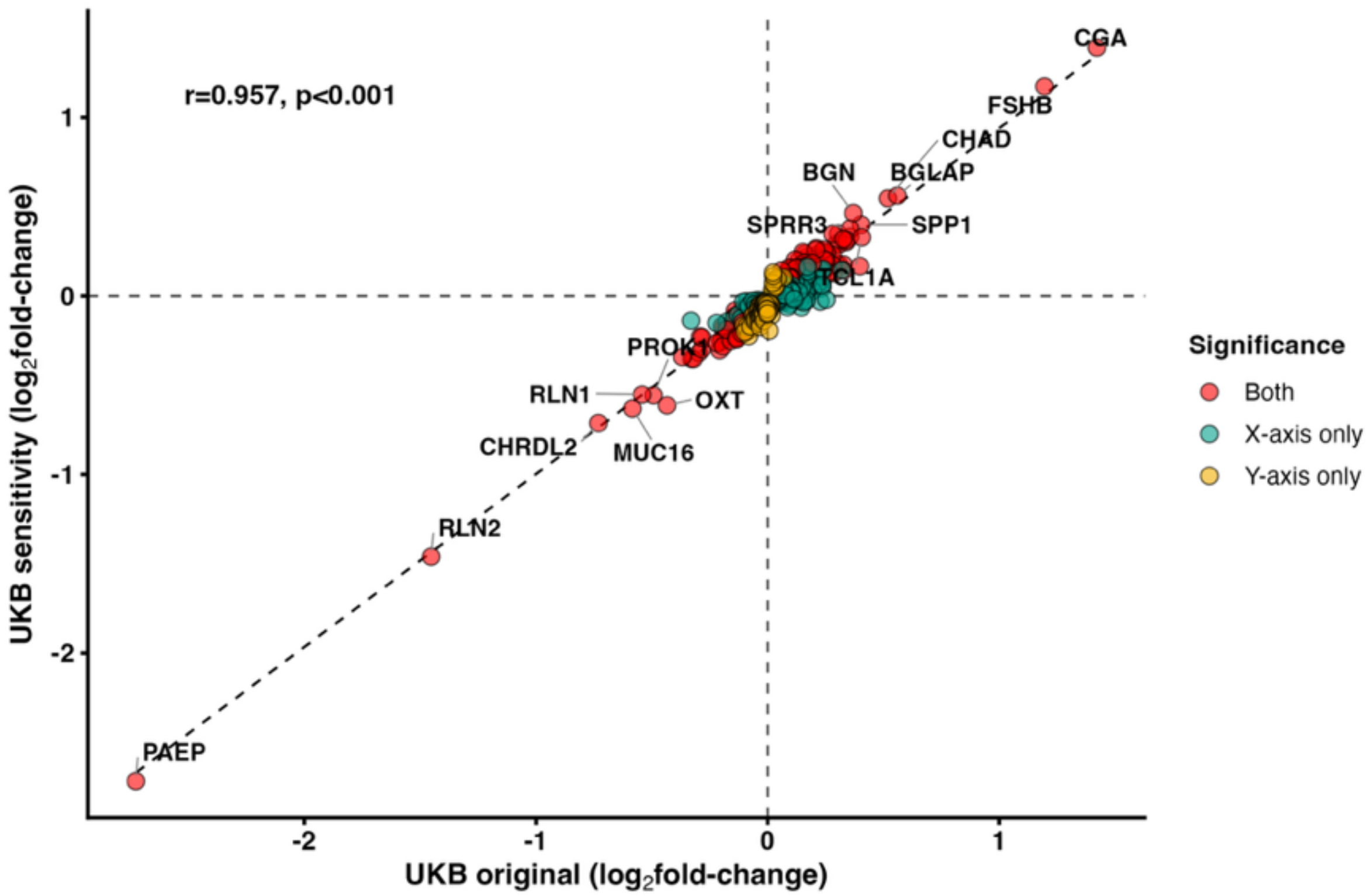

Supplement: Supplementary file 1 [file 41591_2026_4648_MOESM1_ESM.pdf]
