# Supplementary material for: Blood proteomics of menopause map to brain aging and dementia risk
Source: Nat Med. 2026 Sep 22;32(11):4244–55. doi: 10.1038/s41591-026-04648-4 (PMC13768193; doi:10.1038/s41591-026-04648-4)
Supplement: Supplementary file 2 [file 41591_2026_4648_MOESM2_ESM.pdf]

nature portfolio

Corresponding author(s): Kaitlin Casaletto, Rowan Saloner, Madeline Wood Alexander

Last updated by author(s): Aug 8, 2026

Reporting Summary

Nature Portfolio wishes to improve the reproducibility of the work that we publish. This form provides structure for consistency and transparency in reporting. For further information on Nature Portfolio policies, see our Editorial Policies and the Editorial Policy Checklist.

Statistics

For all statistical analyses, confirm that the following items are present in the figure legend, table legend, main text, or Methods section.

n/a	Confirmed	
☐	☒	The exact sample size (*n*) for each experimental group/condition, given as a discrete number and unit of measurement
☐	☒	A statement on whether measurements were taken from distinct samples or whether the same sample was measured repeatedly
☐	☒	The statistical test(s) used AND whether they are one- or two-sided<br>*Only common tests should be described solely by name; describe more complex techniques in the Methods section.*
☐	☒	A description of all covariates tested
☐	☒	A description of any assumptions or corrections, such as tests of normality and adjustment for multiple comparisons
☐	☒	A full description of the statistical parameters including central tendency (e.g. means) or other basic estimates (e.g. regression coefficient) AND variation (e.g. standard deviation) or associated estimates of uncertainty (e.g. confidence intervals)
☐	☒	For null hypothesis testing, the test statistic (e.g. *F*, *t*, *r*) with confidence intervals, effect sizes, degrees of freedom and *P* value noted<br>*Give P values as exact values whenever suitable.*
☒	☐	For Bayesian analysis, information on the choice of priors and Markov chain Monte Carlo settings
☒	☐	For hierarchical and complex designs, identification of the appropriate level for tests and full reporting of outcomes
☒	☐	Estimates of effect sizes (e.g. Cohen's *d*, Pearson's *r*), indicating how they were calculated

Our web collection on statistics for biologists contains articles on many of the points above.

Software and code

Policy information about availability of computer code

Data collection	No software was used
Data analysis	All data was analyzed using publicly available, existing R and Python packages and code repositories:<br><br>R version 4.5.0; lme4==1.1.38, survival==3.8.6, survminer==0.5.1, ggplot2==4.0.2, sjPlot=2.9.0, ggpubr==0.6.2, EnhancedVolcano==1.28.2, ggraph==2.2.2, pheatmap==1.0.13, MatchIt==4.7.2.<br><br>Python version 3.11.14; numpy==1.26.4, pandas==2.3.3, scipy==1.16.3; statsmodels==0.14.6; scikit-learn==1.8.0, matplotlib==3.10.8, seaborn==0.13.2.<br><br>GitHub(github.com/edammer)<br>GOparallel: github.com/edammer/GOparallel<br>Organ age: github.com/hamiltonoh/organageUKB<br>Cell age: github.com/dingdaisy/cellage

For manuscripts utilizing custom algorithms or software that are central to the research but not yet described in published literature, software must be made available to editors and reviewers. We strongly encourage code deposition in a community repository (e.g. GitHub). See the Nature Portfolio guidelines for submitting code & software for further information.

Data

Policy information about availability of data

All manuscripts must include a data availability statement. This statement should provide the following information, where applicable:

- Accession codes, unique identifiers, or web links for publicly available datasets
- A description of any restrictions on data availability
- For clinical datasets or third party data, please ensure that the statement adheres to our policy

Data from the UCSB Healthy Aging Study (i.e., spontaneous menopause cohort) are currently being prepared for inclusion in the Global Neurodegeneration Proteomics Consortium (GNPC) and are expected to be available to qualified researchers through the standard GNPC data access protocol in early 2027 (www.neuroproteome.org/harmonized-data-set-hds). Questions can be directed to Dr. Emily Jacobs and the Bowers WBHI team at info@wbhi.ucsb.edu. Data access requests for the other included studies can be submitted at the following links: UKB: ukbiobank.ac.uk/use-our-data/apply-for-access; WRAP: wrap.wisc.edu/data-requests-2; UCSF BrANCH: memory.ucsf.edu/research-trials/professional/open-science#data-sharing; ADNI: adni.loni.usc.edu/data-samples/adni-data. Access to controlled datasets requires submission of a formal application and adherence to the appropriate data use agreements. Requests are reviewed and responded to by the relevant data access committees according to each study's data access procedures.

Research involving human participants, their data, or biological material

Policy information about studies with human participants or human data. See also policy information about sex, gender (identity/presentation), and sexual orientation and race, ethnicity and racism.

Reporting on sex and gender	Biological sex was self-reported in all studies and distributions reported in Table 1 and Supplementary Tables S6 and S9. No data were collected on gender identity.
Reporting on race, ethnicity, or other socially relevant groupings	All studies collected data on self-reported race/ethnicity and education level, which are reported in Table 1 and Supplementary Tables S6 and S9.
Population characteristics	The UCSB Healthy Aging Study (spontaneous menopause cohort) enrolled female and male participants who were free from major medical conditions, hormone therapy use, aged 35-60, and had intact uterus/ovary(ies) (females only). Validation cohorts (UKB, ADNI, BrANCH, WRAP) included female participants aged 45 years and older, with specific population characteristics described in Extended Data and manuscript.
Recruitment	UCSB participants were recruited from the Santa Barbara community. Participants from other studies (UKB, ADNI, BrANCH, WRAP) were recruited via a combination of community recruitment, health registry invitation, & clincial referral at sites across North America and the United Kingdom.
Ethics oversight	The UCSB Healthy Aging Study (spontaneous menopause cohort) was approved by the institutional review board of the University of California, Santa Barbara (UCSB Human Subjects Committee). The UK Biobank received approval from the North West Multi-centre Research Ethics Committee and is overseen by an independent Ethics and Governance Council. WRAP was approved by the University of Wisconsin Institutional Review Board. UCSF BrANCH was approved by UCSF Institutional Review Board. ADNI was approved by local institutional review boards or equivalent ethics committees at each participating site. All participants provided informed consent.

Note that full information on the approval of the study protocol must also be provided in the manuscript.

Field-specific reporting

Please select the one below that is the best fit for your research. If you are not sure, read the appropriate sections before making your selection.

☒ Life sciences ☐ Behavioural & social sciences ☐ Ecological, evolutionary & environmental sciences

For a reference copy of the document with all sections, see nature.com/documents/nr-reporting-summary-flat.pdf

Life sciences study design

All studies must disclose on these points even when the disclosure is negative.

Sample size	UCSB: N=116; UKB: N=13,173; BrANCH: N=100; WRAP: N=93; ADNI: N=673. Sample sizes were based on maximizing use of available data in each cohort (i.e., women with plasma proteomics data) and therefore no power calculations were performed; rather, interpretation focused on replication across independent cohorts and convergent, biologically plausible patterns of findings.
Data exclusions	Described in more detail in Methods/Extended Data. For menopause models, exclusions included current use of hormone therapy or history of surgical menopause. For all models, exclusions included missing or not available proteomics data.
Replication	In the context of life sciences study design, experimental replications were not performed as no new experimental data were produced in our observational human study. Regarding analyses and results, menopause proteomic findings from UCSB were replicated in the UKB. Cognitive

	findings were replicated across four aging cohorts (UKB, ADNI, WRAP, BrANCH).
Randomization	Samples for proteomics were randomized across plates/batches according to standard procedures (see Methods).<br>No participant randomization was performed as this was an observational study design that did not include treatment groups.
Blinding	For UCSB and UCSF BrANCH NULISAseq proteomics, assays were performed on de-identified samples using coded identifiers. Laboratory personnel at the commercial testing facilities were blinded to participant phenotypes. All other proteomics included in this manuscript (WRAP, ADNI, UKB) were obtained from existing de-identified data. No new proteomic assays for these studies were performed by the authors; therefore, blinding during assay performance is not applicable to the present study.

Reporting for specific materials, systems and methods

We require information from authors about some types of materials, experimental systems and methods used in many studies. Here, indicate whether each material, system or method listed is relevant to your study. If you are not sure if a list item applies to your research, read the appropriate section before selecting a response.

Materials & experimental systems

n/a	Involved in the study	
☐	☒	Antibodies
☒	☐	Eukaryotic cell lines
☒	☐	Palaeontology and archaeology
☒	☐	Animals and other organisms
☐	☒	Clinical data
☒	☐	Dual use research of concern
☒	☐	Plants

Methods

n/a	Involved in the study	
☒	☐	ChIP-seq
☒	☐	Flow cytometry
☒	☐	MRI-based neuroimaging

Antibodies

Antibodies used	Antibodies are used in the commercially-available NULISAseq and Olink immunoassays used for proteomics.
Validation	Kits and procedures for each assay are described in the Methods and associated citations.

Clinical data

Policy information about clinical studies

All manuscripts should comply with the ICMJE guidelines for publication of clinical research and a completed CONSORT checklist must be included with all submissions.

Clinical trial registration	ADNI: NCT00106899, NCT01078636, NCT01231971, NCT02854033, NCT05617014; WRAP: NCT07222930
Study protocol	USCB HAS: Described in Methods; ADNI: clinicaltrials.gov/study/NCT00106899, clinicaltrials.gov/study/NCT01078636, clinicaltrials.gov/study/NCT01231971, clinicaltrials.gov/study/NCT02854033, clinicaltrials.gov/study/NCT05617014<br>WRAP: clinicaltrials.gov/study/NCT07222930; UKB: www.ukbiobank.ac.uk/wp-content/uploads/2025/01/Main-study-protocol.pdf<br>BrANCH: Described in Methods, more details at memory.ucsf.edu/research-trials/research/brain-aging-study
Data collection	UCSB HAS (spontaneous menopause cohort): Female and male participants aged 35 to 60 years were recruited from the Santa Barbara community between 2017 and 2022 through community advertising and local outreach. Eligible participants were free from major medical conditions and hormone therapy use and (females only) had an intact uterus and ovary(ies). Because participation was voluntary, the cohort may be subject to self-selection biases. Excluding individuals with major medical conditions, surgical menopause and hormone therapy use may limit generalizability but reduces potential confounding when investigating biological changes associated with menopause.<br><br>Validation cohorts (UKB, WRAP, ADNI, BrANCH): These analyses used existing data from deeply phenotyped observational cohorts with recruitment procedures described in detail elsewhere. The cohorts may have unique selection biases, such as healthier/higher socioeconomic status than average in the UKB and BrANCH, or enrichment for Alzheimer's disease risk (WRAP and ADNI). In the present study, replication across multiple independent cohorts with different recruitment strategies increases the robustness and generalizability of the findings.
Outcomes	Outcomes were blood-based proteomics (NULISAseq CNS, Olink), cognition (global), and incident dementia.

Plants

Seed stocks	*Report on the source of all seed stocks or other plant material used. If applicable, state the seed stock centre and catalogue number. If plant specimens were collected from the field, describe the collection location, date and sampling procedures.*
Novel plant genotypes	*Describe the methods by which all novel plant genotypes were produced. This includes those generated by transgenic approaches, gene editing, chemical/radiation-based mutagenesis and hybridization. For transgenic lines, describe the transformation method, the number of independent lines analyzed and the generation upon which experiments were performed. For gene-edited lines, describe the editor used, the endogenous sequence targeted for editing, the targeting guide RNA sequence (if applicable) and how the editor was applied.*
Authentication	*Describe any authentication procedures for each seed stock used or novel genotype generated. Describe any experiments used to assess the effect of a mutation and, where applicable, how potential secondary effects (e.g. second site T-DNA insertions, mosiacism, off-target gene editing) were examined.*
