# Supplementary material for: Blood proteomics of menopause map to brain aging and dementia risk
Source: Nat Med. 2026 Sep 22;32(11):4244–55. doi: 10.1038/s41591-026-04648-4 (PMC13768193; doi:10.1038/s41591-026-04648-4)
Supplement: Supplementary file 3 [file 41591_2026_4648_MOESM3_ESM.pdf]

nature portfolio

Peer Review File

Blood proteomics of menopause map to brain aging and dementia risk

Corresponding Author: Dr Kaitlin Casaletto

This file contains all reviewer reports in order by version, followed by all author rebuttals in order by version.

Version 0:

Reviewer comments:

Reviewer #1

(Remarks to the Author)
Thanks for asking me to review this interesting manuscript. The applicants have combined proteomic data from several cohorts (total >3000 women)
In a smaller subgroup (n=80) they report proteomics over the MT (NULISA) which was related to endocrine changes rather than age.
They also report that elevation in proteomic measures were related to poorer cognitive outcomes and AD biomarkers. These findings were replicated in Olink proteomics from age-matched women (n=2814).
They observed that menopause was releated to upregulation of inflammatory markers which might inform future biomarkers or therapautic targets to improve brain health.

Comments
The study is predicated on the assumption that menopause as a time of cognitive decline. However, the authors failed to state that (limited) prospective studies of cognition and menopause show no or minimal change in cognition (e.g. PMID: 40697165, PMID: 39081973). This may be because standard cognitive tests don't pick up subtle changes, but these findings cannot cannot be ignored. There is no point in addressing mechanisms unless there is clinical evidence of decline. The uncertainty about menopause and cognitive decline should be addressed. Also, the evidence that MHT does not prevent MCI or dementia at any age (https://pubmed.ncbi.nlm.nih.gov/41448220/). These factors argue against hormone mediated factors as the mechanism of dementia in women.

METHODS
A cross sectional group of 80 women were recruited and staged using STRAW + 10.
They state that 'menopausal symptoms' (why was irritability included??) were dichotomised as 'present' or 'absent'. It does not make clinical sense to apply this dichotomy to very different symptoms such as VMS, vaginal dryness or 'irritability". What was the justification for this dichotomy (apart from convenience)?

Proteomics - this is outside my expertise but is it valid to compare proteomics between serum and plasma from different cohorts with NULISA assays?

COHORTS INCLUDED
I was unclear whether WRAP was a prospective study? Anyway, only cross-sectional data were used in around 90 women. This approach is weak from an evidence quality perspective and would be stronger with prospective data.
BrANCH is prospective but also small (<100).
UK BB data were used to validate the findings above (n=2814). I was also unclear whether these women were studied prospectively or whether a cross sectional group of women in pre and postmenopause were included?

I am concerned about interpreting data on per/peri/post menopausal changes based on cross-sectional data.
Similarly, the statement that changes in outcomes varied by sex steroids rather than age is a critical observation. I did not fully understand how this was done (lines 204-207) and think this needs expert statisical input. Much is made of this observation so it should be robust.

Similarly, I was unclear how exactly the 'menopause proteomic signature' was defined. Is this something that is distinct

between pre and postmenopause? What about perimenopause? Is this signature apparent in those transitioning menopause (i.e. prospectively)?

RESULTS

The sample sizes are small. If they are also cross sectional it is hard for me to see how a definitive 'menopause signature' can be identified.

I applaud that comparisons with men were made but was unconvinced of the explanation that higher PC1 scores in men reflect 'androgen dominance during menopause`. Androgens don't change over the `MT and estrogens fall. Is this what androgen dominance means? I think the reasons for the observed sex difference are unknown.

The main problem in assigning symptoms/biomarker changes to menopause is that
1. Everyone gets it (so no control group) - only prospective studies can determine changes within individuals over the menopause transition

2. Menopause tends to occur at the same age, so hard to disentangle age from menopause. The fact that only p-tau231 was associated with menopause rather than age is interesting. I think statistical expertise is needed to confirm that menopause rather than age is the driver

3. I am not a statistician but don't understand why findings were adjusted for MHT use rather than MHT users considered separately? Since their sex steroid environment is distinct and the study is about changes in sex steroids, it seems odd not to report how MHT use affected the outcomes of interest.

It is interesting that no associations were seen with 'other' menopausal symptoms such as mood swings, depression and sexual dysfunction. The authors should provide clear and evidence-based information from prospective studies about why these were considered menopausal symptoms in the first place? Note that NIH (admittedly in 2005) concluded that only VMS and vaginal dryness were menopausal symptoms. I am not aware of any quality evidence that menopause at the average age is associated with depression (https://pubmed.ncbi.nlm.nih.gov/41371356/), mood swings (what does this even mean?). To my knowledge only one prospective study has measured sexual functon(SWAN https://pubmed.ncbi.nlm.nih.gov/19212271/ and POA) reports complex changes with sexual dysfunction in the minority. This may explain the lack of association with biomarkers and 'other' menopausal symptoms.

The CCL11 association with cognition was interesting. However, calling this 'cognitive decline' seems alarmist and unjustified by the findings reported. Reviewing the literature on CCL11, I was unconvinced by evidence that this is a marker of cognitive change/decline anyway (https://pubmed.ncbi.nlm.nih.gov/34320382/). Perhaps I am missing something?

DISCUSSION
I think the statement 'did not associate with age' is too strong given their data. Should be toned down.
Similarly, comment on line 396 about 'worse cognitive outcomes and elevated AD biomarker". I think one biomarker was elevated and suggest replacing the term 'worse cognitive outcomes' with a more realistic reflection of what the data actually showed.
They looked very hard for biomarkers that differed depending on reproductive stage - using primarily cross-sectional cohorts. It is incorrect to conclude that this indicated a key transition in midlife health, since women were not followed prospectively.

Reviewer #2

(Remarks to the Author)
This is a very well written important manuscript outline several circulating proteins that are associated with menopause (e.g., spontaneous, associated with vasomotor or neurologic symptoms (e.g., brain health)) in various cohorts. Interesting they also show age was not a factor. Tables were well organized and easy to read. The issues are minor but important for clarity. All issues should be easily addressed by the authors.

Minor concerns
1. Comparisons are made in cohort between data from sample types (serum/plasma) which cause the same assay to behave differently for specific analytes. If there is published data (not from manufactures) around which analytes the authors measured differ this needs to be cited and the addressed by the authors. If not, then this needs to be included as a potential analytical confounder for the study.
2. A few analytes there measured by different assay vendors. In addition to the physical technical aspects of the platform, the vendor will/could use different antibodies and thus the assay can have different LLOD and LLOQ making comparison challenging. It would be helpful to have either LLOD/LLOQ provided for these analytes as well as for all key analytes discussed as part of the proteomic signature. This is specifically an issue with Alamar (for example ptau 181 LLOD seems to be only listed in a poster) as Quanterix has most published. This is important for numerous reasons including as many assays can provide a “value” but yet the values may not be above LLOQ. In this case, the value (data) is not quantifiable and should not be used. This can differ for different assays (and sample type).
3. It was not clear if a single sample was run (or duplicates) and what QC was used for the NULISA CNS panels to ensure technical performance of the assay.
Very minor
1. The authors need to add in how samples were randomized and how they handled batch corrections for each cohort (only

mentioned for one assay) and missing data (was imputation used).
2. Although described in the text a table outlining all cohorts, assays performed, and sample type would be helpful.
3. The NULISA CNS panel has more proteins than 120. It is confusing which assay was used in the various cohorts and which (~120, 122 and 131). Please ensure that you state the specific assay and then list in supplement which protein comprised which assay. As well, please state whether there was a need to normalize across these different assays.
4. “The UK biobanks looked life menopause cohort on NULISAseq with proximity extension assay (PEA) Olink” but this is not correct. The cited paper (Ref 25) only used Olink and this is not equivalent to NULISASeq). Ref 25. NULISAseq uses a proximity ligation assay (PLA) while Olink uses proximity extension assay (PEA).
5. I am not sure the title of the paper is quite accurate. Blood-based proteomic signatures of spontaneous menopause: Implications for later-life brain aging and Alzheimer’s disease risk. The blood assay used are not conclusive for AD and perhaps other wording can be used to better reflect the interesting and important findings.

Reviewer #3

(Remarks to the Author)
The study addressed the question of which biofluid proteins are altered in association with menopause in women and whether those changes are associated with biomarkers of AD pathology in elderly women.
In a prospective study, NULISA-based protein levels were cross-sectionally compared between women who were either pre-, peri-, or post-menopausal. Results showed that a number of proteins differed between pre- and post-menopausal women at a nominal significance level, and a subsequently derived PCA score encompassing these selected proteins (i.e. menopausal protein signature) showed step-wise differences across pre-, peri-, and post-menopausal women. FSH and estradiol levels were associated with the menopausal protein signature and with some menopausal symptoms (night sweats, vaginal dryness).
In validation cohorts using already existing datasets of OLINK-derived plasma proteomics in age-matched pre- vs post-menopausal women from the UK Biobank, the alterations were confirmed, although a large number of other proteins also seemed to be altered.
In elderly women from two different cohorts (WRAP, Branch), higher protein signature scores were associated with a higher plasma p-tau/Ab42 ratio and lower cognition, although domain-specific associations could not be replicated.
Overall, the study addresses an important research gap in understanding the biological underpinnings of the menopause-related increase in AD risk. The combination of a prospective study with multiple validation samples is a strength. Also, several sensitivity analyses were conducted to exclude major confounds. Still, some concerns remain.
Comment
• The establishment of the protein signature is based on a relatively small sample, and the selection of proteins is based on nominal p-values. PC1 captured less than 25% of the variance in the selected proteins. Among the selected proteins, associations with the AD biomarkers and menopausal symptoms were often present only in a small subset when analyzed protein by protein. Is this truly a menopausal protein signature, or simply a statistical strategy to avoid the problem of multiple testing and thus low statistical power? The latter would be legitimate, but the interpretation would be different.
• The authors conclude that the menopausal proteins were not associated with age (e.g. line 394). The reviewer appreciates that sensitivity analyses were conducted in an age-restricted subset to confirm the major findings. Still, it is important to understand whether these protein changes are sensitive to age-related changes.
In the main analysis, the authors test in a partial correlation analysis whether the protein signature is associated with age after partialling out menopause status. However, given that menopause status probably reflects age differences, that statistical design is not a rigorous test of potential associations with age.
Can the authors test whether 1) there are associations with age without controlling for menopause status and 2) menopause differences after partialling out age effects?
Also, given that younger age at menopause onset has been associated with higher dementia risk in some studies (e.g. Dobson et al. Age and Ageing, 2024), age may play a modulating role rather than simply being a nuisance variable.
• In general, the control for potential confounding variables is rather sparse. Given that immunity-related proteins (e.g. CCL11) were highly represented among the proteins loading on the PC1 score, variables that may influence systemic inflammation (e.g. vascular conditions, smoking, anti-inflammatory medications) should be controlled for if available.
• Related to the above: Given that previous studies reported an APOE genotype x sex effect on tau markers, APOE may also play a role in the observed proteome alterations.
• For testing associations between the PC1 protein signature scores and AD plasma markers (p-tau181/Ab ratio), p-tau231 should always be excluded from the signature, not only in sensitivity analyses, to avoid circularity.
• The demographic, clinical, and biomarker characteristics of the WRAP and Branch cohorts should be described in the main body of the article. What were the biomarker and cognitive status of these participants, rather than functional status? MCI patients may still be functionally normal.
• The authors mention that 9 out of 13 proteins from the discovery cohort also differed between pre- and post-menopausal women in the UK Biobank, suggesting high reproducibility. However, Figure 6B suggests that most proteins showed either up- or downregulation in the UK Biobank. Could the authors compute the likelihood that 9 out of 13 proteins from the protein signature would also show elevations just by chance, given the proportion of altered proteins from the Olink assay in the UK Biobank? The concern is that, due to the high number of menopause-related protein alterations in the UK Biobank, most proteins from the signature may appear altered just by chance, along with many others.
• Do the protein alterations have any predictive value? This could potentially be assessed in the UK Biobank. Do women with higher protein signature values have a higher risk of (AD) dementia?

Version 1:

Reviewer comments:

Reviewer #1

(Remarks to the Author)
The authors have largely addressed the issues raised. The addition of further cohorts is welcome.

I am unhappy about the suggestion that cognitive symptoms (largely 'brain fog) over the menopause transition is somehow related to cognitive decline which is then related to dementia. This is a very long bow and the data do not support these associations. As well as being inaccurate, this kind of conflation risks alarming women who may be concerned that cognitive symptoms at menopause indicate an increased risk of dementia. As the authors note, objective measures of cognition at menopause do not fall outside the normal (impaired) range. That makes them normal. The authors have made interesting and important observations and do not need to artificially inflate these by over-calling their findings, especially when this may do harm.

I suggest that the language around cognition is clarified throughout the paper. For example "cognitive decline" is not an appropriate term for changes in cognitive function within the normal range. Please be mindful about accurate language to describe your findings.

Similarly, I was unconvinced by the broad statement about menopausal symptoms being "brain related". It is well established that VMS arise from the hypothalamus. Sleep and mood are also brain related but are common independent of menopause.

Similarly, the BMS statement that MHT does not restore estrogen concentrations to premenopausal levels seems rather odd. Postmenopausal women have physiologically lower estrogen levels than premenopausal women. There is no clinical evidence that "restoring" levels to those of premenopause is beneficial and consistent evidence that it may do harm (VTE, stroke etc). Also, "restoring" estrogen means adding progesterone (for most women) which is unphysiological in postmenopausal women and confers additional risk (breast cancer)

Reviewer #2

(Remarks to the Author)
The authors have carried out an excellent revision. They have addressed all concerns within the manuscript providing information needed to clarify the analysis they have done. I still remain surprised that serum vs plasma essentially correlated but am happy with how the authors deal with this concern.

Reviewer #3

(Remarks to the Author)
The authors have adequately addressed all concerns. My only suggestion at this point is that in the newly added Figures 6B and C, data points (spaghetti plots) instead of error bars only are shown. This would allow to better appreciate the spread of the data. Figure 6B shows that error bars do not always reflect the full extent of variability in the data.

Open Access This Peer Review File is licensed under a Creative Commons Attribution 4.0 International License, which permits use, sharing, adaptation, distribution and reproduction in any medium or format, as long as you give appropriate credit to the original author(s) and the source, provide a link to the Creative Commons license, and indicate if changes were made.
In cases where reviewers are anonymous, credit should be given to 'Anonymous Referee' and the source.
The images or other third party material in this Peer Review File are included in the article's Creative Commons license, unless indicated otherwise in a credit line to the material. If material is not included in the article's Creative Commons license and your intended use is not permitted by statutory regulation or exceeds the permitted use, you will need to obtain permission directly from the copyright holder.
To view a copy of this license, visit https://creativecommons.org/licenses/by/4.0/

Editor:

Editorially, we ask that you address the issues raised by reviewers concerning the limited number of samples in the cohorts, ideally by increasing sample sizes, or integrating proteomics from other cohorts to increase the robustness of the findings. Additionally, we ask that you address the comments raised by reviewer 1 regarding the assumption that menopause is linked to cognitive decline, and better frame the work in light of the most current literature.

Response:

Thank you for the opportunity to revise and resubmit our manuscript. We carefully considered all editorial and reviewer feedback and have integrated several new datasets, additional analyses, and revised framing of the study questions that we believe substantially strengthen the contribution of this manuscript to the literature. These changes are explained in detail in the response below and summarized here:

- In response to the request to increase the sample size, we have added proteomics and clinical data from two additional large longitudinal aging cohorts (ADNI, N=673; UKB, N=11,059) to show that the menopause proteomics we identified consistently associate with poorer cognitive outcomes across independent cohorts of postmenopausal women an average age of 61-72 (longitudinal global cognitive decline, cross-sectional global cognition, incident Alzheimer's disease (AD) dementia, as available). This is in addition to the two existing, deeply phenotyped older adult cohorts (UCSF BrANCH and WRAP) from our original submission. Together, these findings suggest that biological signals identified in blood during the midlife menopause transition demonstrate robust clinical relevance for cognitive aging across 4 independent, older adult cohorts including almost 12,000 cumulative participants (Fig.6).

Across four independent aging cohorts, higher menopause proteomic scores (i.e., PC1) were associated with poorer cognitive outcomes.

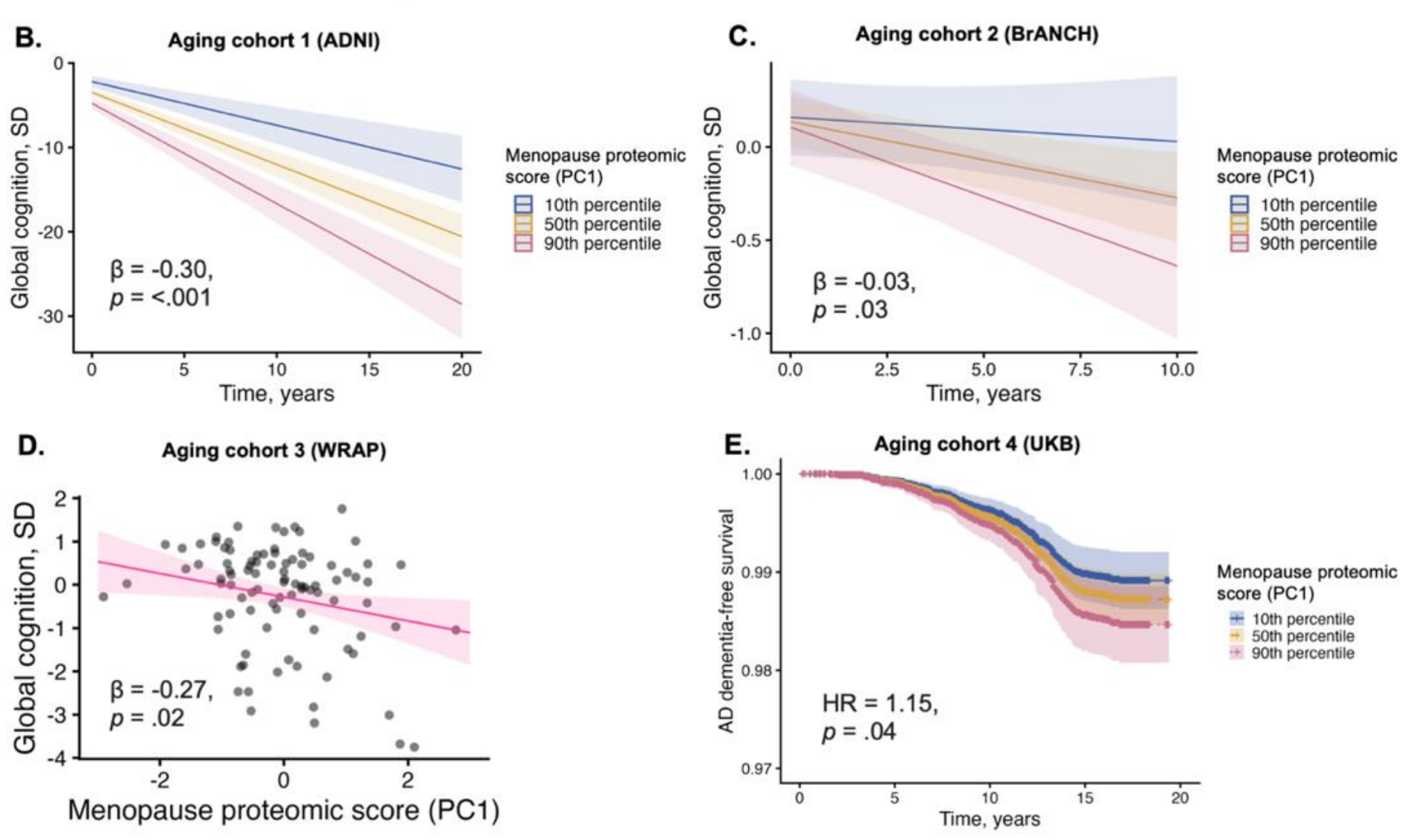

- To increase rigor and further validate the biological robustness of the observed menopause-related proteomic shifts, we performed additional analyses in the UK Biobank examining age-matched pre/peri- versus postmenopausal women (N=2,814). Specifically, instead of focusing only on individual proteins, we now explicitly test whether menopause proteomic scores differ by menopause stage and found robust replication (β=0.22, *p*<.001). This confirms that this composite score captures reproducible and coordinated menopause-associated biological shifts. Additionally, we now show that in the UKB, postmenopausal (vs. pre/perimenopausal) women exhibit accelerated multi-organ and cellular aging on previously established proteomic clocks (Oh et al., Nature Medicine, 2025; Ding et al; Nature Medicine; 2026), including brain aging and oocyte aging (Fig.4G-H). These findings support the validity of our menopause classifications in the UKB and provide additional broader evidence that the menopause transition is associated with accelerated biological aging changes that are relevant for the brain and later dementia risk.

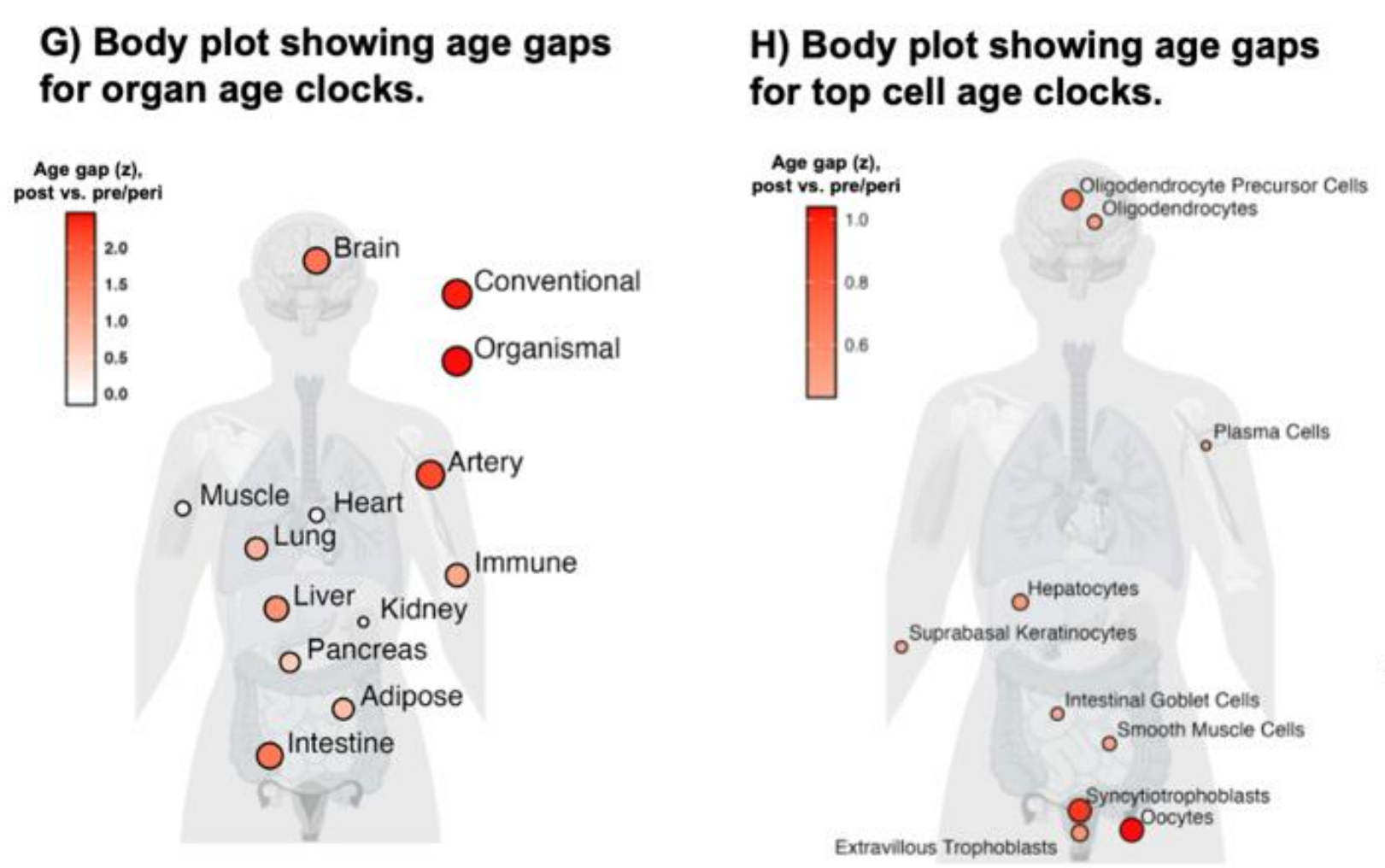

- Finally, in response to feedback from Reviewer 1, we have reframed the introduction and rationale to better capture the contemporary literature on menopause and brain health. Specifically, we acknowledge that menopause does not induce frank cognitive impairment but also underscore the evidence linking menopause to neurologic biology (please see Reviewer 1, response 1).

<u>Reviewer #1: menopause, OBGYN</u>

Thanks for asking me to review this interesting manuscript. The applicants have combined proteomic data from several cohorts (total >3000 women)
In a smaller subgroup (n=80) they report proteomics over the MT (NULISA) which was related to endocrine changes rather than age.
They also report that elevation in proteomic measures were related to poorer cognitive

outcomes and AD biomarkers. These findings were replicated in Olink proteomics from age-matched women (n=2814).
They observed that menopause was releated to upregulation of inflammatory markers which might inform future biomarkers or therapautic targets to improve brain health.

Comment 1. The study is predicated on the assumption that menopause as a time of cognitive decline. However, the authors failed to state that (limited) prospective studies of cognition and menopause show no or minimal change in cognition (e.g. PMID: 40697165, PMID: 39081973). This may be because standard cognitive tests don't pick up subtle changes, but these findings cannot be ignored. There is no point in addressing mechanisms unless there is clinical evidence of decline. The uncertainty about menopause and cognitive decline should be addressed. Also, the evidence that MHT does not prevent MCI or dementia at any age (https://pubmed.ncbi.nlm.nih.gov/41448220/). These factors argue against hormone mediated factors as the mechanism of dementia in women.

Response 1. We thank the reviewer for their constructive feedback, including the opportunity to improve our framing and clarify our rationale for this study. We have rewritten the introduction to better reflect both our aims and the contemporary literature (see p. 3-4). Namely, we agree that menopause is not associated with frank cognitive impairment or decline outside of the normative range. Nonetheless, both of the studies cited by this reviewer show evidence that there are in fact brain-related changes occurring during menopause (not pathologic); Price et al 2025 shows mood, vasomotor symptoms, and sleep disturbances, all of which have direct links to brain functioning, while Than et al. 2023 showed objective performance decline on cognitive processing speed. We also direct the reader to a recent scoping review of the cognitive symptoms of menopause in Lancet OB/GYN and Women's Health (DOI: 10.1016/S3050-5038(26)00043-9) showing that in fact a majority of women (~two-thirds) report experiencing cognitive symptoms during (peri)menopause, and at least 6 prospective studies demonstrate measurable cognitive decline over menopause (that does not fall in the "impaired" range). For further information on cognitive changes during menopause, please see also: Caldwell et al., Brain and cognitive health and midlife. (2026) DOI: 10.1097/GME.0000000000002804 and Maki et al., Brain fog in menopause: a health-care professional's guide for decision-making and counseling on cognition (a 2022 commissioned International Menopause Society White Paper on cognition) DOI:10.1080/13697137.2022.2122792.

We further underscore evidence linking menopause to neurologic biology. That is: 1) we highlight that the cardinal symptoms reported by up to 80% of women are brain related, including vasomotor symptoms, sleep disturbances, reported cognitive and mood changes (e.g., PMID: 33071945; DOI: 10.1016/S3050-5038(26)00043-9, https://doi.org/10.1038/s44159-025-00463-9); 2) there is preclinical evidence that manipulating brain centers along the HPG-axis can *induce* menopause, highlighting a causal role for the brain in menopause biology (e.g., PMIDs: 30497015, 19063938); and 3) longitudinal findings show within-person decline in cognitive performance (not impairment) across menopause even adjusting for age, including as noted by this

reviewer (summarized in DOI: 10.1016/S3050-5038(26)00043-9). Together, this body of work strongly suggests that examining the neurology of menopause (though not pathologic) is highly warranted. This is akin to studying brain changes during the endocrine transitions of adolescence – though not pathologic, highly warranted to inform inflection points for when children may be at risk for adverse cognitive and mental health outcomes; for instance, the ABCD Study represents a $1 billion public-private investment to map brain and cognitive changes over the endocrine transition of adolescence and has yielded highly clinically actionable findings (reviewed in PMID: 32541809).

Of note, our study is not designed, nor do we aim to uncover the biology underlying the subtle cognitive changes that occur during the menopause transition itself. Instead, we position menopause as a *neurologic transition* that has relevance for later life brain aging. This is particularly relevant given the increasing number of studies demonstrating that earlier age of menopause, cause of menopause, and symptom burden associates with increased dementia risk – directly linking these two phenomena (e.g., PMID: 37010830, PMID: 24336141, PMID: 36224031). In this framework, menopause offers an opportunity for basic discovery to understand earliest shifts in brain aging during midlife that may uncover biology relevant both for women's later life brain health and potentially for all aging brains. Further, identification of biological risk factors during this midlife transition that are relevant in later life has massive potential implications for intervention opportunities to prevent dementia in women.

We believe the lack of evidence for hormone therapy in preventing dementia does not negate the possibility that midlife neuroendocrine changes influence later life health outcomes. Notably, hormone therapy does not restore levels to the premenopausal endocrine environment or reverse all the physiological consequences of menopause (e.g., https://thebms.org.uk/wp-content/uploads/2025/11/24-NEW-BMS-ToolsforClinicians-Measurement-of-serum-estradiol-JULY2025-C.pdf; DOI: 10.1097/GME.0000000000002459). We hope this reframing clarifies our rationale and more accurately represents the current scientific consensus on menopause and brain health.

Comment 2. METHODS A cross sectional group of 80 women were recruited and staged using STRAW + 10. They state that 'menopausal symptoms' (why was irritability included??) were dichotomised as 'present' or 'absent'. It does not make clinical sense to apply this dichotomy to very different symptoms such as VMS, vaginal dryness or 'irritability''. What was the justification for this dichotomy (apart from convenience)?

Response 2. We apologize for the lack of clarity in our original wording. Each menopause symptom was separately dichotomized as present or absent (e.g., night sweats present vs. night sweats absent), rather than globally dichotomizing all symptoms as present or absent. We applied this dichotomy due to the small sample sizes and low statistical power available to examine frequency-dependent associations with menopause symptoms, as indicated in the table below:

	Peri (N=25)	Post (N=24)
Hot flashes in the last 2 weeks (%)		
Not at all	14 (56.0)	8 (33.3)
1-5 days	6 (24.0)	10 (41.7)
6-8 days	1 (4.0)	0 (0.0)
9-13 days	2 (8.0)	1 (4.2)
Every day	2 (8.0)	5 (20.8)
Night sweats in the last 2 weeks (%)		
Not at all	9 (36.0)	13 (54.2)
1-5 days	11 (44.0)	6 (25.0)
6-8 days	2 (8.0)	0 (0.0)
9-13 days	2 (8.0)	2 (8.3)
Every day	1 (4.0)	3 (12.5)
Vaginal dryness in the last 2 weeks (%)		
Not at all	19 (76.0)	16 (66.7)
1-5 days	4 (16.0)	4 (16.7)
6-8 days	1 (4.0)	1 (4.2)
9-13 days	1 (4.0)	0 (0.0)
Every day	0 (0.0)	3 (12.5)
Irritability in the last 2 weeks (%)		
Not at all	6 (24.0)	9 (37.5)
1-5 days	15 (60.0)	11 (45.8)
6-8 days	2 (8.0)	0 (0.0)
9-13 days	2 (8.0)	3 (12.5)
Every day	0 (0.0)	1 (4.2)

This has been clarified on page 13:

> “Female participants self-reported how often they experienced commonly endorsed menopause symptoms[44,45] including hot flashes, night sweats, vaginal dryness, and irritability in the last two weeks, on a scale of “not at all”, “1-5 days”, “6-8 days”, “9-13 days”, or “every day”. Due to the small sample size, each individual symptom was dichotomized as present versus absent.”

The scope of symptoms women may experience during menopause is not fully understood and still under active investigation. Irritability was included given it is one of the most common self-report symptoms of menopause endorsed by 60-80% of women in large studies and meta-analyses (PMID: 41603602, 38956480), now cited in the text on page 13.

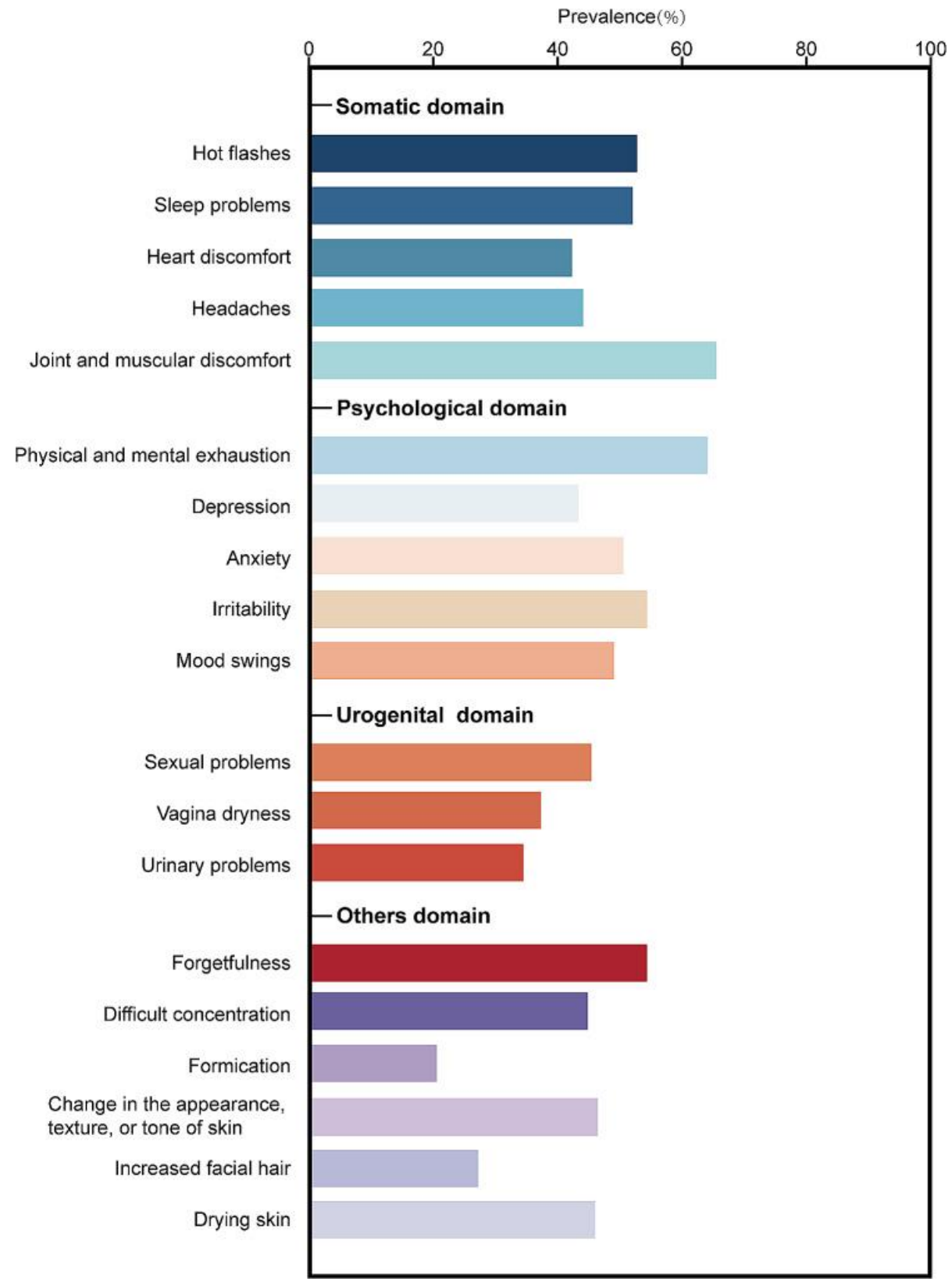

For further reference, please see above figure 2 from Fang et al. meta-analysis (PMID: 38956480) showing pooled estimate prevalence of nineteen menopausal symptoms among middle-aged women.

Comment 3. Proteomics - this is outside my expertise but is it valid to compare proteomics between serum and plasma from different cohorts with NULISA assays?

Response 3. Thank you for raising this important point (see also Reviewer 2, comment 1). Assay matrices (i.e., plasma vs. serum) were selected based on sample/data availability. The NULISA CNS assay is relatively new, having been commercially launched in 2024, but recent data report comparable performance of this assay in serum and plasma (PMID: 42000813). This article shows high target detectability (>95% in both serum and plasma) and moderate-to-strong correlations for the vast majority (84%) of the ~120 targets measured, including all 16 identified menopause proteins. Serum may show slightly stronger inflammatory signals and slightly weaker neurodegenerative signal relative to plasma. This data supports the use of both plasma and serum samples for NULISA proteomics as in our study. Further, our ability to both replicate and detect consistent relationships with the menopause proteomic signature across multiple independent cohorts, sample matrices, and even analyte platforms support the high rigor of the targets we have identified. Nonetheless, we acknowledge challenges of sample matrix differences as a potential limitation (p. 12):

> "Furthermore, while serum and plasma appear to perform comparably on the NULISAseq CNS panel,[43] matrix differences may influence protein measurements making cross-cohort comparison more challenging."

Comment 4. COHORTS INCLUDED I was unclear whether WRAP was a prospective study? Anyway, only cross-sectional data were used in around 90 women. This approach is weak from an evidence quality perspective and would be stronger with prospective data.
BrANCH is prospective but also small (<100).
UKBB data were used to validate the findings above (n=2814). I was also unclear whether these women were studied prospectively or whether a cross sectional group of women in pre and postmenopause were included?

Response 4. We agree that the cohorts included in the original submission were small and that cross-sectional data have inherent limitations in terms of quality of evidence. To address this, we have added analyses from two additional large aging cohorts with proteomics and longitudinal cognitive and clinical data. Together, these four cohorts encompass almost 12,000 post-menopausal women. To refocus our analyses on the most clinically relevant outcomes that are consistent across cohorts, we now show that the menopause proteomic score (PC1) associates with (1) global longitudinal cognitive decline in BrANCH and ADNI; (2) global cross-sectional cognition in WRAP; and (3) incident AD dementia in the UK Biobank. These findings are described on page 9 and depicted in Figure 6:

> "In longitudinal data with an average 5-year follow up from Alzheimer's Disease Neuroimaging Initiative (ADNI; *N*=673 total; 54.3% MCI/dementia; 49.0% amyloid-positive) and UCSF Brain Aging Network for Cognitive Health (BrANCH; *N*=100; 1% MCI; 21.7% amyloid-positive), higher (i.e., more postmenopausal) baseline menopause proteomic scores associated with faster global cognitive decline (ADNI: β=-0.30, 95% CI=-0.42, -0.18, *p*<.001; BrANCH: β= -0.03, 95% CI= -0.05, -0.002, *p*=.03; Fig.6B-C). In cross-sectional data from WRAP (*N*=93; 15.0% MCI/dementia; 64.5% amyloid-positive), higher menopause proteomic scores associated with worse global performance (β= -0.27, 95% CI= -0.51, -0.04, *p*=.02; Fig.6D). Finally, in the UKB (*N*=11,059), we examined whether menopause proteomic scores associated with risk for incident dementia over a mean (*SD*) of 15.7 (2.84) years of follow-up. Higher menopause scores were associated with greater risk for AD dementia (HR=1.15, 95% CI=1.01, 1.31, *p*=.04, *N*=219 events; Fig.6E), but not frontotemporal (HR=0.90, 95% CI=0.59, 1.36, *p*=.61, *N*=23 events), vascular (HR=1.15, 95% CI=0.91, 1.47, *p*=.25, *N*=65 events) or all-cause dementia (HR=1.08, 95% CI=0.98, 1.19, *p*=.13, *N*=387 events)."

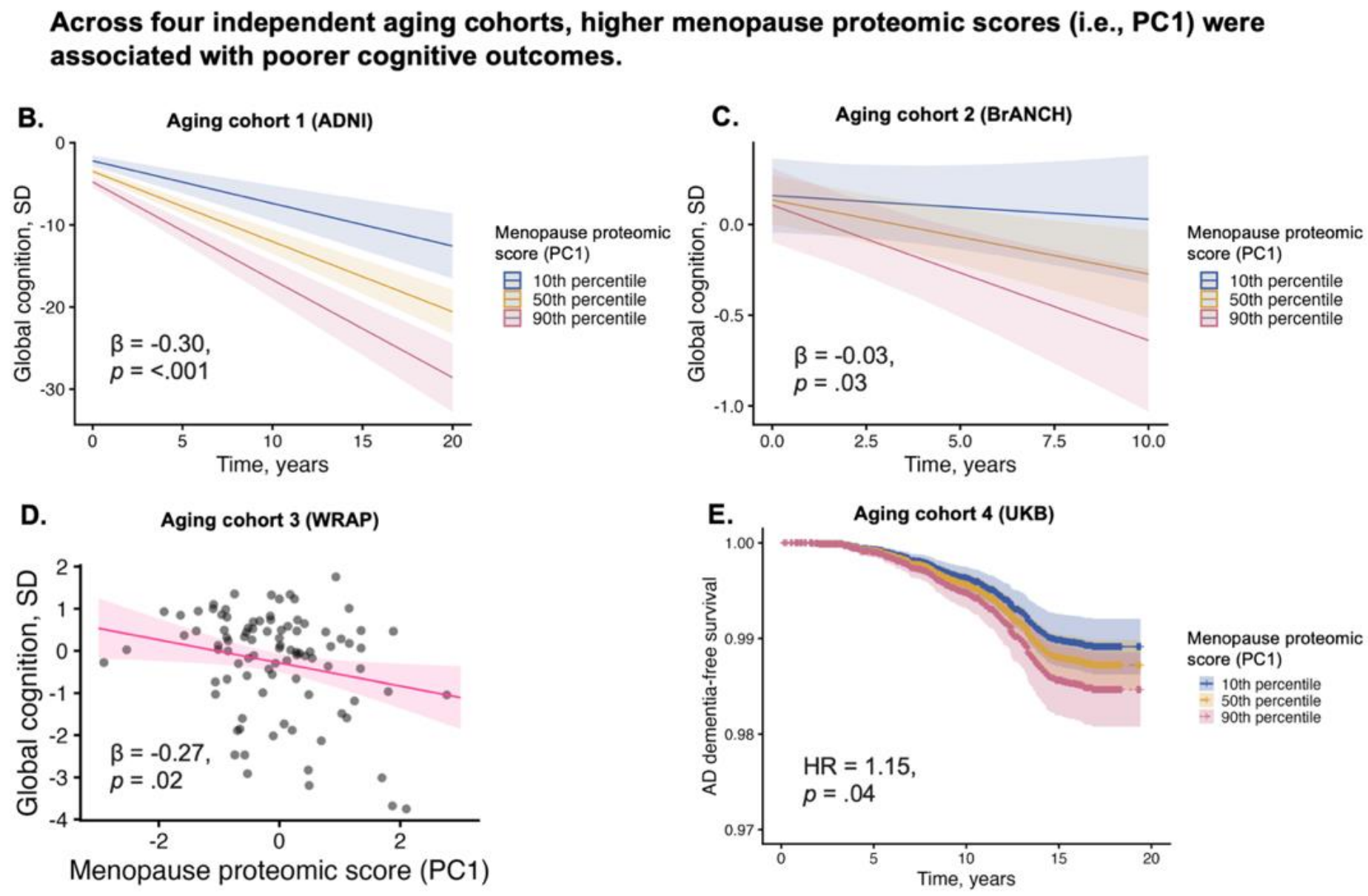

The UK Biobank validation cohort of age-matched pre/peri- and postmenopausal women was cross-sectional, due to very limited longitudinal proteomics data available in the UKB at this time. This is clarified in the methods (p. 14):

> “To evaluate whether spontaneous menopause proteomic shifts were robust to cohort and proteomic platform, we compared spontaneous menopause protein effect sizes detected in the midlife menopause cohort with NULISAseq proteomics to menopause protein effect sizes detected in a large cohort of 2,814 women from UK Biobank with cross-sectional proximity extension assay Olink proteomics (Extended Data).[48]”

We further acknowledge lack of longitudinal data across the menopause transition itself as a key limitation and important future direction (see Responses 5 and 8).

Comment 5. I am concerned about interpreting data on per/peri/post menopausal changes based on cross-sectional data.

Response 5. We agree that the lack of longitudinal data is a limitation of the study which we now include in the Discussion (see text below). Studies examining within-person proteomic shifts are actively underway by our group and others and will be highly informative. However, given the high novelty and rigor of our findings with replication over 4 cohorts, we believe these data provide an important foundation to build on.

> **“**Other limitations include the small spontaneous menopause discovery sample which only included cross-sectional protein and hormone data […] Longitudinal studies mapping the within-person trajectories of hormones, multi-omics, symptoms, and brain health across menopause will be critical to confirm the observed menopause-associated proteomic changes and disentangle the

complex interplay between endocrine, neurologic, and chronological aging." (p. 12)

Comment 6. Similarly, the statement that changes in outcomes varied by sex steroids rather than age is a critical observation. I did not fully understand how this was done (lines 204-207) and think this needs expert statistical input. Much is made of this observation so it should be robust.

Response 6. We agree that this is an important component of the study and inferences from observational human data have inherent limitations. We now clarify that our analysis does not establish that hormone-related variation in the proteome occurs entirely independent of aging or is causal. Rather, the multivariable models indicate that hormone levels explain more variance in the menopause proteomic score than age when modelled together. Further, our finding that age did not correlate with menopause associated targets in age-matched men suggests that chronological age is likely having a smaller effect (compared to menopause stage). We have revised the text accordingly (p. 6):

> "Age-adjusted models showed the menopause proteomic score inversely associated with estradiol (β=-0.89, 95% CI=-1.37, -0.41, p=.0004), positively associated with FSH (β=1.29, 95% CI=0.78, 1.80, p<.0001), and did not associate with progesterone (β=-0.23, 95% CI=-0.71, 0.25, p=.34) (Fig.2C). When simultaneously modeling all three hormones together, only FSH was significantly associated with the menopause score (FSH: β=1.13, 95% CI=0.51, 1.78, p=.0005; estradiol: β=-0.43, 95% CI=-0.97, 0.11, p=.12; progesterone: β=0.24, 95% CI=-0.20, 0.68, p=.28). Furthermore, in the same multivariable model, age was not associated with the menopause score (β=-0.01, 95% CI=-0.17, 0.14, p=.88), suggesting that the menopause score is more strongly related to hormone levels than to age when modelled together."

> "We computed the same composite score in men and found age did not correlate to the menopause score in men (cor=-0.02, p=.89)."

We have also softened our language regarding menopause-associated proteomic shifts, hormones, and age in the revised passages below:

> "In N=80 rigorously staged (STRAW+10) -, peri-, and postmenopausal women with serum NULISAseq proteomics, spontaneous menopause was characterized by dysregulation in inflammatory, synaptic, metabolic, and Alzheimer's disease biologic processes, which tracked more strongly with hormones than with age." (from Abstract)

> "Menopause-related proteomic shifts tracked more strongly with hormones than with age." (p. 10)

Comment 7. Similarly, I was unclear how exactly the 'menopause proteomic signature' was defined. Is this something that is distinct between pre and postmenopause? What about perimenopause? Is this signature apparent in those transitioning menopause (i.e. prospectively)?

Response 7. We appreciate the opportunity to clarify the goal of the proteomic score. We agree that our study did not holistically identify all possible proteomic shifts of menopause and is limited by small sample size and lack of longitudinal data (also see Reviewer 3 response 1). Instead, we aimed to identify biologic shifts of menopause relevant to later life brain aging using a circumscribed proteomics panel that includes targets known to be relevant to brain aging (NULISA CNS 120). We used PC1 not as an all-encompassing menopause protein signature but as a statistical strategy to reduce data dimensionality/noise introduced by the protein measures, and to capture menopause-related biological changes observed in our data with a single measure that mitigates the problem of multiple comparisons. To clarify this, we have renamed PC1 as a “menopause proteomic composite” rather than a “signature.” This rationale is now stated clearly on page 5:

> “To reduce data dimensionality and noise, more holistically capture biology represented, and mitigate multiple comparisons, we performed principal components analysis (PCA) on these 16 targets to estimate a menopause proteomic composite score that summarizes these top menopause-related proteomic changes.”

Comment 8. RESULTS. The sample sizes are small. If they are also cross sectional it is hard for me to see how a definitive 'menopause signature' can be identified.

Response 8. We agree that the small sample size and lack of longitudinal data are limitations of the study, and we now explicitly acknowledge these in the Discussion (p.12; see Response 5). We also agree that the menopause proteomic score does not represent a definitive menopause signature and have updated the wording and interpretation of the manuscript to reflect this (see Response 7).

Comment 9. I applaud that comparisons with men were made but was unconvinced of the explanation that higher PC1 scores in men reflect 'androgen dominance during menopause`. Androgens don't change over the `MT and estrogens fall. Is this what androgen dominance means? I think the reasons for the observed sex difference are unknown.

Response 9. To avoid speculation, we have removed this sentence from the results.

Comment 10. The main problem in assigning symptoms/biomarker changes to menopause is that
1. Everyone gets it (so no control group) - only prospective studies can determine changes within individuals over the menopause transition
2. Menopause tends to occur at the same age, so hard to disentangle age from

menopause. The fact that only p-tau231 was associated with menopause rather than age is interesting. I think statistical expertise is needed to confirm that menopause rather than age is the driver

Response 10. We agree with these challenges and appreciate the importance of deeply interrogating associations with menopause stage vs. age. We did include age-matched men in our original analysis which can serve as a control for disentangling effects of chronological age versus endocrine status. As noted in Response 6, we did not observe a statistically significant relationship between age and menopause targets identified in age-matched men, suggesting that overarching chronological aging biology does not wholly explain the targets identified (including p-tau231). We further emphasize that these are midlife men and women with a restricted age range (43-58) further limiting potential influence of chronological age on the targets identified.

Nonetheless, we now include additional analyses testing the unadjusted vs. age adjusted associations with protein levels in women, which are now described in the Results (p.5-6) and shown in Figure 1E and Table S4 (also see response to Reviewer 3, comment 2). In brief, we found that there were significant associations between some of the menopause proteins and age in women when unadjusted for menopause status. This is expected given that menopause stage and age are inherently confounded. However, in multivariable models considering both age and menopause status, age is no longer associated with PC1 scores. We also compared the unadjusted effect sizes of age versus menopause stage on PC1 scores to contextualize potential effects sizes of each. Here, we show that the effects of menopause stage are notably stronger than those of age. Together, these findings suggest that menopause stage has statistically stronger and larger effect sizes than chronological age with the identified menopause proteins. These findings are described in the text as below. Additionally, we added the cross-sectional design as a limitation acknowledged in the discussion (see Reviewer 1, Response 5).

> "In women, age was significantly associated with higher menopause proteomic scores (cor=0.36, *p*=.001). However, this association with age was no longer significant and reversed directionality after adjusting for menopause stage (β=-0.14, 95% CI=-0.31, 0.03, *p*=.11). Unadjusted linear estimates showed a stepwise effect of menopause stage on the proteomic score. Relative to premenopausal, perimenopausal status was associated with a 1.36-unit (*SD*) increase in menopause scores, and postmenopausal status with a 3.25-unit increase. By contrast, each year of age was associated with a 0.24-unit higher menopause score. To assess the potential modulating role of age, we also tested interactions between menopause stage and chronological age on the menopause score, which were not significant (post [vs. pre]*age: β=0.003, 95% CI= 0.44, 0.45, *p*=.99; peri [vs. pre]*age: β=0.17, 95% CI=-0.24, 0.57, *p*=.42). We computed the same composite score in men and found age did not correlate to the menopause score in men (cor=-0.02, *p*=.89). Overall, these findings suggest that the observed proteomic changes were driven more by menopause stage than chronological age in women." (p. 5-6)

Comment 11. 3. I am not a statistician but don't understand why findings were adjusted for MHT use rather than MHT users considered separately? Since their sex steroid environment is distinct and the study is about changes in sex steroids, it seems odd not to report how MHT use affected the outcomes of interest.

Response 11. Thank you for this comment. We would like to clarify that main analyses of menopause-associated proteomic signals were restricted to women with no current use of MHT in both the UCSB spontaneous menopause cohort and in the UK Biobank. We restricted to women without exogenous hormone use to understand the proteomic shifts of spontaneous menopause, reflecting endogenous endocrine changes. The reviewer is correct that analyses of menopause symptoms in aging women were adjusted for MHT use. This decision was made given that MHT use may differ systematically between women with and without menopause symptoms (e.g., symptom burden, as well as access to care and health literacy), so we considered it a potential analytic confounder. Our study was not designed or powered to address questions regarding MHT use, though we acknowledge this is an important area of investigation. Nonetheless, we now include post-hoc analyses to begin to address questions around MHT use on our outcomes of interest. We now report exploratory models stratified by history of MHT use and no history of MHT use, separately, in the aging cohorts testing associations of: (1) history of menopause symptoms with the menopause proteomic score, and (2) the menopause proteomic score with cognition and dementia risk. Generally, effect sizes were relatively similar between the two MHT groups, with no clear systematic differences between MHT users and non-users (Table S16). A caveat as noted above is that most of these stratified sample sizes were small, and we only had limited self-report data on MHT use history. Also, data on MHT use were not collected in ADNI so this could not be examined in that cohort. Future studies designed to test how MHT, and other hormonal and non-hormonal therapies moderate biological trajectories over endocrine transitions in women remain urgently needed.

Comment 12. It is interesting that no associations were seen with 'other' menopausal symptoms such as mood swings, depression and sexual dysfunction. The authors should provide clear and evidence-based information from prospective studies about why these were considered menopausal symptoms in the first place? Note that NIH (admittedly in 2005) concluded that only VMS and vaginal dryness were menopausal symptoms. I am not aware of any quality evidence that menopause at the average age is associated with depression (https://pubmed.ncbi.nlm.nih.gov/41371356/), mood swings (what does this even mean?). To my knowledge only one prospective study has measured sexual functon(SWAN https://pubmed.ncbi.nlm.nih.gov/19212271/ and POA) reports complex changes with sexual dysfunction in the minority.
This may explain the lack of association with biomarkers and 'other' menopausal symptoms.

Response 12. Thank you for the opportunity to clarify the rationale for the selection of menopause symptoms. We first acknowledge that the field's understanding of menopause symptoms is incomplete, and the full scope of possible symptoms women

may experience are still be delineated. However, the Menopause Society and the NIH's evidence-based MyMenoPlan.org, established by the National Institute of Aging in 2020, provides comprehensive reporting on currently established menopause symptoms. In WRAP, the menopause symptoms that female participants were asked to report cull from the established literature and included: hot flashes, sleep disturbances, mood swings, depression, night sweats, and sexual dysfunction. These symptoms were selected by WRAP investigators because they are among the most reported menopause-associated symptoms in midlife women. For example, reviews show that between 40-65% of women report menopause-associated sleep problems (PMID: 38916279), and evidence from multiple prospective and cross-sectional studies including meta-analysis shows increased risks for depressive symptoms and mood changes around the time of menopause (PMID: 38642901; 30182804; 21961723; 38642901). We will also note that the study the reviewer pointed to also shows a relationship between the menopause transition and increased risk for incident depression and anxiety in >10k participants from UKB (PMID: 41371356). While we agree with the reviewer that the term "sexual dysfunction" may be imprecise and encompass a complex and heterogenous range of symptoms, declines in libido and increases in sexual distress and pain during sex are consistently reported across the menopause transition in population-based and prospective studies (DOI: 10.1016/j.lanogw.2025.100024; PMID: 15287158). These citations have been added to the manuscript (Extended Data, p.4):

> "Menopause symptoms reported included hot flashes, night sweats, sexual dysfunction, problems sleeping, mood swings, and depression – which together are among the most common and distressing menopause symptoms observed in midlife women[11–17] – plus total symptom count."

Comment 13. The CCL11 association with cognition was interesting. However, calling this 'cognitive decline' seems alarmist and unjustified by the findings reported. Reviewing the literature on CCL11, I was unconvinced by evidence that this is a marker of cognitive change/decline anyway (https://pubmed.ncbi.nlm.nih.gov/34320382/). Perhaps I am missing something?

Response 13. Thank you for raising this point. Given additional analyses that did not show consistent associations of CCL11 with cognitive outcomes across newly added aging cohorts (i.e., ADNI, UKB; Tables S12-13; Fig.6F), the discussion of CCL11 in the context of cognitive decline has been removed.

Comment 14. DISCUSSION I think the statement 'did not associate with age' is too strong given their data. Should be toned down.

Response 14. We have moderated the claim as follows (p.10):

> "Menopause-related proteomic shifts tracked more strongly with hormones than with age."

Comment 15. Similarly, comment on line 396 about 'worse cognitive outcomes and elevated AD biomarker". I think one biomarker was elevated and suggest replacing the term 'worse cognitive outcomes' with a more realistic reflection of what the data actually showed. They looked very hard for biomarkers that differed depending on reproductive stage - using primarily cross-sectional cohorts. It is incorrect to conclude that this indicated a key transition in midlife health, since women were not followed prospectively.

Response 15. Thank you for this feedback and for the opportunity to more accurately summarize our conclusions. We have moderated these statements and updated them to reflect the addition of new data, as below:

> "Further, in four independent cohorts of older postmenopausal women, menopause proteomics consistently associated with poorer cognitive outcomes, suggesting that menopause-related biological shifts may remain relevant years to decades post-menopause. Collectively, our findings highlight potential biology linking menopause to risk and resilience in cognitive aging." (p. 10)

Reviewer #2: proteomics

This is a very well written important manuscript outline several circulating proteins that are associated with menopause (e.g., spontaneous, associated with vasomotor or neurologic symptoms (e.g., brain health)) in various cohorts. Interesting they also show age was not a factor. Tables were well organized and easy to read. The issues are minor but important for clarity. All issues should be easily addressed by the authors.

Comment 1. Comparisons are made in cohort between data from sample types (serum/plasma) which cause the same assay to behave differently for specific analytes. If there is published data (not from manufactures) around which analytes the authors measured differ this needs to be cited and the addressed by the authors. If not, then this needs to be included as a potential analytical confounder for the study.

Response 1. Thank you for the very positive feedback and for the constructive suggestions. We agree that sample matrix is an important consideration in our study (see also Reviewer 1, comment 3). Assay matrices (i.e., plasma vs. serum) were selected based on sample/data availability. The NULISA CNS assay is new but recent data report comparable performance of this assay in serum and plasma (PMID: 42000813; now cited in the article). This citation shows high target detectability (>95% in both serum and plasma) and moderate-to-strong correlations for the vast majority (84%) of the ~120 targets measured, including all 16 identified menopause proteins. Serum may show slightly stronger inflammatory signals and slightly weaker neurodegenerative signal relative to plasma. This data supports the use of both plasma and serum samples for NULISA proteomics as in our study. Further, our ability to both replicate and detect consistent relationships with the menopause proteomic signature

across multiple independent cohorts, sample matrices, and even analyte platforms support the high rigor of the targets we have identified. Nonetheless, we acknowledge challenges of sample matrix differences as a potential limitation (p. 12):

> "Furthermore, while serum and plasma appear to perform comparably on the NULISAseq CNS panel,[43] matrix differences may influence protein measurements making cross-cohort comparison more challenging."

Comment 2. A few analytes there measured by different assay vendors. In addition to the physical technical aspects of the platform, the vendor will/could use different antibodies and thus the assay can have different LLOD and LLOQ making comparison challenging. It would be helpful to have either LLOD/LLOQ provided for these analytes as well as for all key analytes discussed as part of the proteomic signature. This is specifically an issue with Alamar (for example ptau 181 LLOD seems to be only listed in a poster) as Quanterix has most published. This is important for numerous reasons including as many assays can provide a "value" but yet the values may not be above LLOQ. In this case, the value (data) is not quantifiable and should not be used. This can differ for different assays (and sample type).

Response 2. Thank you for raising these important considerations. NULISAseq assays have plate-specific limits of detectability (LODs) calculated using a standard algorithm and negative control samples run in quadruplicates. We examined LODs for all cohorts included that used NULISA (UCSB menopause cohort, BrANCH, ADNI, WRAP) and calculated median detectability for each protein, defined as the percentage of samples in which the protein quantification is above the LOD for that target. Consistent with other NULISA CNS studies (e.g., https://www.biorxiv.org/content/10.1101/2025.06.10.658994v1.full), we have now removed proteins with <50% detectability from analysis, resulting in the removal of 8 proteins from the main UCSB menopause cohort, and 1 protein from ADNI. These steps are described in more detail in the Extended Data (p. 1-2):

> "Plate- and sample-level QC metrics (e.g., coefficient of variation (CV), detectability) were used to assess assay performance. The limit of detectability (LOD) is determined as the mean plus three times the standard deviation of the exponentiated (i.e., anti-log-transformed) normalized counts for the negative control samples on the plate, which are run in quadruplicates. Detectability is then calculated as the percentage of samples in which the quantification is above the LOD for that target. […] In the UCSB HAS, 8 proteins (i.e., UCHL1, SNCB, PTDP43409, PTN, pTau217, OligoSNCA, IL1B, and NRGN) had median detectability of <50%, so were removed from analyses following previous work with the NULISA panel.[3] In all other NULISA cohorts (i.e., BrANCH, ADNI, WRAP), our analyses were restricted to the 16 menopause proteins identified in the UCSB HAS. All 16 of these menopause proteins were detectable (≥50%) in BrANCH and WRAP and all except SNAP25 were detectable (≥50%) in ADNI. SNAP25 was removed from analyses in ADNI."

Furthermore, a full list of proteins included in analysis in each cohort is now provided in Table S17.

Response 3. It was not clear if a single sample was run (or duplicates) and what QC was used for the NULISA CNS panels to ensure technical performance of the assay.

Response 3. Additional processing and QC details for the NULISA panels have been added to the Extended Data (p.1-2):

> “Samples were randomized (non-stratified) across plates and run in singlet, per previously established procedures for NULISA assays.[1,2] […] Plate- and sample-level QC metrics (e.g., coefficient of variation (CV), detectability) were used to assess assay performance. […] In all cohorts, QC metrics were in acceptable ranges, including for both intra-plate internal control CV (UCSB HAS: 15.0%; BrANCH: 7.3%; ADNI: 5.9%; WRAP: 0.01-7.5%; all within maximum threshold of 25%) and inter-plate control CV (UCSB HAS: 4.0%; BrANCH: 8.6%; ADNI: 11.8%; WRAP: 0.29-5.6%; all within maximum threshold of 15%).”

Comment 4. The authors need to add in how samples were randomized and how they handled batch corrections for each cohort (only mentioned for one assay) and missing data (was imputation used).

Response 4. We have also added details on randomization, batch corrections, and missing data to the Extended Data (p.1-2):

> “Samples were randomized (non-stratified) across plates and run in singlet, per previously established procedures for NULISA assays.[1,2] Batch (plate) effects were mitigated using the standard NULISA Protein Quantification (NPQ) protocols and algorithm (as previously described),[1] including hierarchical normalization that consists of internal spike-in controls for well-level normalization, pooled inter-plate controls to harmonize protein measurements across plates, and negative controls to estimate background and limits of detection. Protein concentrations are reported in NPQ units, which are computed by normalizing the relative concentration of each analyte using internal and inter-plate controls and $\log_2$-transforming the normalized values. […] There were no missing values on any examined proteins in the UCSB HAS cohort. No imputation was used for NULISAseq data, and analyses in other NULISA cohorts (i.e., BrANCH, ADNI, WRAP) were restricted to participants with non-missing values on all menopause proteins.”

Please note that to address reviewer feedback and the addition of new data (i.e., ADNI), we have refocused the aging analyses and now only include cognition/incident dementia as outcomes. We no longer use the Quanterix and Lumipulse plasma p-tau/AB42 assays in WRAP and BrANCH as study outcomes and only include these to characterize the biomarker status of participants (i.e., amyloid-positivity, for demographics). As such, we only include detailed technical and QC data for the

proteomics assays used as main outcomes (i.e., NULISAseq in UCSB, ADNI, WRAP, BrANCH; Olink in UKB).

Comment 5. Although described in the text a table outlining all cohorts, assays performed, and sample type would be helpful.

Response 5. We agree with this suggestion and have added the table to the supplement (Table S17).

Comment 6. The NULISA CNS panel has more proteins than 120. It is confusing which assay was used in the various cohorts and which (~120, 122 and 131). Please ensure that you state the specific assay and then list in supplement which protein comprised which assay. As well, please state whether there was a need to normalize across these different assays.

Response 6. Thank you for the opportunity to clarify. While the NULISA panel is called the "CNS Disease Panel 120" by the manufacturer (Alamar), it is correct that the various cohorts have different numbers of proteins. This is because the NULISA panels can be customized using additional targets spiked into the standard panel and the "CNS panel" continues to evolve as new targets are identified. We now explicitly state the number of targets measured in each cohort in the methods (p.14):

> "Serum (menopause cohort) or plasma (aging cohorts, see below) was analyzed on the NULISAseq CNS panel to measure 127 proteins in UCSB HAS, 131 in ADNI, 132 in BrANCH, and 122 in WRAP using the automated Alamar Argo HT workflow (Extended Data)."

As mentioned above in Response 5, we have also added a table to the Extended Data listing each cohort, assay, and protein comprehensively (Table S17).

We also now state explicitly that we did not normalize across cohorts/assays in the Extended Data (p.2):

> "Given all analyses examined proteomic outcomes within cohorts separately, we did not normalize protein levels between cohorts."

Comment 7. The UK biobanks looked life menopause cohort on NULISAseq with proximity extension assay (PEA) Olink" but this is not correct. The cited paper (Ref 25) only used Olink and this is not equivalent to NULISASeq). Ref 25. NULISAseq uses a proximity ligation assay (PLA) while Olink uses proximity extension assay (PEA).

Response 7. We apologize for the confusing wording of this passage previously. We have revised for clarity as follows (p. 14):

> "To evaluate whether spontaneous menopause proteomic shifts were robust to cohort and proteomic platform, we compared spontaneous menopause protein

> effect sizes detected in the midlife menopause cohort with NULISAseq proteomics to menopause protein effect sizes detected in a large cohort of 2,814 women from UK Biobank with cross-sectional proximity extension assay Olink proteomics (Extended Data).[48]"

Comment 8. I am not sure the title of the paper is quite accurate. Blood-based proteomic signatures of spontaneous menopause: Implications for later-life brain aging and Alzheimer's disease risk. The blood assay used are not conclusive for AD and perhaps other wording can be used to better reflect the interesting and important findings.

Response 8. We agree and have updated the title to one that we believe better reflects the content and findings of the paper: "Blood proteomics of menopause map to brain aging and dementia risk." Given that we now focus the aging analyses on later-life cognition/cognitive decline as well as incident dementia in the UK Biobank, this new title accurately captures the outcomes measured and reasonable conclusions that can be drawn from the data presented.

<u>Reviewer #3: AD</u>

The study addressed the question of which biofluid proteins are altered in association with menopause in women and whether those changes are associated with biomarkers of AD pathology in elderly women.
In a prospective study, NULISA-based protein levels were cross-sectionally compared between women who were either pre-, peri-, or post-menopausal. Results showed that a number of proteins differed between pre- and post-menopausal women at a nominal significance level, and a subsequently derived PCA score encompassing these selected proteins (i.e. menopausal protein signature) showed step-wise differences across pre-, peri-, and post-menopausal women. FSH and estradiol levels were associated with the menopausal protein signature and with some menopausal symptoms (night sweats, vaginal dryness).
In validation cohorts using already existing datasets of OLINK-derived plasma proteomics in age-matched pre- vs post-menopausal women from the UK Biobank, the alterations were confirmed, although a large number of other proteins also seemed to be altered.
In elderly women from two different cohorts (WRAP, Branch), higher protein signature scores were associated with a higher plasma p-tau/Ab42 ratio and lower cognition, although domain-specific associations could not be replicated.
Overall, the study addresses an important research gap in understanding the biological underpinnings of the menopause-related increase in AD risk. The combination of a prospective study with multiple validation samples is a strength. Also, several sensitivity analyses were conducted to exclude major confounds. Still, some concerns remain.

Comment 1. The establishment of the protein signature is based on a relatively small sample, and the selection of proteins is based on nominal p-values. PC1 captured less

than 25% of the variance in the selected proteins. Among the selected proteins, associations with the AD biomarkers and menopausal symptoms were often present only in a small subset when analyzed protein by protein. Is this truly a menopausal protein signature, or simply a statistical strategy to avoid the problem of multiple testing and thus low statistical power? The latter would be legitimate, but the interpretation would be different.

Response 1. We sincerely thank the reviewer for the feedback on our manuscript and for the thoughtful considerations raised. We agree that our study did not holistically identify all possible proteomic shifts of menopause (panel was limited to ~120 proteins) and we were limited by a small sample size and lack of longitudinal follow up in our menopause discovery cohort (also see Reviewer 1 responses 6 and 7). As such, we use PC1 not as a true menopause protein signature but as a statistical strategy to reduce data dimensionality/noise introduced by the protein measures, and to capture menopause-related biological changes observed in our data with a single measure that mitigates the problem of multiple comparisons. To clarify this, we have renamed PC1 as a "menopause proteomic composite" rather than a "signature." This rationale is now stated clearly on page 5:

> "To reduce data dimensionality and noise, more holistically capture biology represented, and mitigate multiple comparisons, we performed principal components analysis (PCA) on these 16 targets to estimate a menopause proteomic score that summarizes these top menopause-related changes."

We further acknowledge that the menopause score does not represent the full range of menopause-associated biology in the discussion (p. 12):

> "While we used a composite score to summarize menopause-associated biological signals observed on ultrasensitive NULISAseq in our deeply-phenotyped cohort, the menopause proteomic score does not represent the full breadth of menopause-related biology detectable in circulation, as evidenced by the widespread proteomic shifts seen in the UKB Olink analyses. That is, NULISAseq CNS captures only ~120 proteins involved in neurologic aging, while other high-throughput assays may allow expanded discovery."

Comment 2. The authors conclude that the menopausal proteins were not associated with age (e.g. line 394). The reviewer appreciates that sensitivity analyses were conducted in an age-restricted subset to confirm the major findings. Still, it is important to understand whether these protein changes are sensitive to age-related changes.
In the main analysis, the authors test in a partial correlation analysis whether the protein signature is associated with age after partialling out menopause status. However, given that menopause status probably reflects age differences, that statistical design is not a rigorous test of potential associations with age.
Can the authors test whether 1) there are associations with age without controlling for menopause status and 2) menopause differences after partialling out age effects?
Also, given that younger age at menopause onset has been associated with higher

dementia risk in some studies (e.g. Dobson et al. Age and Ageing, 2024), age may play a modulating role rather than simply being a nuisance variable.

Response 2. We agree that it is important to understand whether the reported protein changes are sensitive to age-related changes, and have added the requested analyses, which are now described in the Results (p. 5-6) and shown in Figure 1E and Table S4. In brief, we found that there were significant associations between some of the menopause proteins and age in women when unadjusted for menopause status. This is expected given that menopause stage and age are inherently confounded. However, in multivariable models considering both age and menopause status, age no longer associated with menopause scores. We also compare the unadjusted effect sizes of age and menopause stage on menopause scores to contextualize potential effects of age vs. menopause. Here, we show that the effects of menopause stage are notably stronger than those of age. Finally, to address the potential modulating role of age, which we agree is an important consideration, we test whether there were interactive effects of age and menopause status on menopause scores. There was no evidence of interactive effects in these models. Together, these findings suggest that menopause stage has statistically stronger and larger effect sizes than chronological age with the identified menopause proteins. These findings are described in the text as below (p. 5-6):

> "In women, age was significantly associated with higher menopause proteomic scores (cor=0.36, *p*=.001). However, this association with age was no longer significant and reversed directionality after adjusting for menopause stage (β=-0.14, 95% CI=-0.31, 0.03, *p*=.11). Unadjusted linear estimates showed a stepwise effect of menopause stage on the proteomic score. Relative to premenopausal, perimenopausal status was associated with a 1.36-unit (*SD*) increase in menopause scores, and postmenopausal status with a 3.25-unit increase. By contrast, each year of age was associated with a 0.24-unit higher menopause score. To assess the potential modulating role of age, we also tested interactions between menopause stage and chronological age on the menopause score, which were not significant (post [vs. pre]*age: β=0.003, 95% CI= 0.44, 0.45, *p*=.99; peri [vs. pre]*age: β=0.17, 95% CI=-0.24, 0.57, *p*=.42). We computed the same composite score in men and found age did not correlate to the menopause score in men (cor=-0.02, *p*=.89). Overall, these findings suggest that the observed proteomic changes were driven more by menopause stage than chronological age in women."

For individual menopause proteins, we also present associations with age in both women (unadjusted and adjusted for menopause stage) and in men in Figure 1E.

Comment 3. In general, the control for potential confounding variables is rather sparse. Given that immunity-related proteins (e.g. CCL11) were highly represented among the proteins loading on the PC1 score, variables that may influence systemic inflammation (e.g. vascular conditions, smoking, anti-inflammatory medications) should be controlled for if available.

Response 3. We agree that it is helpful to know if menopause-associated proteomic shifts (and their possible downstream cognitive sequelae) are robust to adjustment for common health conditions and risk factors that may affect systemic inflammation and the proteome more broadly. We have added sensitivity analyses adjusting for several key age-related vascular comorbidities/risk factors, including smoking, body mass index (BMI), high blood pressure, high cholesterol, and metabolic dysfunction/diabetes, as available in each cohort and as detailed in the Extended Data (p. 6) and Tables S15-16:

> "In all cohorts, we also re-estimated models after adjusting for cardiometabolic comorbidities and common health-related risk factors that may influence the proteome, per available data in each cohort. These covariates included: UCSB HAS: BMI, smoking history, and self-reported histories of high blood pressure, high cholesterol, and diabetes; ADNI: BMI, smoking history, systolic blood pressure, and diastolic blood pressure; BrANCH: BMI, smoking history, systolic blood pressure, diastolic blood pressure, hbA1c, and LDL cholesterol; WRAP: BMI, smoking history, systolic blood pressure, diastolic blood pressure, fasting glucose, and LDL cholesterol; UKB: BMI, smoking history, systolic blood pressure, diastolic blood pressure, hbA1c, and LDL cholesterol. All analyses yielded similar results to main models (Tables S15-16)."

Comment 4. Related to the above: Given that previous studies reported an APOE genotype x sex effect on tau markers, APOE may also play a role in the observed proteome alterations.

Response 4. Thank you for this suggestion. We have added exploratory analyses testing the potential moderating effects of *APOE4* carriage in both the spontaneous menopause and aging analyses. These are mentioned briefly in the main text (p.10) and detailed in the Extended Data (p. 7):

> "In both the UCSB HAS and aging cohorts, we finally performed exploratory analyses testing effect modification by *APOE4* genotype (i.e., carrier vs. non-carrier). *APOE4* carriage was inferred using NULISAseq serum levels of the APOE4 protein in the UCSB HAS cohort (Fig.S8) and was determined using genetic data for the single-nucleotide polymorphisms rs429358 and rs7412 in the aging cohorts (i.e., ADNI, BrANCH, WRAP, UKB). These showed largely null effects, with the exception that *APOE4* carriage significantly exacerbated associations of menopause proteomic scores with global cognitive decline in ADNI (Tables S15-16; Fig.S7)."

Of note, in the newly added ADNI cohort, there was a significant three-way interaction between menopause proteomic scores, *APOE4* carriage, and time on longitudinal global cognitive decline, such that associations of higher menopause proteomic scores with cognitive decline were larger magnitude in *APOE4* carriers relative to non-carriers. This is shown in Figure S7 and below:

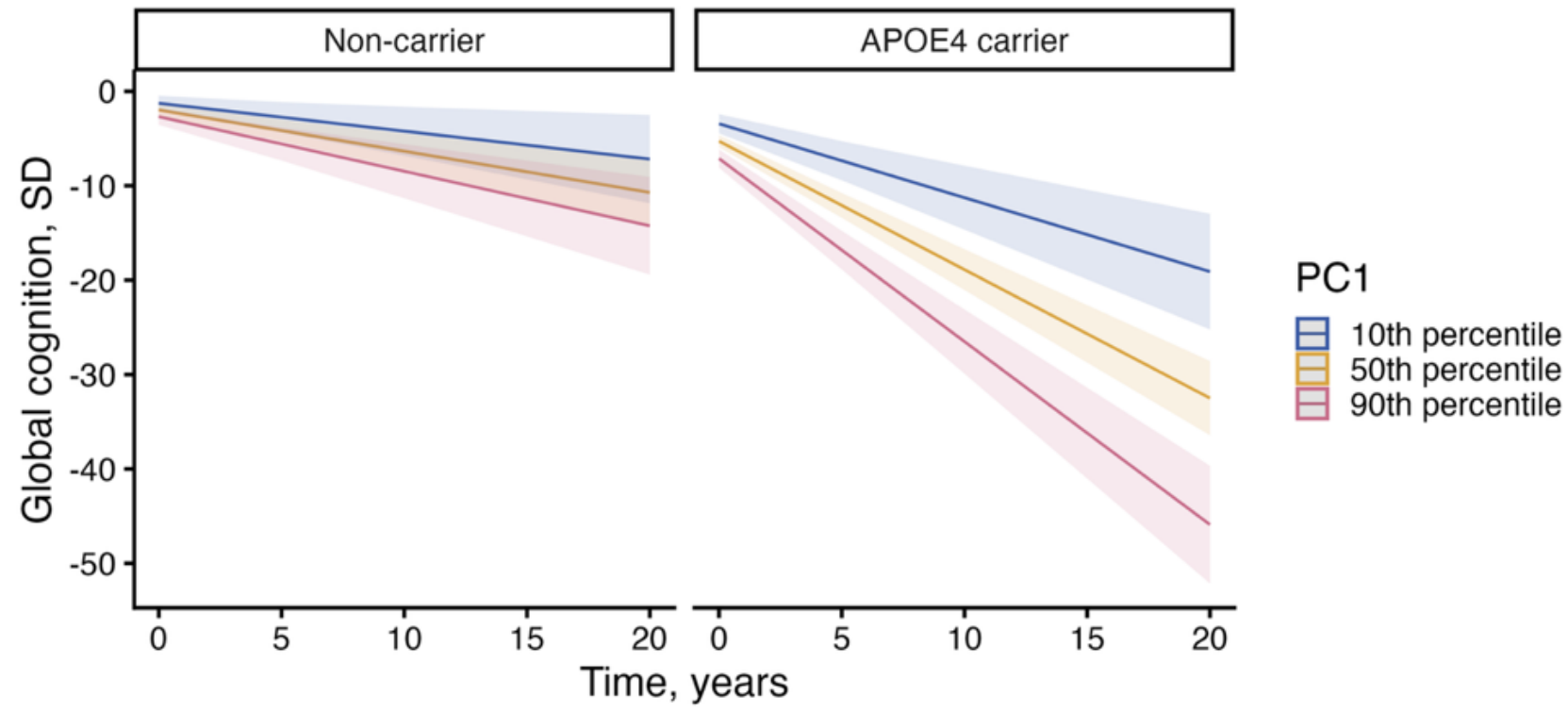

Comment 5. For testing associations between the PC1 protein signature scores and AD plasma markers (p-tau181/Ab ratio), p-tau231 should always be excluded from the signature, not only in sensitivity analyses, to avoid circularity.

Response 5. Thank you for this suggestion. We agree that these analyses were somewhat circular given that p-tau isoforms were used both in the menopause composite score and as later-life aging outcomes. To address this, we now focus our aging analyses exclusively on later-life cognition/cognitive decline and incident dementia, which we believe increase the clinical relevance and interpretability of our findings.

Comment 6. The demographic, clinical, and biomarker characteristics of the WRAP and Branch cohorts should be described in the main body of the article. What were the biomarker and cognitive status of these participants, rather than functional status? MCI patients may still be functionally normal.

Response 6. This information has been added to the main text as below (p. 9):

> "In longitudinal data with an average 5-year follow up from the Alzheimer's Disease Neuroimaging Initiative (ADNI; *N*=673; 54.3% mild cognitive impairment [MCI]/dementia; 49.0% amyloid-positive) and UCSF Brain Aging Network for Cognitive Health (BrANCH; *N*=100; 1% MCI; 21.7% amyloid-positive) […] In cross-sectional data from WRAP (*N*=93; 15.0% MCI/dementia; 64.5% amyloid-positive) …"

Please note that for the UK Biobank, while we did confirm that no participants in our analytic sample had a prior registered diagnosis of dementia (Extended Data), information on baseline cognitive status and amyloid positivity is not available in this dataset, so is not reported in the manuscript.

Comment 7. The authors mention that 9 out of 13 proteins from the discovery cohort also differed between pre- and post-menopausal women in the UK Biobank, suggesting high reproducibility. However, Figure 6B suggests that most proteins showed either up- or downregulation in the UK Biobank. Could the authors compute the likelihood that 9 out of 13 proteins from the protein signature would also show elevations just by chance,

given the proportion of altered proteins from the Olink assay in the UK Biobank? The concern is that, due to the high number of menopause-related protein alterations in the UK Biobank, most proteins from the signature may appear altered just by chance, along with many others.

Response 7. We thank the reviewer for raising this point. We agree that a substantial proportion of proteins differ between pre/peri- and postmenopausal women in the UK Biobank, which we interpret as evidence of genuine and widespread proteomic shifts associated with menopause. These included 9 of 13 available proteins that showed statistically significant and directionally concordant associations with menopause stage in both cohorts. We agree that this overlap alone should not be interpreted as evidence of a defining menopause signature score (see also Response 1). However, our assertion is not that these proteins constitute a uniquely specific menopause signature, but rather that they represent a reproducible subset of this broader menopause-related biological signal. This is supported by the concordance of effect sizes and directionality across both cohorts (Figure 4E).

To further address the reviewer's concern, we used Fisher's exact test to test whether the observed overlap of elevated proteins exceeds chance, given the background proportion of UKB proteins that show upregulation across the menopause transition. We found that the proportion of upregulated proteins in both UCSB and UKB was greater than expected by chance (OR = 3.6, Fisher's exact p = 0.025, corresponding to ~1.8-fold enrichment). These results further support that the replicated menopause-associated proteomic shifts are unlikely to be spurious and likely reflect biologically meaningful signals.

Comment 8. Do the protein alterations have any predictive value? This could potentially be assessed in the UK Biobank. Do women with higher protein signature values have a higher risk of (AD) dementia?

Response 8. We thank the reviewer for this suggestion and have added these analyses to the manuscript (p.9; Figure 6E and below):

> "Finally, in the UKB (*N*=11,059), we examined whether menopause proteomic scores associated with risk for incident dementia over a mean (*SD*) of 15.7 (2.84) years of follow-up. Higher menopause scores were associated with greater risk for AD dementia (HR=1.15, 95% CI=1.01, 1.31, *p*=.04, *N*=219 events; Fig.6E), but not frontotemporal (HR=0.90, 95% CI=0.59, 1.36, *p*=.61, *N*=23 events), vascular (HR=1.15, 95% CI=0.91, 1.47, *p*=.25, *N*=65 events) or all-cause dementia (HR=1.08, 95% CI=0.98, 1.19, *p*=.13, *N*=387 events)."

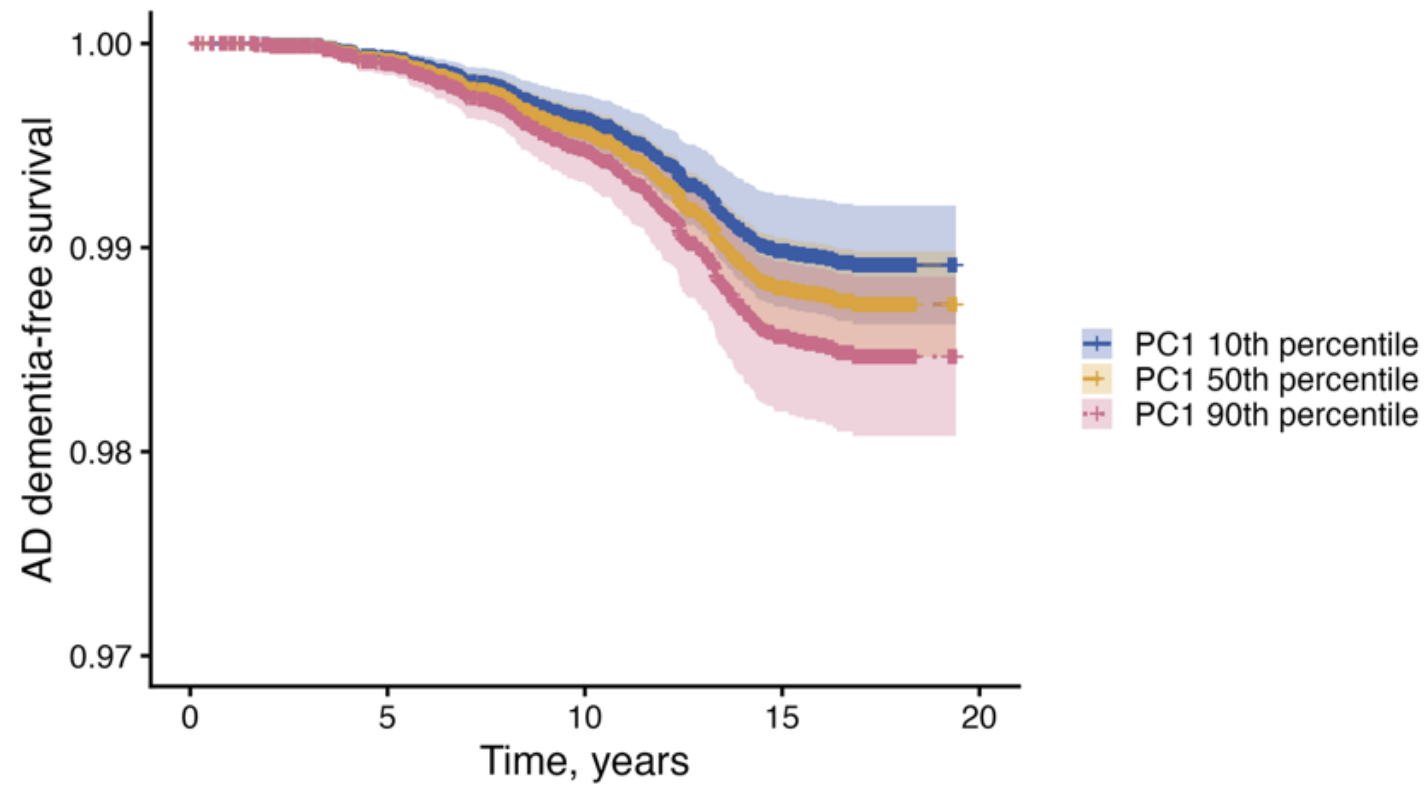

Methodological details for these analyses are described in the Extended Data (p.4):

> "***UK Biobank (UKB; aging cohort 4):*** To assess whether menopause-related proteomic shifts predict risk for dementia, we used data from *N*=11,059 women ≥50 years old in the UKB with non-missing values for all 13 menopause proteins measured on Olink at study baseline. Participants were free from known dementia at baseline and followed for a mean (*SD*) of 15.7 (2.84) years. Consistent with prior work, incident dementia cases were algorithmically defined using a combination of information in international classification of diseases (ICD) diagnosis codes in linked primary care, hospital inpatient, and death registry data, for all-cause dementia (codes A81, F00 to F03, F05, F10, G30, G31 and I67), alongside Alzheimer's disease (F00 and G30), vascular dementia (F01 and I67) and frontotemporal dementia (F02 and G31).[5] Participants were censored at first dementia diagnosis, death, or September 2025, representing the most recent update of algorithmically defined dementia outcomes in the UKB."

While we did observe statistically significant increases in AD dementia risk with higher PC1 scores, in the discussion, we acknowledge that effect sizes were small (p.11-12). Further, in the UKB, there were only proteomics data available for 13 of the 16 menopause proteins, so the incident dementia analyses were performed using a modified version of the menopause proteomic score that did not include p-tau231, BACE1, or IL12p70.

Reviewer #1:

Comment 1. The authors have largely addressed the issues raised. The addition of further cohorts is welcome.

Response 1. We thank the reviewer for their feedback and for their thoughtful suggestions, which we believe have substantially strengthened the manuscript.

Comment 2. I am unhappy about the suggestion that cognitive symptoms (largely 'brain fog) over the menopause transition is somehow related to cognitive decline which is then related to dementia. This is a very long bow and the data do not support these associations. As well as being inaccurate, this kind of conflation risks alarming women who may be concerned that cognitive symptoms at menopause indicate an increased risk of dementia. As the authors note, objective measures of cognition at menopause do not fall outside the normal (impaired) range. That makes them normal. The authors have made interesting and important observations and do not need to artificially inflate these by over-calling their findings, especially when this may do harm.

I suggest that the language around cognition is clarified throughout the paper. For example "cognitive decline" is not an appropriate term for changes in cognitive function within the normal range. Please be mindful about accurate language to describe your findings.

Response 2. We appreciate these sentiments and agree that it is very important to carefully communicate findings to ensure it is not implied or misconstrued that menopause (or its downstream biological sequelae) leads to dementia. To address this, throughout the manuscript, we have removed the term “cognitive decline” as it pertains to our findings, to avoid the terminology that could be associated with clinically significant impairment thresholds. Instead, we describe our cognitive findings as “cognitive changes”. We have also carefully checked and revised (where relevant) our wording in the manuscript for instances where the results or interpretation could be misunderstood in this way. We hope that these revisions promote clear and accurate communication of our findings.

More broadly, we interpret our data as suggesting that the menopause transition may be a vulnerable window (“stress test”) for brain health. Currently, it is unknown if women who experience specific menopause symptoms, like cognitive changes, are at elevated risk for brain changes in later life. Similar to pregnancy in which women who experience preeclampsia are at a higher risk for stroke and vascular dementia in later life, it is plausible that these symptoms could be useful behavioral markers for future risk. This is an important future line of inquiry. Regardless, we strongly agree that given the lack of current data, it is critically important to communicate the boundaries of what we know – and we do not believe menopause “causes” dementia.

Comment 3. Similarly, I was unconvinced by the broad statement about menopausal symptoms being "brain related". It is well established that VMS arise from the

hypothalamus. Sleep and mood are also brain related but are common independent of menopause.

Response 3. Thank you for this comment. We introduce the neural basis of most menopause symptoms as evidence that the brain is, in part, an endocrine organ, and to demonstrate its acute responsiveness to the neuroendocrine changes induced by menopause. We believe that this positions menopause as a neurologic (and indeed, systems-level) biological transition that may be relevant for aging trajectories and later chronic disease risk. However, we appreciate that menopause symptoms could be impacted by both brain-mediated and non-neurologic factors. This has now been acknowledged in the discussion:

> "While vasomotor symptoms originate in the hypothalamus, these symptoms are multi-factorial and can be impacted by both neurologic and non-brain physiologic and contextual factors.[10,11]" (p. 12)

Comment 4. Similarly, the BMS statement that MHT does not restore estrogen concentrations to premenopausal levels seems rather odd. Postmenopausal women have physiologically lower estrogen levels than premenopausal women. There is no clinical evidence that "restoring" levels to those of premenopause is beneficial and consistent evidence that it may do harm (VTE, stroke etc). Also, "restoring" estrogen means adding progesterone (for most women) which is unphysiological in postmenopausal women and confers additional risk (breast cancer)

Response 4. We appreciate these points. At a high level, we wanted to communicate that the lack of clear benefit or protection of MHT for dementia does not negate asking the question if endocrine changes of menopause influence the brain. Nonetheless, to avoid speculation, we do not make any claims about whether hormone therapy restores the premenopausal endocrine environment in the manuscript.

<u>Reviewer #2:</u>

Comment 1. The authors have carried out an excellent revision. They have addressed all concerns within the manuscript providing information needed to clarify the analysis they have done. I still remain surprised that serum vs plasma essentially correlated but am happy with how the authors deal with this concern.

Response 1. We sincerely thank the reviewer for all their helpful feedback and suggestions to strengthen this work.

<u>Reviewer #3:</u>

Comment 1. The authors have adequately addressed all concerns. My only suggestion at this point is that in the newly added Figures 6B and C, data points (spaghetti plots)

instead of error bars only are shown. This would allow to better appreciate the spread of the data. Figure 6B shows that error bars do not always reflect the full extent of variability in the data.

Response 1. We greatly appreciate the reviewer's insight and thank them for their suggestions. We have added spaghetti plots to Figures 6B and C (now Figures 5B and C).
